# The changes of microbial community and flavor compound in the fermentation process of Chinese rice wine using *Fagopyrum tataricum* grain as feedstock

**DOI:** 10.1038/s41598-019-40337-8

**Published:** 2019-03-04

**Authors:** Qing Ren, Leping Sun, Huijun Wu, Yousheng Wang, Zhiwei Wang, Fuping Zheng, Xin Lu, Jialiang Xu

**Affiliations:** Beijing Advanced innovation Center for Food Nutrition and Human Health, Beijing Technology and Business University, Beijing, 100048 China

## Abstract

Chinese rice wine (CRW), a unique wine species, has a long history in China. *Fagopyrum tataricum* grain is a kind of high-quality grain with function in health care. The production of CRW wine with *F*. *tataricum* grain is beneficial to the development of new rice wine products. The flavor compounds and microorganisms in *F*. *tataricum* grain rice wine were studied. One hundred and seven volatile compounds (including 11 kinds of pyrazines that were rarely detected in wine) were detected and eight organic acids were measured. The microecological diversity in the fermentation process of *F*. *tataricum* rice wine was studied. It was found that *Bacillus* was the main bacterial genus, and the unclassfied_O_*Saccharomycetales* was the main fungi. Correlation analysis between microorganism and flavor compound shown there are 838 correlations. A total of 108 microbial genera maybe participate in the formation of flavor compounds. In addition, fourteen genera included unclassified_O_*Saccharomycetales*, *Lactococcus*, *Pediococcus*, *Aspergillus*, *Cladosporium*, *Cochliobolus*, *Sporidiobolus*, *Pichia* and *Saccharomycopsis et al*. were screened as functional significant microbiota and built correlation with flavor compounds. This work provides a perspective for bridging the gap between flavor compound and microbial community, and advances our understanding of mechanisms in *F*. *tataricum* rice wine fermentation.

## Introduction

Chinese rice wine (CRW) is a Chinese traditional alcoholic beverage. It has been popular in China for thousands of years because of full-bodied aroma, soft taste, high nutritional value and low alcohol content^1,2^. CRW fermentation usually uses grain as feedstock. Wheat Qu, as a starter for CRW production, contains a variety of microorganisms and their metabolites. Wheat Qu has a compound enzyme preparation to provide a variety of enzymes, which is needed for the CRW fermentation process. Therefore, the quality of Wheat Qu has a great impact on CRW^3^. Additionally, yeast and amyloglucosidase were added separately to pre-saccharify feedstock for increasing alcohol. The fermentation of CRW needed two processes: saccharification of starch and fermentation of sugars. Starch can be saccharified to glucose and other sugars by amylase and amyloglucosidase. Meanwhile, protein can be hydrolyzed by proteases and carboxypeptidases to peptides and amino acids. Then, yeast can utilize these nutrients for cell growth, fermentation and flavor formation^1^. Furthermore, with development of the CRW industry, the kinds of feedstocks for fermentation of CRW continue to expand^4^. In recent years, some scholars have begun to use *Fagopyrum tataricum* as fermentation feedstock to study and optimize the fermenting process of CRW^5,6^. *F. tataricum* grain, which was a functional food material, have many advantages, such as high biological value proteins, high crude fiber, balanced amino acid composition, and high vitamin B1, B2, and B6^7,8^. In addition to rich nutrition, *F*. *tataricum* also has functions of lowering blood lipid and blood sugar, regulating myocardial activity, clearing free radicals and other physiological functions^9^. Studies have shown that *F*. *tataricum* CRW has the function of reducing blood lipid, blood sugar and blood pressure^10^. The use of *F*. *tataricum* as feedstock can not only use its rich nutrition and physiological activity to increase the variety of CRW, but also meet the consumer’s demand for low alcohol content^2^.

Flavor is an important feature of CRW. Alcohols, aldehydes, esters, acids, phenols and so on, contribute to the various volatile flavor formation. There are many reports describing the volatile and semi-volatile flavor compounds in CRW^11^. A total of 66 volatile compounds were identified in glutinous rice CRW that rice was pretreated by enzymatic extrusion or steam-cooked, in the meanwhile, the alcohols, esters, and aromatic compounds contribute to the various volatile flavor formation^12^. A total of 15 volatile compounds were detected and measured in the CRW from Shaoxing region, including 5 higher alcohols and 10 esters. Almost all of these compounds have already been detected in CRW^4^. It is well known that bacteria can produce many flavor compounds and determine the quality of CRW. However, there are few reports about how bacterial communities change in CRW, and most bacteria can only be detected by cultivation-dependent methods, such as cell culture and colony counting^13,14^. With the development of science and technology, Illumina-based metagenomic sequencing is widely used. Through this technique, Xie *et al*. revealed the community structure of Shaoxing rice wine at different fermentation times^15^. Liu *et al*. used metagenomics and found that the Jiannanchun koji microbial community composition is stable and mainly distributed in *Proteobacteria*, *Firmicutes* and *Actinobacteria*, which accounted for more than 95% of the microbial community^16^. However, studies on the microbial diversity and flavor of Chinese rice wine fermented with *F. tataricum* using the High-throughput sequencing (HTS) approach have not been reported yet.

With the development of ecological techniques, there are increasing studies which focus on microbes and metabolic phenotype correlation in the food fermentation^17,18^. Some statistic approach and models are applied to understand the role of microbiota and functions of the community in fermented foods. To associate the microbiota and volatile compounds in the fermentation of Daqu starter for Chinese liquor making, Pearson correlations coefficient (r) was calculated among the microbial genera and metabolites, and suggested that Lactobacillus were associated with esters, acids and alcoholsand Bacillus and Clavispora positively correlated with six pyrazines^19^. In addition, in the acetic acid fermentation (AAF) of Zhenjiang aromatic vinegar, bidirectional orthogonal partial least squares (O2PLS) was applied to predict the relationship between microbiota assembly and flavors datasets. Finally, a functional core microbiota was selected by comparison of the comprehensive importance of microbiota correlated with flavours^20^. In this study, we adopted Pearson correlations coefficient (r), correlation network and bidirectional orthogonal partial least squares (O2PLS) to predict relationship between microbial genera and flavor. Functional core microbiota were selected by comparison of the comprehensive significance of microbiota correlated with flavors in the fermentation process of CRW using *F. tataricum* grain as feedstock.

In this paper, the volatile flavor compounds were determined by HS-SPME and GC-MS. Bacterial diversity was analyzed using MiSeq sequencing of the 16S rRNA gene clone library. Fungal diversity was analyzed using MiSeq sequencing of the internal transcribed spacer (ITS1) region. The objective of this study was to identify the flavor compounds in *F*. *tataricum* grain CRW, and to predict the relationship between volatile compounds and microbial communities, and to estimate the functional significant microbiota by bioinformatics analysis. In order to produce high-quality CRW, we must make full use of the aroma producing microorganisms. So the analysis the relationship of flavor substances and microorganisms maybe provide some hints. This experiment provide theoretical support for future production of CRW by analyzing the microbial community dynamics and metabolic profile of CRW, predicting the association between microbes and flavors. In addition, *F. tataricum* as the feedstock can increase the variety of CRW and increase the added value of *F. tataricum*.

## Materials and Methods

### Sample collection

*F. tataricum* were collected in April 2017, in Zhangjiakou, Hebei Province. Fermentation was carried out in winery’s experimental tanks with a constant temperature of 28 °C after collecting the *F. tataricum*. The period of fermentation was 12 days. The samples were harvested every two days. During different fermentation stages of 0, 2, 4, 6, 8, 10, and 12 days, three parallel samples were collected each time. When sampling, the ferment was mixed evenly. The 300 g samples that at different locations in the pits were harvested into sterile bottles. A total of 21 samples of different fermentation days were collected. Each sample was divided into two parts: one part was centrifuged with 4000 r/min for 20 min and stored the supernatant at 4 °C immediately for the flavor test, and the other part was continues placed in sterile bottles and stored at −80 °C before DNA extraction. Dry ice was used to keep low temperature throughout the transportation process.

### Determination of Reducing Sugar, Alcohol, Acidity, Flavor Compounds and Organic Acids

The DNS method was adopted to determine the reducing sugar content with glucose as a reference standards. A 1.0 mL liquid sample was ten times diluted. Then, 1.0 mL of the dilution was shaken well with 1.0 mL of DNS solution and put in a boiling water bath for 5 min. When the mixture cooled to room temperature, it was diluted by water to a final volume of 10.0 mL, before absorbance at 540 nm was read^21^. The alcohol degree was measured by density bottle method. By taking 100 mL wine into a distillation flask for rotary evaporation, the distillation was stopped when about 95 mL of distillate was collected. After cooling the distillate to room temperature, the volume was made up to 100 mL by water in a measuring cylinder. The alcohol content was measured by density bottle. Acidity was measured by titration. 10 mL of wine was taken into a beaker, and 50 mL water without of carbon dioxide was added. After pH value was titrate to 8 with 0.1 mol/L NaOH, the volume of NaOH consumed was recorded. The acidity was calculate according to the formula below.

$${\rm{X}}=\frac{(V1-V2)\times c\times 0.090}{V}\times 1000$$

X: Acidity of the sample. The unit is g/L.

V1: The volume of NaOH consumed by the sample. The unit is mL

V2: The volume of NaOH consumed by the blank. The unit is mL

c: The concentration of NaOH. The unit is mol/L

0.090: The molar mass of lactic acid. The unit is g/mol

V: The volume of sample. The unit is mL.

Those methods all according to the standard of GB/T13662-2008 to evaluate the wine quality^22^.

The flavor compounds in the samples were analyzed by headspace solid-phase microextraction and gas chromatography mass spectrometry (HS-SPME/GC-MS). Semi-quantification of volatile compounds was performed using 2-octanol as the internal standard^23^. Each wine sample (8 mL) was placed in a 15 mL SPME glass vial together with 2.5 g sodium chloride and 150 μL 2-octanol (65.76 mg/L) . The sample was extracted by SPME fiber (50/30 μm DVB/CAR/PDMS) in water bath and ultrasonic wave for 45 min at 50 °C. After extraction, fiber was introduced into the injection port of the GC-MS system (at 230 °C for 5 min). Identification was carried out using a Shimadzua-QP2010 Plus-GC-MS. Each concentrated fraction was analyzed on a DB-wax column (30 m X 0.25 mm i.e., 0.25 µm film thickness). The carrier gas, helium, was circulated at 1 mL/ min in split-flow mode with a split ratio of 50/1. The oven temperature program was as follows: 35 °C (4 min), 5 °C/min to 150 °C (2 min), 3 °C/min to 210 °C. The injector and detector temperatures were 230 °C and the ion source temperature was set at 200 °C. The ion energy for the electron impact (EI) was always 70 eV. The chromatograms were recorded by monitoring the total ion currents in the 30-350 mass range. Data was collected in total ion mode. Semi-quantitative data of the flavor compounds were acquired with the following formula:

$${\rm{C}}({\rm{\mu }}g/{\rm{L}})=\frac{{\rm{Ac}}}{{\rm{Ais}}}{\rm{Cis}}({\rm{\mu }}g/{\rm{L}})$$

C: the relative concentration of flavor compounds;

Cis: the final concentration of internal standard in sample;

Ac: peak area of flavor compounds;

Ais: peak area of internal standard.

The organic acids in the samples were analyzed by HPLC. Each wine sample (5 mL) was placed in a tube and centrifuged at 10,000 r/min for 15 min, then filtered through a 0.45 mm microporous membrane. Chromatographic conditions were based on the method described by Ye *et al*.^24^. The determination of the organic acids was carried by HPLC. The separations were carried out on an Agilent 1260 Infinity II equipped with a 250 mm × 4.6 mm and 5 μm welch ultimate XB-C18 column. The column temperature was set at 30 °C. A mixture of phosphate buffer (0.01 mol/L (NH_4_)_2_HPO_4_) adjusted with a phosphoric acid solution to a pH of 3.0 was used as the mobile phase with a flow rate of 0.8 mL/min. The detection wavelength was 215 nm.

### DNA extraction, PCR amplification and Illumina MiSeq sequencing

Microbial DNA was extracted from the samples using a M4015-01E.Z.N.A.^®^ soil DNA kit (Omega Bio-Tek, Norcross, GA, U.S.A.). The final DNA concentration and purity were determined using NanoDrop 2000 UV-vis spectrophotometer (Thermo Scientific, Wilmington, USA). DNA quality was checked by 1% agarose gel electrophoresis. The primers for bacterial and archaeal 16 S rRNA genes and the eukaryotic ITS rRNA genes were provided by Majorbio Biotechnology Company. The V3-V4 hypervariable regions of the bacterial 16 S rRNA gene were amplified by polymerase chain reactions (95 °C for 5 min; followed by 25 cycles of 95 °C for 30 s, 55 °C for 30 s and 72 °C for 40 s with a final extension of 72 °C for 10 min) with primers 338 F (5′-ACTCCTACGGGAGGCAGCAG-3′) and 806 R (5′-GGACTACHVGGGTWTCTAAT-3′)^25^ using a thermocycler PCR system (GeneAmp 9700, ABI, USA). The ITS1 was amplified by PCR (95 °C for 2 min, followed by 30 cycles at 95 °C for 30 s, 61 °C for 30 s, and 72 °C for 45 s with a final extension at 72 °C for 10 min) with the ITS1F (5′-AxxxCTTGGTCATTTAGAGGAAGTAA-3′) and ITS2 (5′-BGCTGCGTTCTTCATCGATGC-3′)^26^ primers. PCR products were purified according to a previous study^27^. Purified amplicons were sent to the Majorbio Institute (Shanghai, China) for Illumina high-throughput sequencing on a MiSeq platform (Illumina, CA, USA).

### Processing of sequencing data

Raw fastq files were demultiplexed, quality-filtered by Trimmomatic and merged by FLASH using the following criteria: (i) the reads were truncated at any site receiving an average quality score < 20 over a 50 bp sliding window. (ii) Primers were exactly matched, allowing 2 nucleotide mismatches, and reads containing ambiguous bases were removed. (iii) Sequences whose overlap was longer than 10 bp were merged according to their overlap sequence^27,28^.

Sequences were clustered into operational taxonomy units (OTUs) at a similarity cutoff of 97% (version 7.1 http://drive5.com/uparse/) and further assigned to phylotypes from the phylum to the genus level using classify. Alpha rarefaction was performed in QIIME (version 1.7.0) using the Chao1 estimates of species abundance^29^, which is the observed species estimation of the amount of unique OTUs found in each sample, and the Shannon index^30^. The cluster analysis was preceded by PCA^31^. For the QIIME calculation, the beta diversity of the unweighted and weighted UniFrac distances was used for UPGMA clustering and principal coordinate analysis^32^. To identify the differences in the bacterial communities between the two groups, similarities were analyzed using the Bray-Curtis dissimilarity distance matrices.

The OTU abundance table was standardized by PICRUSt. According to the KEGG database, the KO, Pathway and EC information in the fermentation process was obtained, and the abundance of each functional category was calculated according to the OTU abundance. In addition, for the Pathway, 3 levels of information on the metabolic pathways were obtained using PICRUSt, and the abundance tables at each level were obtained.

The significance of correlations between the microbial genera and flavor compounds were tested (pearson correlation) for associating the microbiota and flavor compounds. Benjamini–Hochberg method was used for adjusting the P values by FDR, and the cutoff of adjusted P values was set as 0.05. The association network was displayed using R language programming^33^. O2PLS models were built using the OmicsPLS package of R^34^. Permutation test was performed to establish the thresholds for identifying the most influential variables. Datasets were reshuffled 1000 times and the O2PLS model was established for each rearranged dataset. Setting the significant level (α) of 0.05 for the two datasets, the upper and lower α/2 quantiles of the loading values were used as the latent variables thresholds^35^.

## Results

### Changes of acid, reducing sugar and alcohol

**Figure 1:**
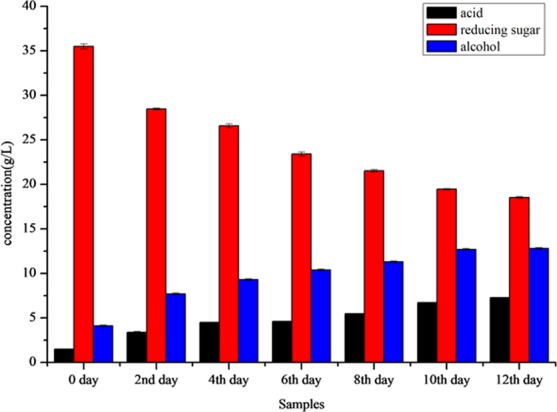
Changes of the acid, reducing sugar and alcohol found in Chinese Rice Wine brewed from *F. tataricum* during fermentation.

The data in Fig. 1 showed that the concentration of acid and alcohol increased gradually. The biggest increase was from 0 day to the 2^nd^ day, and tended to be stable at the end of fermentation. The final acid and alcohol were 7.28 ± 0.05 g/L and 12.8 ± 0.1 g/L, respectively. The content of reducing sugar rapidly reduced from 35.49 ± 0.29 g/L to 28.47 ± 0.09 g/L in the first two days of fermentation and slowly reduced in the late stage of fermentation. The final content of reducing sugar was 18.52 ± 0.23 g/L. Detailed data are attached to Supplementary Table S1.

### Changes of organic acids in the CRW during fermentation

The amount of organic acids directly influences the aroma and taste of CRW. The data showed that malic acid, lactic acid, citric acid and succinic acid were the dominant acids. The highest total organic acid content was on the 10^th^ day, while the lowest organic acid content was on day 0. The succinic acid content was the highest and the lactic acid content was second one among organic acids. The succinic acid content showed an upward trend overall and rose sharply in the first two days, then slowly rose but fluctuated slightly, and finally reached 2753.95 ± 4.70 mg/L. The lactic acid content showed an upward trend in 0–8 days, and it rose sharply in the first two days, then slowly rose and reached 1776.59 ± 5.04 mg/L. But there was a slight downward trend on 8–12 days. Malic acid content rose to 87.60 ± 6.41 mg/L in the first two days. But after that it continuously reduced to 17.93 ± 0.22 mg/L. The citric acid content decreased sharply on the 2^nd^ day and then was steady. The tartaric acid content keep a continuous fluctuation throughout the fermentation. The oxalic acid, pyruvic acid and α-ketoglutaric acid contents had a similarly growing trend that was a slight increase in the first two days and then trended to steady. Detailed data are attached to Supplementary Table S2.

### Changes of volatile compounds in the CRW during fermentation

A total of 107 volatile compounds were detected in the 21 samples at seven time points, including 19 alkanes, 21 alcohols, 31 esters, 7 ketones, 11 pyrazines, 5 acids, 4 phenols, and 9 other compounds (see Supplementary Table S3). The number of esters tended to increase from the 2^nd^ day to a peak on the 6^th^ day, which was 19. The number of esters were always higher than other volatile compounds, and the number of alcohols were the second highest. The kinds of alkanes reached maximum on the 6^th^ day, which was 7, and declined slightly at the 8^th^ day. The number of ketones, pyrazines, phenols, acids and other volatile compounds remained relatively stable with fluctuations in the fermentation process. On the last day, 37 compounds were detected, including 2 alkanes, 10 alcohols, 14 esters, 2 ketone, 3 pyrazines, 2 acids, 3 phenols, and 1 other compound. Among the esters, the relative concentrations of hexanoic acid ethyl ester, octanoic acid ethyl ester, decanoic acid ethyl ester, (E)-9-octadecenoic acid ethyl ester, 9,12-octadecadienoic acid ethyl ester, acetic acid phenylethyl ester and hexadecanoic acid ethyl ester were much higher than other esters. Ethyl alcohol can react with the acetyltransferase catalyst via esterification in the fermentation of CRW. Among the alcohols, 2-methyl-1-propanol, 3-methyl-1-butanol, 1-hexanol, 2,3-butanediol and phenylethyl alcohol, which existed in each period of fermentation in this study, were the main alcohols, which is in agreement with the previous report^36^. Volatile phenols also had a high concentration during CRW fermentation using *F*. *tataricum* grain as the feedstock. Among the phenols, 2-methoxy-4-vinylphenol was the main one, which had two maxima at the 4^th^ and 12^th^ days. Additionally, there were 11 pyrazine compounds identified in CRW samples.

### Changes in bacterial communities during different fermentation stages

To detect the intra- and inter variabilities in the bacterial community among the seven groups, we estimated a series of alpha diversity indices, including the Shannon and Simpson indices (see Supplementary Fig. S1). As shown in the Fig. S1a, the bacterial community diversity was highest on the 8^th^ day. The changing trend in the diversity increased gradually and then decreased. The Simpson diversity index showed that the highest community diversity was on the 12^th^ day (see Fig. S1b).

**Figure 2:**
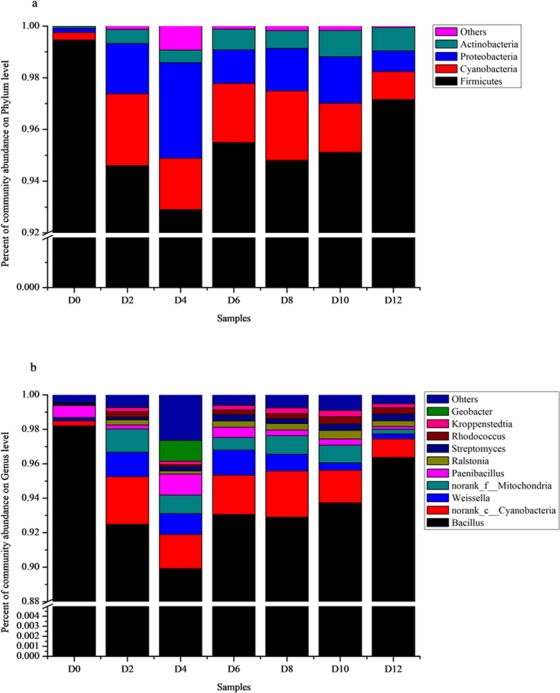
Bacterial community distribution from D0 to D12. Relative abundance of the most abundant bacteria at the phylum (**a**) and genus (**b**) levels were analyzed. D0 stands for the day 0 sample and the remainder are deduced by analogy. The members of the other populations were placed in an artificial group designated as “Others”.

Sequencing of the bacterial communities acquired a total of 1,085,965 sequences, which passed the quality control filters. The 16S rRNA gene sequences indicated that 168 bacterial genera can be found in the fermentation broth and were distributed among 17 phyla and 108 families. Most microorganisms in the fermentation broth belonged to three bacterial phyla (*Firmicutes*, *Proteobacteria* and *Actinobacteria*) (see Fig. 2a). The phylum *Firmicutes* was dominant. At the genus level, the dominating genera was *Bacillus* (see Fig. 2b). *Weissella* and *Paenibacillus* have a higher abundance than the other bacteria. *Bacillus* decreased gradually from the 0 to 4^th^ day and then slightly increased from the 6^th^ day to the end of fermentation. *Weissella* increased and appeared at its maximum on the 8^th^ day and then decreased. On the 8^th^ day, most of the top 20 genera were the most abundant, which indicated that the fermentation reached the highest level on the 8^th^ day. Conversely, most of the top 20 genera had the lowest abundance on the last day, except for *Bacillus*. Among the bacterial communities, *Bacillus* was the dominant bacteria, ranging from 90.05 to 98.21%. Compared with *Bacillus*, *Lactobacillus*, *Lactococcus* and *Weissella* had a different tendency. All of them are lactic acid bacteria (LAB). These groups of bacteria decreased sharply at the beginning of fermentation, increased on the 4^th^ day and eventually decreased slightly at the end of fermentation. In this study, the relative abundance of *Lactobacillus* was lower, while *Weissella* was higher. Therefore, *Weissella* was probably the main genus that generated acids and could inhibit the growth of other bacteria. *Saccharopolyspora* belonged to *Actinomycetales*, which peaked after 10 days. Detailed information on the composition of bacteria is shown in the Supplementary Table S4.

**Figure 3:**
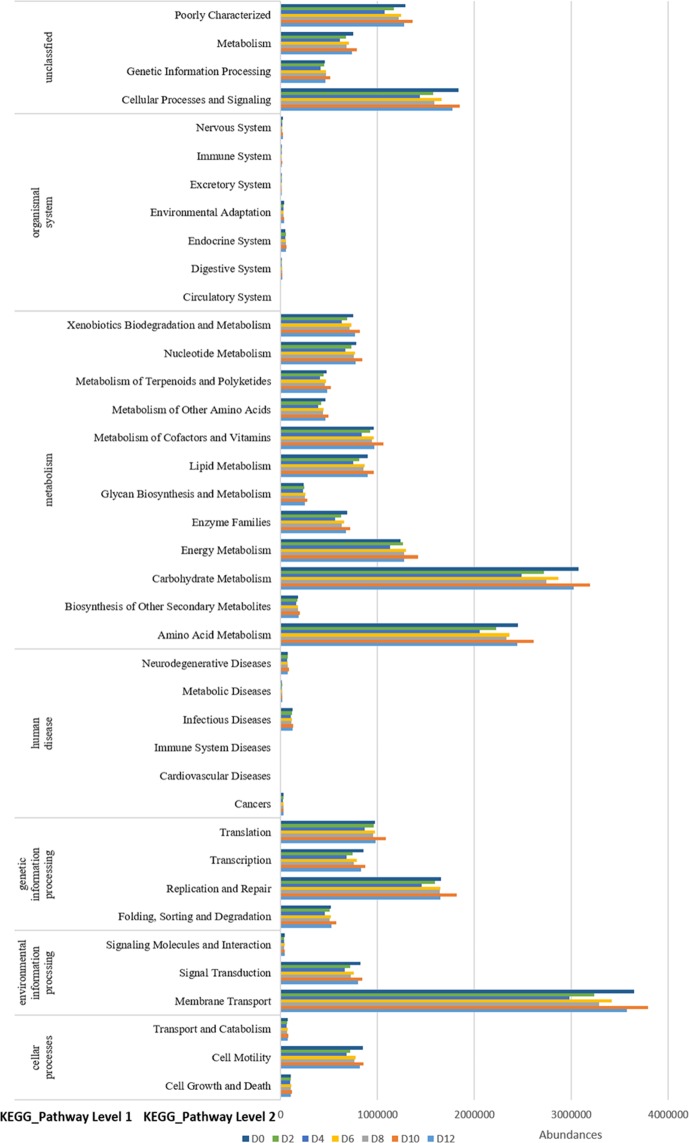
Relative abundance levels of the predicted gene function related to the KEGG pathways at levels 1 and 2.

Based on the 16 S rRNA gene sequencing data, PICRUSt was performed to predict the function of the microorganisms in fermented mash, which showed similar gene functions among all the 21 samples, which were categorized into 39 level 2 KEGG pathways that was shown at Fig. 3 (human disease pathway has been deleted), in which Membrane Transport, Carbohydrate Metabolism,Amino Acid Metabolism, Replication and Repair,Cellular Processes and Signaling, Energy Metabolism,Poorly Characterized, Translation, Metabolism of Cofactors and Vitamins were the dominant KEGG pathways. Detailed information of the predicted gene functions related to KEGG pathways at levels 3 is shown in the Supplementary Table S5.

### Changes in fungal communities during different fermentation stages

**Figure 4:**
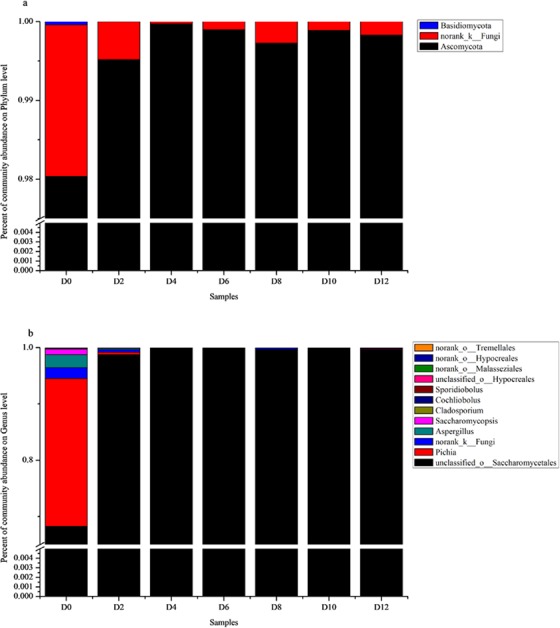
Fungal community distribution from D1 to D12. Relative abundance of the most abundant fungi at the phylum (**a**) and genus (**b**) level were analyzed. D0 stands for the day 0 sample, and the remainder can be analogously deduced. The members of the other populations were placed in an artificial group designated as “Others”.

A total of 1,029,988 sequences for the fungal communities were obtained after quality control processing. Three phyla, 12 families and 12 genera were detected. Approximately 99% of the ITS region sequences could be assigned to the Ascomycota (see Fig. 4a). At the genus level, unclassfied_O_*Saccharomycetale*s had the highest percent relative abundance in the fermentation and remained basically stable with slight fluctuation. It was the dominant fungi genus, occupying more than 70% abundance during fermentation. (See Fig. 4b). The relative abundance of Pichia peaked at day 0 and then decreased rapidly. There were fewer kinds of fungal genus participating in the fermentation of CRW. Detailed information of fungal is shown in the Supplementary Table S6.

### Association between microorganisms and flavor compounds

**Figure 5:**
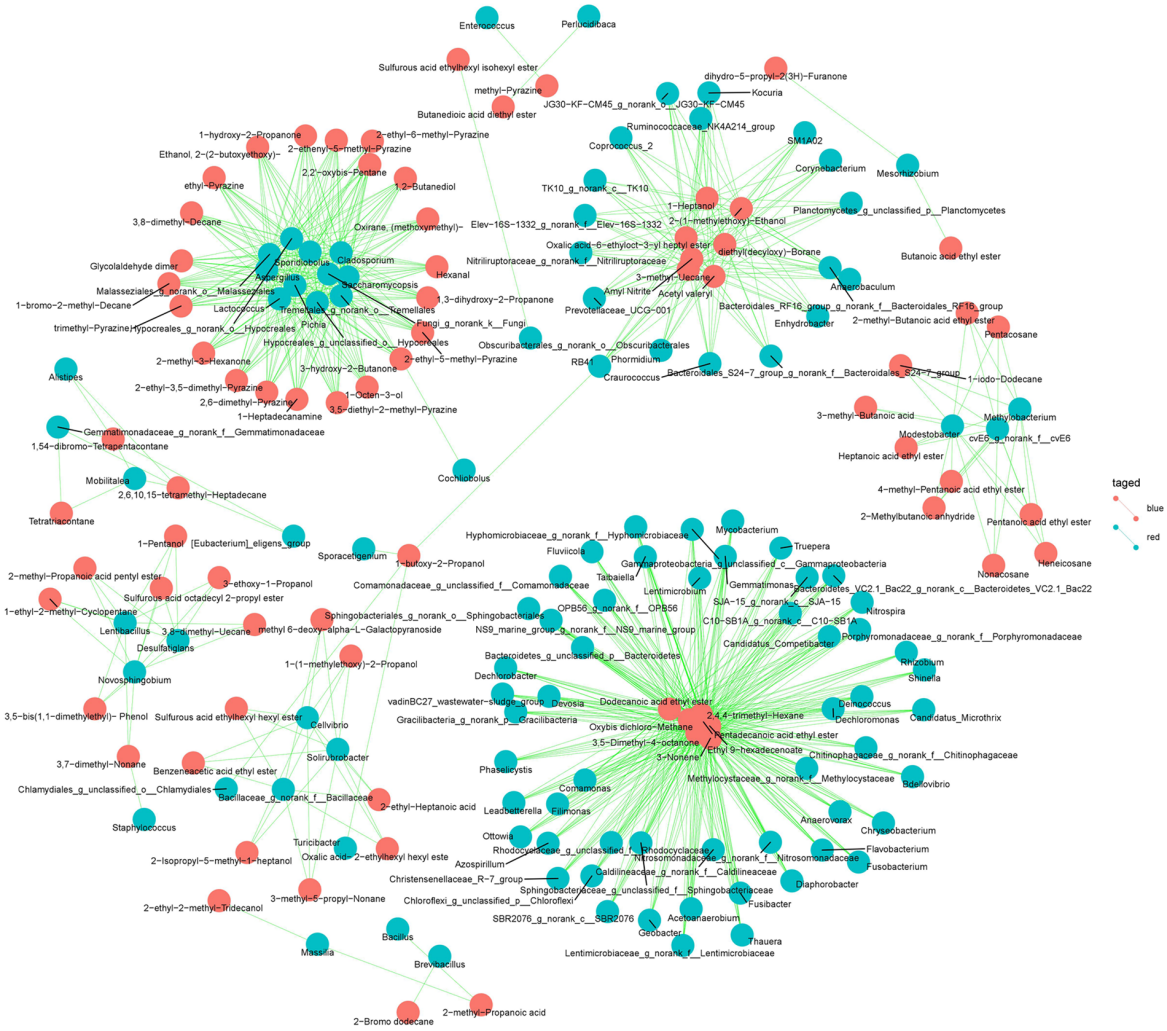
Correlation between the microorganisms and flavor compounds in *F. tataricum* grain.

A correlation analysis of the microbial and flavor substances during *F*. *tataricum* grain fermentation was conducted. To obtain a closer correlation, the P value was adjusted. By filtering the data with a P adjust less than 0.05, we established 838 correlations (see Fig. 5 and Supplementary Table S7). A total of 108 microbial genera may be involved in the formation of 72 flavor compounds (adjusted P < 0.05). Through the correlation analysis we predicted the following results. *Aspergillus*, *Cladosporium*, norank_O_*Hypocreales*, unclassified_O_*Hypocreales*, *Lactococcus*, *Saccharomycopsis*, *Sporidiobolus*, *Pichia*, norank_O_*Malasseziales et al*. participated in the formation of the largest number of flavor compounds, which were up to 22. *Bacillus*, *Staphylococcus*, *Cochliobolus*, *Perlucidibaca*, *Enterococcus* and norank_O_*Obscuribacterales* were only related to 2-methyl-propanoic acid, 3,7-dimethyl-nonane, 3-hydroxy-2-butanone, butanedioic acid diethyl ester, methyl-pyrazine and sulfurous acid ethylhexyl isohexyl ester, respectively.

Pyrazines as the special flavor compounds that were rarely detected (include 2,6-dimethyl-pyrazine, 2-ethenyl-5-methyl-pyrazine, 2-ethyl-3,5-dimethyl-pyrazine, 2-ethyl-5-methyl-pyrazine, 2-ethyl-6-methyl-pyrazine, 3,5-diethyl-2-methyl-pyrazine, ethyl-pyrazine and trimethyl-pyrazine) were correlated with *Aspergillus*, *Cladosporium, Cladosporium*, norank_K_*Fungi*, *Lactococcus*, *Pichia*, norank_O_*Hypocreales*, unclassified_O_*Hypocreales*, norank_O_*Malasseziales*, *Saccharomycopsis*, *Sporidiobolus* and norank_O_*Tremellales*. A total of 88 correlations were establish between pyrazines and microorganism.

### Association between functional significant microbiota and flavor compounds

The result of analysis based on O2PLS model shown that 14 genera had significance. Among them, unclassified_O_*Saccharomycetales* had upper quantiles. *Lactococcus*, *Pediococcus*, *Aspergillus*, *Cladosporium*, *Cochliobolus*, *Sporidiobolus*, *Pichia*, *Saccharomycopsis*, *norank_O_Hypocreales*, *unclassified_O_Hypocreales*, *norank_K_Fungi*, *norank_O_Malasseziales* and *norank_O_Tremellales* had lower quantiles (Supplementary Table S8).

**Figure 6:**
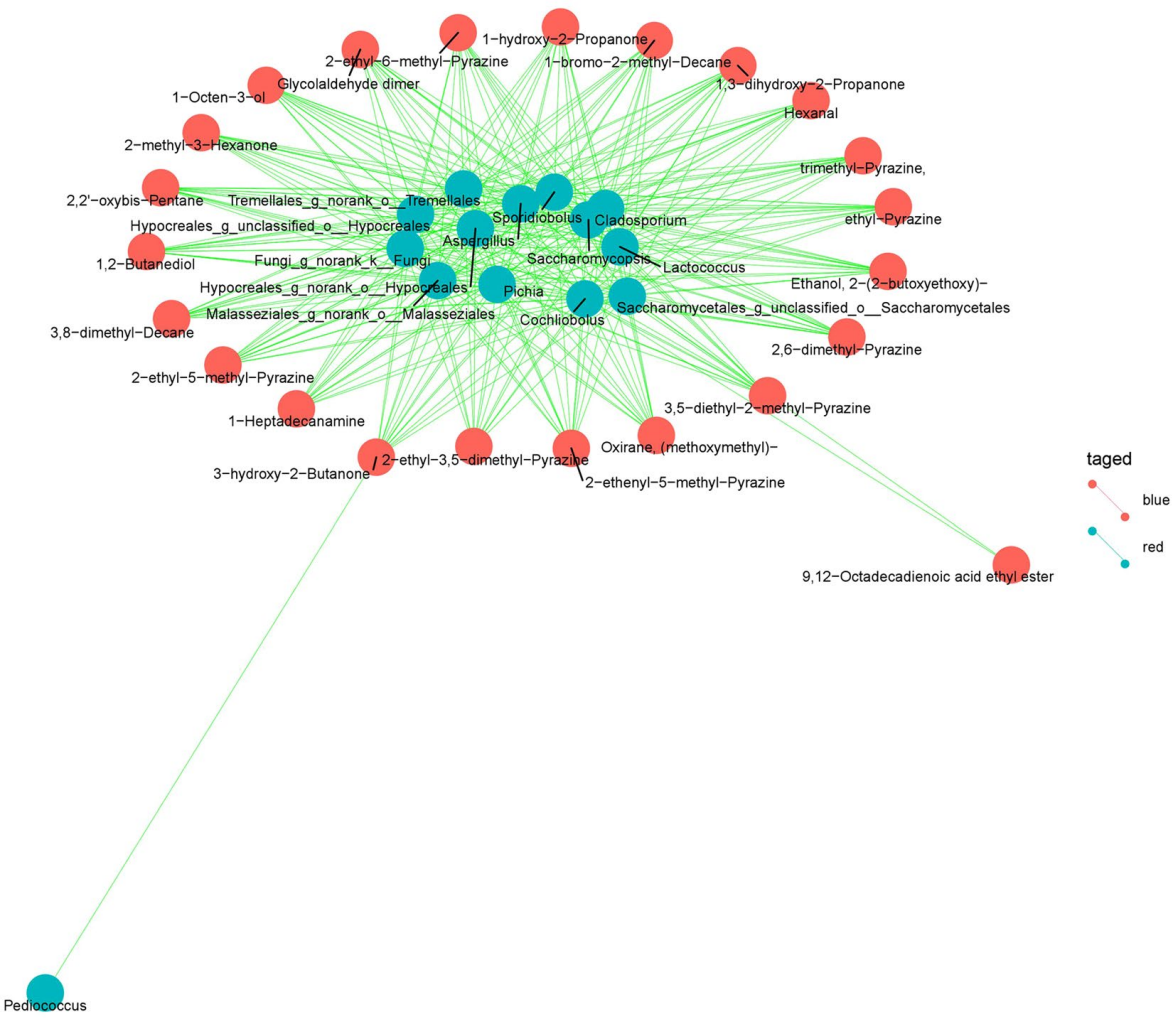
Association between functional significant microbiota and flavor compounds in *F. tataricum* grain.

Further analysis was performed to investigate the relationship of significant microbiota correlated with flavor compounds during CRW fermentation process. It was shown that these significant microbiota built association with 23 kinds of flavor compounds including 8 pyrazines, 4 ketones, 4 alkanes, 3 alcohols, 2 aldehydes, 9,12-octadecadienoic acid ethyl ester and 1-heptadecanamine. A total of 289 correlations had shown in Fig. 6 and Supplementary Table S9. *Pediococcus* only built correlation with 3-hydroxy-2-butanone. Four genera yeast (*Saccharomycopsis*, *Sporidiobolus*, unclassified_O_*Saccharomycetales* and *Pichia*) had similar correlation results, and only unclassified_O_*Saccharomycetales* additionally built correlation with 9,12-octadecadienoic acid ethyl ester. Eight kinds of pyrazines built correlations with functional significant microbiota except *Pediococcus*.

## Discussion

The metabolic activity of microorganisms involved in industrial fermentation depends on the fermentation conditions and availability of nutrients. In a natural environment or under artificial control, the microbial community is likely to produce various metabolites using different materials. This experiment adopted *F. tataricum* grain as the fermentation material, which is different from the southern CRW made from glutinous rice. Using Wheat Qu to brew wine is characteristic of CRW, and it is also a traditional operation process of the CRW fermentation process.

In the fermentation process of CRW, the change of acid, alcohol and reducing sugar are the important physical and chemical indicators which indirectly reflect the vital activities of microorganisms^37^. The results showed that ethanol was mainly produced in the first two days because a large amount of reducing sugar was utilized by the yeasts, as the reducing sugar rapidly reduced and the alcohol increased^38^. Subsequently, the metabolism of yeasts were inhabited by alcohol. As a result, the utilization of sugar by yeasts was reduces and the ability to produce alcohol weaken^39^. For acid, suitable environment promoted growth of acidogenic bacteria at first two days while the content of acid rose fast. As the fermentation proceeds, high alcohol and acid concentration restrained acid-producing microbes from growing, so that the acid content began to rise slowly.

Micro-substances such as alcohol, acid, ester and aldehyde contribute to the flavor of CRW^40^. The experimental results showed that the alcohol had the highest proportion among the volatile compounds, and the higher alcohols had the highest proportion among the alcohol compounds. Some research indicated that there were two main pathways for higher alcohol metabolism: the Ehrlich metabolic pathway in which amino acids are converted to alcohols by deamination and the anabolic pathways that by the synthetic pathway of glucose^41^. At the same time, the fermentation broth was rich in various microorganisms and enzymes. Therefore, lipid oxidation might be another way to produce alcohol compounds^42^. The Ehrlich metabolic pathway not only produced volatile compounds (higher alcohols), but also decomposes some bitter amino acids (leucine), improving the flavor of CRW^43^. In addition, the experimental results showed that changing trend of alcohol and alkane were opposite, so they might be mutually transformed.

Esters are the most important flavor components, which contribute fruit and floral aroma^44^. One source of esters is the esterification of fatty acids and alcohols. Another source is the anabolism of microorganisms by the use of higher alcohols under the action of acetyltransferases^45^. The results showed that ethyl ester was the most abundant ester, indicating that ethyl esters may play an important role in the composition of volatile components. Phenols, pyrazines had the similar trend with esters. Among them, 2-methoxy-4-vinylphenol was the most important phenolic, which can contribute aroma and had a low threshold (3 µg/L)^46^. It was reported that the content of 2-methoxy-4-vinylphenol in Wheat Qu was high^47^. It might be derived from Wheat Qu^48^, but whether it was related to the metabolism of microorganisms in CRW has not been reported. Pyrazines are important flavor compounds in many fermented foods and alcoholic beverages, with baking, nuts and cocoa aroma. It is a heterocyclic nitrogen compound that can be used as a pharmaceutical, as well as in flavorings and spice intermediates.

Ketones had a higher content than other flavor compounds in the beginning of fermentation. It indicated that feedstock such as Wheat Qu might contain ketones which also participated in the metabolism of microorganisms in fermentation^49^.

The initial microbial community participating in fermentation were mainly brought in by the Wheat Qu, however, the process of making Wheat Qu was completely open, as a result, the kinds of microorganisms contained in the Wheat Qu were extremely complicated. The changes in the composition of the bacterial communities affected the quality of CRW, and thereby reflected its role in the CRW fermentation process. At the phylum level, the bacterial communities detected in CRW fermented with *F. tataricum* were similar to those fermented with rice in Zhejiang Province, but they were obviously different at the genus level^50^. At the beginning of fermentation, most microorganisms were brought in from the feedstock, brewing water and the environment. In the first two days, as the nutrients were richer, microorganisms grew quickly in this environment. However, as the fermentation continued, alcohol and acid increased and oxygen decreased, which inhibited the microorganisms’ growth.

In this study, *Bacillus* was the dominating genus during the fermentation process. *Bacillus* which can produce spores, has stronger environmental adaptability and resistance to acid and alcohol^51^. This may be one of the reasons why it can exist with high relative abundance during fermentation. In addition, *Weissella* also has a higher abundance, and is the main genus of acid production by which inhibit the growth of other microorganisms. There is similarity in the fungal diversity of CRW that fermented by *F.tataricum* and rice. According to previous reports of Shaoxing CRW, Ascomycota occupied 99% of the total fungal population at the phylum level. In this study, the data showed the same result. At the genus level, there were fewer fungal communities participating in the fermentation of CRW. Unclassfied_O_*Saccharomycetales* and *Pichia* as major fungal genus may produce more volatile compounds or nutrients.

This experiment used an association analysis to speculate that *Cochliobolus* and unclassified_O_*Saccharomycetales* were closely related to the formation of 9,12-octadecadienoic acid ethyl ester. In addition, *Cellvibrio* was associated with benzeneacetic acid ethyl ester, oxalic acid- 2-ethylhexyl hexyl ester and sulfurous acid ethylhexyl hexyl ester. *Bdellovibrio* functions as a phage, which can stably decompose protein and use peptide and amino acids as a carbon source and energy source, which was related to the formation of pentadecanoic acid ethyl ester. This may be related to the ability of *Bdellovibrio* to produce various enzymes depending on the host cell. *Rhizobium* is a heterotrophic bacterium that uses carbohydrate various salts and organic acids (mainly malic and succinic acid) as carbon sources and can utilize mannitol and other sugars to produce acids. During fermentation, it was related to the formation of pentadecanoic acid ethyl ester and ethyl 9-hexadecenoate.

Acetoin (3-hydroxy-2-butanone) is an important physiological metabolite excreted by many microorganisms, serves as a quality index of fermented products and is an important flavor in vinegar^52^. Members of *Acetobacter*, *Lactococcus*, *Klebsiella*, *Enterobacter*, and *Bacillus* are considered acetoin biosynthesis bacteria in various fermentation processes^53,54^. In our study, speculated the synthesis of acetoin was closely related to *Aspergillus*, *Cladosporium*, *Cochliobolus*, *Lactococcus*, *Pichia*, *Pediococcus*, norank_O_*Malasseziales*, *Saccharomycopsis*, *Sporidiobolus* and unclassified_O_*Saccharomycetales*. Biosynthesis of acetoin is a complex process.

Pyrazine are rarely detected in southern CRW. It is produced mostly by non-enzymatic browning in the Maillard reaction^55^. The occurrence of microbial metabolites of pyrazine compounds is rare, but not absent. A previous study showed that *Bacillus pumilus* is a high-yield bacterial strain for tetramethyl pyrazine biosynthesis^56^. In this experiment, all the pyrazine built correlation with the same microbial genera.

About acids, most *Lactobacillus* species had the ability to produce lactic acid, ethanol and acetic acid. The genera *Pichia*, *Saccharomyces*, and *Zygosaccharomyces* can convert lactic acid to pyruvate. Moreover, they converted pyruvate to acetyl-CoA, acetaldehyde in liquor production^57^. The citric acid-accumulating microorganisms mainly include several yeast strains and molds from the genera *Aspergillus*, *Penicillium*, and *Candida*^58^. The malic acid-producing microorganisms were also mainly from *Saccharomyces* and *Aspergillus*^59,60^. Except for the reported microbe communities, the possibly main microorganisms associated with organic acids in this experiment were *Saccharomycopsis* (which ferments one or more sugars, including galactose, glucose, maltose and sucrose, producing amino acids, organic acids, inorganic acids, etc.), *Lactococcus* (which uses some carbohydrates to ferment acid; L(+)-lactic acid is the main product), *Pediococcus* (which produces acids by the fermentation of sugars; the main product is DL, or L(+)-lactate. Some strains can produce diacetyl butter or a cream aroma), *Streptomyces* (which can utilize glucose and have a strong starch and protein hydrolysis ability. It is the most important antibiotic-producing bacteria, and some strains can produce industrial protease, glucose isomerase, vitamin B12 and others), *Bacillus* (which has strong protease, amylase and lipase activity and can synthesize many organic acids, enzymes, physiological activities and other substances), *Acetobacter* (which can oxidize ethanol to produce acetic acid and can be further oxidized into carbon dioxide, which can cause a variety of low alcohol wine deterioration and can be used in brewing vinegar).

In the high expression metabolic pathway, ABC transporters are members of a transport system superfamily that is one of the largest and is possibly one of the oldest families, with representatives in all the extant phyla from prokaryotes to humans^61^. ABC transporters take up a large variety of nutrients, biosynthetic precursors, trace metals and vitamins, while exporters transport lipids, sterols, drugs, and a large variety of primary and secondary metabolites^62^. Glycolysis/Gluconeogenesis is a series of reactions that degrade glucose and glycogen to pyruvate and ATP. It is a ubiquitous pathway of glucose degradation in all living organisms. Most monosaccharides, such as sugars and galactose, can be converted into one of these intermediates. The intermediates can be used directly. For example, the intermediate product, dihydroxy phosphate acetone (DHAP), is a combination that forms the source of fatty glycerol fatty acids. Metatranscriptomic analysis revealed pyruvate metabolism in yeasts (genera *Pichia, Schizosaccharomyces*, *Saccharomyces* and *Zygosaccharomyces*) and lactic acid bacteria (genus *Lactobacillus*), which was classified into two stages in the production of the flavor components, with the genus *Schizosaccharomyces* serving as the core functional microorganism and the genus *Lactobacillus* serving as the core functional microorganism^57^. Specific expression of genes in metabolism also requires subsequent meta-analysis.

In this experiment, the micro-ecological structure of *F. tataricum* grain wine was detected by high throughput sequencing, and HS-SPME/GC-MS and HPLC were used to determine the kinds and contents of the flavor substances. The correlation analysis predicted the relationship between the core of the flavor substances and the microorganisms. In addition to the known major microbial communities, there are many new microorganisms involved in the production of the core flavor compounds. Fermenting rice wine with *F. tataricum* can not only increase the variety of CRW, but also increase the added value of *F. tataricum*. How to improve the flavor and quality of CRW is important to every producer. To produce high-quality CRW, we must make full use of the aroma producing microorganisms. So the analysis of flavor substances and microorganisms’ relationship maybe provide some hints. According to our knowledge, this is the first report to analyse the relationship between the structure and function of microbial community in *F. tataricum* fermentation.

## Supplementary information

supplementary information

## Data Availability

All data generated or analyzed during this study are included in this published article (and its Supplementary Information files). Sequence data at https://www.ncbi.nlm.nih.gov/bioproject/ Accession: PRJNA483531.

## References

[1] Chen S, Xu Y (2012). The influence of yeast strains on the volatile flavour compounds of Chinese rice wine. Journal of the Institute of Brewing.

[2] Wang JG, Shen YG, Lu WJ, Qian YL (2012). Situation and development trend of Chinese rice wine research. China Brewing.

[3] Wang, J. G. Function of traditional koji in rice wine and its characteristics. *China Brewing* (2004).

[4] Wang PX (2014). Changes in flavour characteristics and bacterial diversity during the traditional fermentation of Chinese rice wines from Shaoxing region. Food Control.

[5] Li H (2016). Process of buckwheat rice wine technology. Food Research and Development.

[6] Wan P, Yang Y, Yang L, Yang W, Zhao G (2015). Optimization of main fermentation technology for buckwheat dry rice wine by response durface method. Journal of Food Science and Biotechnology.

[7] Bonafaccia G, Marocchini M, Kreft I (2003). Composition and technological properties of the, flour and bran from common and tartary buckwheat. Food Chemistry.

[8] B, J., Bo, E. & I, K. Studies on protein fractions and protein quality of buckwheat. *Genetika* **13**, 115–121 (1981).

[9] Li, X. L., Shi, X.H. & Zhu, H. J. Present situation of development and application of tartary buckwheat products and its development strategy. *Journal of Shanxi Agricultural Sciences* (2011).

[10] Yu L (2016). Maopu Tartary buckwheat wine: The big winner of health liquor. New food.

[11] Cao Y, Xie GF, Wu C (2012). A study on characteristic flavor compounds in traditional Chinese rice Wine -Guyue Longshan rice wine. Journal of the Institute of Brewing.

[12] Xu E, Long J, Wu Z, Li H, Wang F (2015). Characterization of volatile flavor compounds in Chinese rice wine fermented from enzymatic extruded rice. Journal of Food Science.

[13] Lv XC, Huang RL, Chen F, Zhang W, Rao PF (2013). Bacterial community dynamics during the traditional brewing of Wuyi Hong Qu glutinous rice wine as determined by culture-independent methods. Food Control.

[14] Hu, Z., Xie, G., Wu, C., Cao, Y. & Lu, J. Research on prokaryotic microbes in mash during yellow rice wine big pot fermentation. Liquor-Making Science & Technology (2009).

[15] Xie G, Wang L, Gao Q, Yu W, Hong X (2013). Microbial community structure in fermentation process of Shaoxing rice wine by Illumina-based metagenomic. Journal of the Science of Food & Agriculture.

[16] Liu M, Tang Q, Xu Z, Xu Z (2016). Bacterial ommunity diversity of jannanchun Koji based on metagenomics. Liquor Making.

[17] Randazzo C, Heilig H, Restuccia C, Giudici P, Caggia C (2005). Bacterial population in traditional sourdough evaluated by molecular methods. Journal of Applied Microbiology..

[18] Ji Y, Lee S, Kim J, Park M, Bae J (2011). Metagenomic analysis of kimchi, a traditional Korean fermented food. Applied & Environmental Microbiology.

[19] Wang P, Wu Q, Jiang X, Wang Z, Tang J (2017). Bacillus licheniformis, affects the microbial community and metabolic profile in the spontaneous fermentation of Daqu, starter for Chinese liquor making. International Journal of Food Microbiology..

[20] Wang, Z., Lu, Z., Shi, J. & Xu, Z. Exploring flavour-producing core microbiota in multispecies solid-state fermentation of traditional Chinese vinegar. *Scientific Reports* **6** (2016). 10.1038/srep26818 PMC4886211 27241188

[21] Kaijie Lu Z (2013). Optimization of salicylic acid colorimetric determination of reducing sugar by response surface methodology. Journal of the Chinese cereals and oils association.

[22] General Administration Of Quality Supervision, I. A. Q. O. & China, T. P. R. O. Chinese rice wine. in *GB/T13662-2008* (eds Apos, S. A. O. T. & China, S. R. O.) (2008).

[23] Tao L, Fan W, Yan X (2008). Characterization of volatile and semi-volatile compounds in Chinese rice wines by headspace solid phase microextraction followed by gas chromatography-mass spectrometry. Journal of the Institute of Brewing.

[24] Ye FR, Chen XD, Ni XH (2013). Study of analytical method for organic acids in yellow rice wines by high performance liquid chromatography. Liquor Making.

[25] Zhang XL (2015). Biodiversity of the symbiotic bacteria associated with toxic marine dinoflagellate alexandrium tamarense. Journal of Biosciences & Medicines.

[26] Buée M, Reich M, Murat C (2009). 454 Pyrosequencing analyses of forest soils reveal an unexpectedly high fungal diversity. New Phytologist.

[27] Ren, G., Ren, W. & Teng, Y. Evident bacterial community changes but only slight degradation when polluted with pyrene in a red soil. *Frontiers in Microbiology* **6** (2015). 10.3389/fmicb.2015.00022 PMC4311681 25688237

[28] Magoč T, Salzberg SL (2011). FLASH: fast length adjustment of short reads to improve genome assemblies. Bioinformatics.

[29] Caporaso J (2010). QIIME allows analysis of high-throughput community sequencing data. Nature Methods.

[30] Kemp P, Aller J (2004). Bacterial diversity in aquatic and other environments: what 16S rDNA libraries can tell us. Fems Microbiology Ecology.

[31] Chang CW, Laird DA, Mausbach MJ (2001). Near-Infrared reflectance spectroscopy–principal components regression analyses of soil properties. Soil Science Society of America Journal.

[32] Lozupone C, Lladser ME, Dan K (2011). UniFrac: an effective distance metric for microbial community comparison. Isme Journal.

[33] Xu, J. *et al*. Microbial dynamics and metabolite changes in Chinese Rice Wine fermentation from sorghum with different tannin content. *Scientific Reports* **8** (2018). 10.1038/s41598-018-23013-1 PMC5854674 29545525

[34] El Bouhaddani, S. *et al*. Evaluation of O2PLS in Omics data integration. *BMC Bioinformatics* **172** (2016). 10.1186/s12859-015-0854-z PMC4959391 26822911

[35] Rodriguez, V.M. *et al*. Maize Stem Response to Long-Term Attack by Sesamia nonagrioides. *Frontiers in Plant Science* **9** (2018). 10.3389/fpls.2018.00522 PMC5925969 29740463

[36] Luo T, Fan W, Xu Y (2008). Characterization of volatile and semi-volatile compounds in Chinese rice wines by headspace solid phase microextraction followed by gas. Journal of the Institute of Brewing.

[37] Yun, L.Y. Characterization of microflora and their functions on flavor compounds in Shaoxing rice wine. (Jiangnan University. Jiangsu, Wuxi, 2015).

[38] Li Y (2009). Discussion on rice wine acidity and its related indexes. China Brewing.

[39] Zhang M (2012). Research progress in mechanism of alcohol tolerance of sake yeast. Science and Technology of Food Industry.

[40] Li J (2000). Sources of color components, aroma components and taste components in yellow rice wine. Liquor-Making Science & Technology.

[41] Wondra M, Berovič M (2001). Analyses of aroma components of chardonnay wine fermented by different yeast strains. Food Technology and Biotechnology.

[42] Cramer ACJ, Mattinson DS, Fellman JK (2005). Analysis of volatile compounds from various types of barley cultivars. Journal of Agricultural and Food Chemistry.

[43] Shou HZ, Ling ZY, Yang X, Xie GF (2007). Relationship between metabolites of wheat Qu microorganisms and flavor of rice wine. China Brewing.

[44] Fan WL, Qian MC (2006). Characterization of aroma compounds of Chinese ‘Wuliangye’ and ‘Jiannanchun’ liquors by aroma extract dilution analysis. Journal of Agricultural and Food Chemistry.

[45] Mo XL, Fan WL, Xu Y (2009). Changes in volatile compounds of Chinese rice wine wheat Qu during fermentation and storage. Journal of the Institute of Brewing.

[46] Buttery RG, Turnbaugh JG, Ling LC (1988). Contribution of volatiles to rice aroma. Journal of Agricultural and Food Chemistry.

[47] Mo XL, Xu Y, Fan WL (2010). Characterization of aroma compounds in Chinese rice wine Qu by solvent-assisted flavor evaporation and headspace solid-phase microextraction. Journal of Agricultural and Food Chemistry.

[48] Martinez AT, Camarero S, Gutierrez A (2001). Studies on wheat lignin degradation by Pleurotus species using analytical pyrolysis. Journal of Analytical and Applied Pyrolysis.

[49] Liu X (2015). Latest advances of microbial production of platform chemical acetoin. China Biotechnology.

[50] Liu SP, Mao J, Liu YY (2015). Bacterial succession and the dynamics of volatile compounds during the fermentation of Chinese rice wine from Shaoxing region. World J Microbiol Biotechnol.

[51] Liu, G. H., Liu, B., Lin, N. Q. & Lin, Y. Z. Phyletic evolution and taxonomic characteristics of Bacillus. *Fujian Journal of Agricultural Sciences*, 436–499 (2008).

[52] Xiao Z, Xu P (2007). Acetoin metabolism in bacteria. Critical Reviews in Microbiology.

[53] Chen JC, Chen QH, Qin G (2010). Simultaneous determination of acetoin and tetramethylpyrazine in traditional vinegars by HPLC method. Food Chemistry.

[54] Faveri DD, Torre P, Molinari F (2003). Carbon material balances and bioenergetics of 2,3-butanediol bio-oxidation by Acetobacter hansenii. Enzyme & Microbial Technology.

[55] Yu, X., Yin, J. & Hu, G. Analysis and study of nitrogen compounds in liquor. *Liquor Making*, 71–76 (1992).

[56] Xu, P., Xiao, Z. & Wei, Z. Bacillus pumilus strain for high yield of tetramethylpyrazine (2011).

[57] Song, Z., Du, H. & Zhang, Y. Unraveling core functional microbiota in traditional solid-state fermentation by high-throughput amplicons and metatranscriptomics sequencing. *Frontiers in Microbiology* **8** (2017). 10.3389/fmicb.2017.01294 PMC5509801 28769888

[58] Papagianni M (2007). Advances in citric acid fermentation by Aspergillus niger: Biochemical aspects, membrane transport and modeling. Biotechnology Advances.

[59] Peleg Y, Stieglitz B, Goldberg I (1989). Malic acid accumulation by Aspergillus flavus. Applied Microbiology & Biotechnology.

[60] Pines O, Even-Ram S, Elnathan N (1996). The cytosolic pathway of l -malic acid synthesis in Saccharomyces cerevisiae: the role of fumarase. Applied Microbiology & Biotechnology.

[61] Ponte-Sucre, A. *ABC transporters in microorganisms: research, innovation and value as targets against drug resistance*, (Caister Academic, 2012).

[62] Ter BJ, Guskov A, Slotboom DJ (2014). Structural diversity of ABC transporters. Journal of General Physiology.

