# Supplementary material for: The changes of microbial community and flavor compound in the fermentation process of Chinese rice wine using Fagopyrum tataricum grain as feedstock
Source: Sci Rep. 2019 Mar 4;9:3365. doi: 10.1038/s41598-019-40337-8 (PMC6399331; doi:10.1038/s41598-019-40337-8)
Supplement: Supplementary file 1 — supplementary information [file 41598_2019_40337_MOESM1_ESM.pdf]

The changes of microbial community and flavor compound in the fermentation process of Chinese rice wine using *Fagopyrum tataricum* grain as feedstock

Qing Ren, Leping Sun, Huijun Wu, Yousheng Wang, Zhiwei Wang, Fuping Zheng, Xin Lu, Jialiang Xu

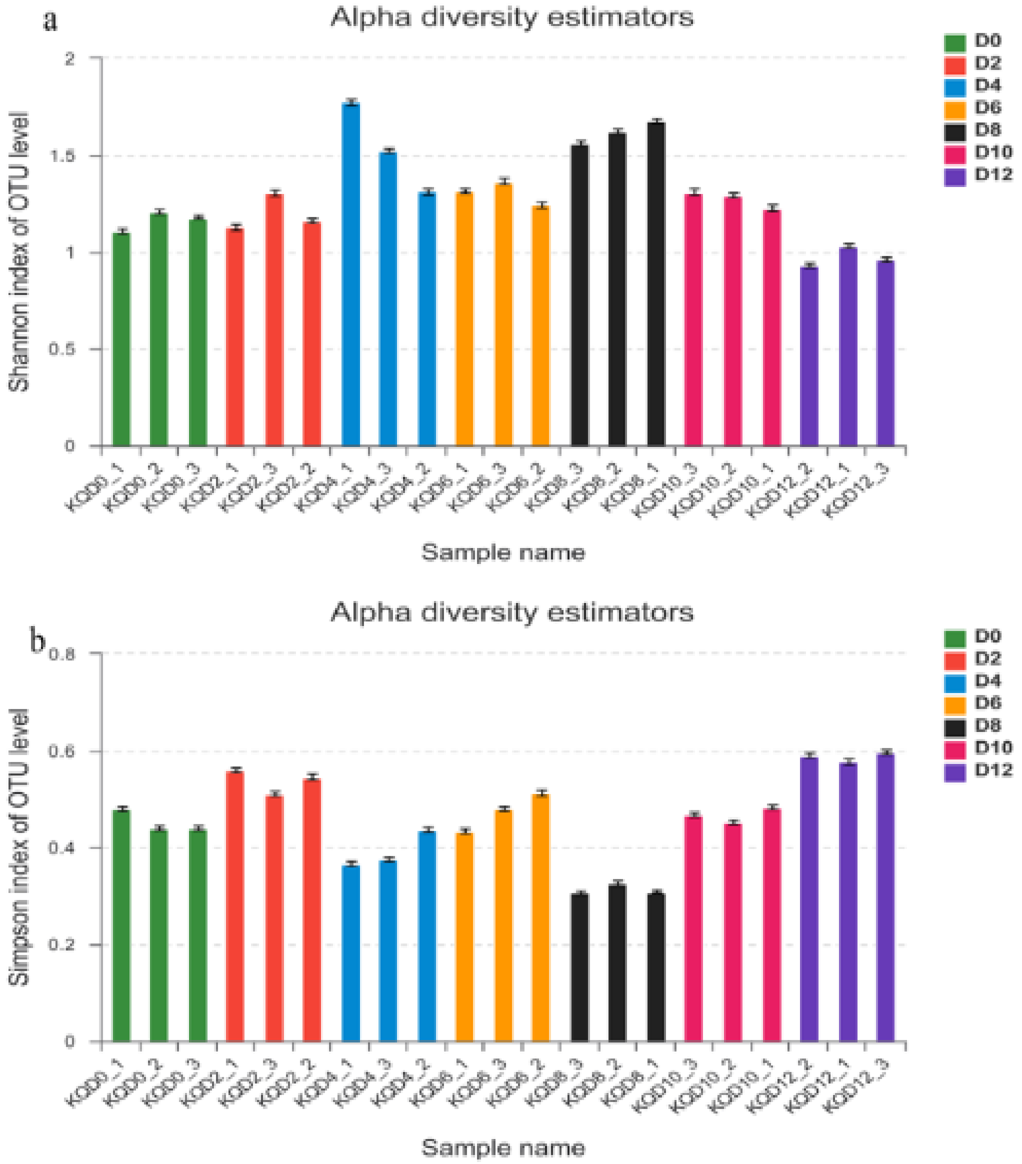

Figure S1. Distributions of alpha diversity indices (a: Shannon diversity index, b: Simpson diversity index) D0 stands for the sample of 0 day, the rest are deduced by analogy.

Table.S1 Changes in acid, reducing sugar and alcohol found in *F. tataricum* grain CRW during fermentation.

Compounds	0 day	2nd day	4th day	6th day	8th day	10th day	12th day
acid	1.49±0.05	3.40±0.07	4.49±0.05	4.60±0.05	5.47±0.03	6.71±0.03	7.28±0.05
reducing sugar	35.49±0.29	28.47±0.09	26.57±0.23	23.42±0.23	21.53±0.15	19.46±0.08	18.52±0.23
alcohol	4.1±0.1	7.7±0.1	9.3±0.1	10.4±0.1	11.3±0.1	12.7±0.1	12.8±0.1

*unite was g/L. Every value is expressed as means standard error (n=3).

Table.S2 Changes in organic acids found in *F. tataricum* grain CRW during fermentation.

Organic acid	0 day	2nd day	4th day	6th day	8th day	10th day	12th day
oxalic acid	1.03±0	10.61±0.32	10.11±0.02	13.63±0.04	15.27±0.02	15.21±0.07	13.89±0.02
tartaric acid	8.55±0.98	18.76±0.33	26.49±0.45	14.09±0.77	21.90±4.61	17.10±0.05	18.83±0.20
pyruvic acid	1.11±0.23	3.18±0.52	1.83±0.15	1.78±0.22	1.81±0.24	1.72±0.23	1.28±0.17
malic acid	68.10±7.98	87.60±6.41	48.29±1.88	46.59±1.71	38.41±6.19	38.76±11.83	17.93±0.22
α-ketoglutaric acid	2.16±0.12	3.96±0.12	3.64±0.23	2.69±0.05	3.32±0.08	3.17±0.17	2.73±0.09
lactic acid	191.34±23.97	1674.25±13.26	1853.01±3.01	2103.54±12.43	2337.91±7.73	2155.12±8.60	1776.59±5.04
citric acid	69.63±2.37	19.45±0.26	21.48±0.11	24.63±0.20	21.08±0.40	20.49±2.96	5.72±0.19
succinic acid	330.48±31.35	1708.02±42.68	1651.73±2.81	2204.47±5.78	2249.83±0.40	2680.13±9.94	2753.95±4.70

*unite was mg/L. Every value is expressed as means standard error (n=3).

Table.S3 Relative content of volatile components in different periods of *F. tataricum* grain Chinese Rice Wine during fermentation.

Compounds	Day 0	Day 2	Day 4	Day 6	Day 8	Day 10	Day12
alkane							
Heneicosane	0.25±0.12	ND	ND	11.74±1.34	ND	ND	ND
Pentacosane	0.90±0.24	ND	15.92±0.55	231.14±17.48	7.67±1.07	ND	ND
Nonacosane	ND	ND	ND	4.32±0.68	ND	ND	ND
Tetratriacontane	ND	ND	ND	5.52±0.53	15.77±1.79	ND	ND
2,4,4-trimethyl-Hexane	ND	ND	0.42±0.15	ND	ND	ND	ND
2,6,10,15-tetramethyl-Heptadecane	0.03±0.01	ND	ND	ND	0.34±0.04	ND	ND
3,7-dimethyl-Nonane	ND	1.78±0.14	0.43±0.02	0.07±0.01	ND	0.41±0.02	ND
3,8-dimethyl-Decane	0.02±0.01	ND	ND	ND	ND	ND	ND
3-methyl-5-propyl-Nonane	ND	ND	ND	ND	ND	ND	0.36±0.10
3-methyl-Undecane	ND	ND	ND	ND	ND	1.42±0.15	ND
3,8-dimethyl-Undecane	ND	0.26±0.02	ND	ND	ND	ND	ND

1-ethyl-2-methyl-Cyclopentane	ND	0.36±0.06	ND	ND	ND	ND	ND
1-bromo-2-methyl-Decane	0.01±0.01	ND	ND	ND	ND	ND	ND
2-Bromo dodecane	ND	ND	ND	0.34±0.04	ND	ND	0.42±0.11
1,54-dibromo-Tetrapentacontane	ND	ND	ND	ND	40.70±2.46	ND	ND
1-iodo-Dodecane	ND	ND	ND	0.93±0.13	ND	ND	ND
2,2'-oxybis-Pentane	0.02±0.01	ND	ND	ND	ND	ND	ND
diethyl(decyloxy)-Borane	ND	ND	ND	ND	ND	0.28±0.04	ND
Oxybis dichloro-Methane	ND	ND	0.27±0.03	ND	ND	ND	ND
alcohol							
1-Propanol	ND	9.35±1.26	4.34±0.63	1.27±0.42	4.02±0.95	7.53±1.47	4.60±0.99
1-Butanol	ND	0.34±0.06	0.26±0.09		0.34±0.03	0.50±0.07	0.32±0.03
2-methyl-1-Propanol	0.02±0.01	16.93±2.41	10.87±1.57	3.35±0.96	11.51±1.48	15.87±2.51	12.76±1.39
1-Pentanol	0.01±0.01	0.16±0.01	ND	ND	ND	ND	ND
3-methyl-1-Butanol	0.16±0.02	85.92±4.55	70.64±5.83	23.50±2.59	71.70±6.25	102.07±24.13	83.77±12.98
1-Hexanol	0.04±0.01	0.65±0.11	0.68±0.15	0.25±0.02	0.55±0.01	1.00±0.21	1.05±0.17
1-Heptanol	ND	ND	ND	ND	ND	0.61±0.03	ND
2-Isopropyl-5-methyl-1-heptanol	ND	ND	ND	ND	ND	ND	0.21±0.13
2-ethyl-2-methyl-Tridecanol	ND	2.60±0.69	3.71±1.27	ND	ND	ND	ND
3-ethoxy-1-Propanol	ND	0.27±0.04	ND	ND	ND	ND	ND
1-butoxy-2-Propanol	ND	0.33±0.02	ND	ND	ND	0.60±0.24	ND
2-(1-methylethoxy)-Ethanol	ND	ND	ND	ND	ND	2.39±0.73	ND
1-(1-methylethoxy)-2-Propanol	ND	ND	ND	ND	ND	ND	6.11±1.33
Propylene Glycol	ND	2.34±0.94	ND	1.04±0.68	1.94±1.02	ND	ND
Glycerin	ND	3.09±0.86	37.86±6.23	26.49±4.87	28.35±3.96	19.43±3.46	67.73±12.48
1,2-Butanediol	0.03±0.01	ND	ND	ND	ND	ND	ND
2,3-Butanediol	2.25±0.79	82.58±14.31	58.78±9.49	36.92±5.28	52.72±9.22	30.53±6.29	109.53±13.06
1-Octen-3-ol	0.01±0.01	ND	ND	ND	ND	ND	ND
Phenylethyl Alcohol	0.07±0.02	43.61±7.23	59.79±9.40	14.81±4.59	29.75±7.02	68.39±11.48	92.23±15.37
2-(2-butoxyethoxy)-Ethanol	0.02±0.01	ND	ND	ND	ND	ND	ND

3-(methylthio)-1-Propanol	ND	0.26±0.03	0.29±0.05	ND	ND	ND	ND
			ester				
Butanoic acid ethyl ester	ND	ND	0.24±0.01	0.19±0.02	ND	0.42±0.10	ND
2-methyl-Butanoic acid ethyl ester	ND	ND	ND	0.29±0.02	ND	ND	ND
3-methyl-1-Butanol acetate	ND	ND	ND	0.14±0.01	ND	ND	ND
Pentanoic acid ethyl ester	ND	ND	ND	0.19±0.01	ND	ND	ND
4-methyl-Pentanoic acid ethyl ester	ND	ND	ND	0.42±0.07	ND	ND	ND
Hexanoic acid ethyl ester	ND	1.20±0.23	1.67±0.18	4.38±0.76	1.39±0.28	1.63±0.34	1.62±0.69
Heptanoic acid ethyl ester	ND	ND	ND	0.37	ND	ND	ND
Octanoic acid ethyl ester	ND	0.88±0.14	1.67±0.79	3.61±1.35	0.79±0.18	1.04±0.32	1.69±0.96
Nonanoic acid ethyl ester	ND	0.19±0.03	0.64±0.12	0.50±0.09	ND	ND	1.26±0.23
Decanoic acid ethyl ester	ND	1.91±0.30	7.10±2.06	3.26±1.37	1.03±0.39	2.58±0.85	5.01±1.04
Undecanoic acid ethyl ester	ND	0.61±0.04	ND	0.28±0.02	0.29±0.03	0.49±0.07	ND
Pentadecanoic acid ethyl ester	ND	ND	4.13±1.03	ND	ND	ND	ND
Dodecanoic acid ethyl ester	ND	ND	2.15±0.92	0.76±0.04	ND	ND	ND
Tetradecanoic acid ethyl ester	ND	0.70±0.02	1.04±0.13	1.44±0.25	0.41±0.11	ND	ND
Hexadecanoic acid ethyl ester	ND	11.75±3.07	30.19±6.29	10.82±2.01	11.20±1.93	23.68±3.65	19.87±2.88
Ethyl 9-hexadecenoate	ND	ND	0.64±0.02	ND	ND	ND	ND
Octadecanoic acid ethyl ester	ND	0.64±0.05	0.91±0.08	0.49±0.02	0.62±0.11	1.11±0.24	ND
(E)-9-Octadecenoic acid ethyl ester	ND	8.80±1.79	15.28±2.06	5.62±1.26	ND	13.31±2.64	12.47±3.10
9,12-Octadecadienoic acid ethyl ester	ND	3.98±1.07	9.42±2.33	5.08±1.97	7.15±2.60	9.04±2.84	8.72±2.45
Benzoic acid ethyl ester	ND	0.81±0.03	1.36±0.32	ND	ND	1.12±0.18	2.19±0.73
Benzeneacetic acid ethyl ester	ND	ND	ND	ND	ND	ND	0.61±0.07

Butanedioic acid diethyl ester	ND	ND	ND	0.08±0.01	0.37±0.04	0.92±0.08	1.84±0.27
2-methyl-Propanoic acid pentyl ester	0.01±0.01	0.26±0.06	ND	ND	ND	ND	ND
3-methyl-1-Butanol acetate	ND	0.28±0.03	0.22±0.04	0.56±0.06	0.19±0.01	ND	ND
Amyl Nitrite	ND	ND	ND	ND	ND	2.41±0.48	ND
Acetic acid phenylethyl ester	ND	0.69±0.12	0.71±0.19	0.17±0.02	0.27±0.07	0.90±0.13	1.18±0.39
Oxalic acid- 2-ethylhexyl hexyl ester	ND	ND	ND	ND	ND	ND	0.29±0.03
Oxalic acid-6-ethyloct-3-yl heptyl ester	ND	ND	ND	ND	ND	0.42±0.02	ND
Sulfurous acid ethylhexyl hexyl ester	ND	ND	0.83±0.19	ND	ND	ND	2.86±0.73
Sulfurous acid ethylhexyl isohexyl ester	ND	0.52±0.05	ND	0.22±0.02	ND	ND	ND
Sulfurous acid butyl decyl ester	ND	0.80±0.03	0.82±0.02	ND	ND	ND	1.38±0.17
Sulfurous acid octadecyl 2-propyl ester	ND	0.25±0.07	ND	ND	ND	ND	ND
ketone							
2-Octanone	ND	4.06±1.04	5.25±2.10	7.32±2.39	3.49±0.97	6.87±1.79	8.74±2.38
2-methyl-3-Hexanone	0.02±0.01	ND	ND	ND	ND	ND	ND
3,5-Dimethyl-4-octanone	ND	ND	0.32±0.02	ND	ND	ND	ND
1-hydroxy-2-Propanone	0.01±0.01	ND	ND	ND	ND	ND	ND
3-hydroxy-2-Butanone	3.10±1.45	1.18±0.50	0.63±0.28	0.20±0.06	0.29±0.03	0.27±0.08	0.34±0.07
1,3-dihydroxy-2-Propanone	0.02±0.01	ND	ND	ND	ND	ND	ND
dihydro-5-propyl-2(3H)-Furanone	ND	ND	0.64±0.06	ND	ND	1.00±0.31	ND
			acid				
Acetic acid	0.09±0.01	6.86±2.19	4.29±1.52	9.33±2.96	13.47±3.70	ND	1.44±0.93
Hexanoic acid	ND	4.91±1.29	ND	ND	2.24±0.94	ND	ND
2-methyl-Propanoic acid	0.19±0.02	4.74±1.02	4.55±0.93	1.62±0.84	2.54±1.08	ND	ND
3-methyl-Butanoic acid	0.14±0.03	ND	ND	1.24±0.19	ND	ND	ND
2-ethyl-Heptanoic acid	ND	ND	ND	ND	ND	ND	0.40±0.04
			Pyrazine				

methyl-Pyrazine	0.04±0.01	0.45±0.07	0.37±0.03	ND	ND	0.55±0.08	0.53±0.07
ethyl-Pyrazine	0.02±0.01	ND	ND	ND	ND	ND	ND
2,5-dimethyl-Pyrazine	0.08±0.02	0.84±0.04	0.30±0.02	ND	ND	0.92±0.06	0.51±0.04
2,6-dimethyl-Pyrazine	0.03±0.01	ND	ND	ND	ND	ND	ND
trimethyl-Pyrazine,	0.05±0.02	ND	ND	ND	ND	ND	ND
2-ethyl-6-methyl-Pyrazine	0.03±0.01	ND	ND	ND	ND	ND	ND
2-ethyl-5-methyl-Pyrazine	0.04±0.01	ND	ND	ND	ND	ND	ND
3-ethyl-2,5-dimethyl-Pyrazine	0.08±0.02	0.34±0.05	0.79±0.06	ND	ND	0.55±0.05	1.33±0.20
2-ethyl-3,5-dimethyl-Pyrazine	0.03±0.01	ND	ND	ND	ND	ND	ND
3,5-diethyl-2-methyl-Pyrazine	0.03±0.01	ND	ND	ND	ND	ND	ND
2-ethenyl-5-methyl-Pyrazine	0.01±0.01	ND	ND	ND	ND	ND	ND
			phenol				
Phenol	0.21±0.04	3.40±1.39	5.59±2.07	1.64±0.85	1.43±0.36	4.53±1.79	7.05±3.18
2-methoxy-Phenol	0.19±0.01	2.44±1.08	3.11±1.42	0.73±0.11	1.17±0.49	4.48±1.96	5.33±2.06
2-Methoxy-4-vinylphenol	0.60±0.07	20.73±4.79	23.72±3.95	5.19±1.96	7.97±2.31	25.78±4.26	31.02±5.38
3,5-bis(1,1-dimethylethyl)- Phenol	ND	0.54±0.04	ND	ND	ND	ND	ND
			other				
3-Nonene	ND	ND	0.52±0.08	ND	ND	ND	ND
Oxirane, (methoxymethyl)-	0.02±0.01	ND	ND	ND	ND	ND	ND
Benzothiazole	0.21±0.04	3.92±1.03	3.55±1.21	8.62±2.79	3.34±1.37	4.85±1.27	ND
Acetyl valeryl	ND	ND	ND	ND	ND	0.28±0.02	ND
methyl 6-deoxy-alpha-L-Galactopyranoside	ND	ND	ND	ND	ND	ND	4.79±1.32
Hexanal	0.01±0.01	ND	ND	ND	ND	ND	ND
1-Heptadecanamine	0.26±0.02	ND	ND	ND	ND	ND	ND
Glycolaldehyde dimer	0.01±0.01	ND	ND	ND	ND	ND	ND
2-Methylbutanoic anhydride	ND	ND	ND	0.13±0.04	ND	ND	ND

* ND, not detected. Every value is expressed as means ± standard error (n = 3).

Table S4. Bacterial genera abundance during different fermentation stages of *F. tataricum* grain CRW.

OTU ID	KQD0	KQD2	KQD4	KQD6	KQD8	KQD10	KQD12
norank_f_0319-6G20	0	0	1	1	1	2	2
Acetoanaerobium	0	0	13	0	0	0	0
Acetobacter	7	16	4	9	3	5	1
Achromobacter	1	5	3	7	5	8	3
norank_c_Acidobacteria	0	1	0	2	0	2	2
Acinetobacter	3	13	22	7	11	10	7
norank_f_Actinopolysporaceae	0	1	2	2	4	6	5
Alistipes	0	1	0	0	6	1	1
Anaerobaculum	0	0	0	0	0	2	0
Anaerostipes	0	3	0	1	1	2	1
Anaerovorax	0	0	2	0	0	0	0
Apibacter	5	48	30	46	35	37	13
Aquabacterium	2	6	4	12	13	11	8
Azospirillum	0	0	89	0	0	3	0
norank_c_BD7-11	0	0	1	4	1	2	2
norank_f_Bacillaceae	0	0	0	0	0	0	1
Bacillus	54351	43650	40074	47678	47233	52332	51229
Bacteria_g_unclassified_k_norank	1	2	3	0	1	2	0
norank_f_Bacteroidales_RF16_group	0	0	0	0	0	2	0
norank_f_Bacteroidales_S24-7_group	0	0	0	0	0	9	0
Bacteroides	2	5	2	4	40	18	7
norank_c_Bacteroidetes_VC2.1_Bac22	0	0	46	0	0	1	0
unclassified_p_Bacteroidetes	0	0	1	0	0	0	0
Bdellovibrio	1	1	33	1	2	4	2
Bifidobacterium	0	7	2	1	7	3	5
Blautia	0	7	0	1	7	7	3
Bradyrhizobium	0	2	5	1	2	2	2
Brevibacillus	0	0	0	3	0	1	5
Brevundimonas	0	0	2	2	1	0	0
Burkholderia-Paraburkholderia	2	15	6	15	5	31	13
_norank_c_C10-SB1A	0	0	1	0	0	0	0
norank_f_Caldilineaceae	0	0	7	0	0	0	0
Candidatus_Competibacter	0	0	7	0	0	0	0
Candidatus_Microthrix	0	0	9	0	0	1	0
Carnimonas	0	2	0	2	1	2	0
Cellvibrio	0	0	0	0	0	0	5
norank_f_Chitinophagaceae	0	0	25	0	0	0	0
unclassified_f_Chitinophagaceae	0	0	0	1	0	1	1
unclassified_o_Chlamydiales	0	1	0	0	0	1	0
unclassified_p_Chloroflexi	0	0	5	0	0	0	0
Christensenellaceae_R-7_group	0	0	12	0	0	0	0

Chryseobacterium	0	0	6	0	0	0	1
Clostridium_sensu_stricto_1	0	0	1	0	2	0	2
Collinsella	0	2	1	0	3	2	1
unclassified_f_Comamonadaceae	0	0	17	0	0	0	0
Comamonas	0	0	54	0	0	0	0
Coprococcus_2	0	0	0	0	1	4	0
unclassified_f_Corynebacteriaceae	0	0	0	1	2	0	2
Corynebacterium	0	0	0	0	0	3	0
Corynebacterium_1	1	4	3	4	4	6	2
Craurococcus	0	0	0	0	0	3	0
norank_c_Cyanobacteria	162	1353	904	1200	1311	1065	582
Dechlorobacter	0	0	123	0	0	4	0
Dechloromonas	0	0	12	0	0	0	0
Deinococcus	0	0	2	0	0	0	0
Desulfatiglans	0	1	0	0	0	0	0
Devosia	0	0	8	0	0	0	0
Dialister	0	0	0	0	3	4	0
Diaphorobacter	0	0	15	0	1	0	0
norank_f_Elev-16S-1332	0	0	0	0	0	1	0
Enhydrobacter	0	1	0	0	1	3	1
Enterobacter	5	33	6	21	21	23	19
unclassified_f_Enterobacteriaceae	1	2	2	4	3	1	7
Enterococcus	0	2	2	0	0	2	1
Escherichia-Shigella	1	3	3	9	9	11	10
Faecalibacterium	2	6	1	1	27	11	6
Filimonas	0	0	5	0	0	0	0
Flavobacterium	0	0	28	0	0	0	0
Fluviicola	0	0	3	0	0	0	0
Fusibacter	0	0	41	0	0	1	0
Fusicatenibacter	0	1	0	0	2	1	1
Fusobacterium	0	0	2	0	0	0	0
unclassified_c_Gammaproteobacteria	0	0	4	0	0	0	0
norank_f_Gemmatimonadaceae	0	0	0	0	1	0	0
Gemmatimonas	0	0	5	0	0	0	0
Geobacter	0	2	501	0	0	0	1
norank_p_Gracilibacteria	0	0	2	0	0	0	0
norank_f_Hyphomicrobiaceae	0	0	12	0	0	0	0
norank_o_JG30-KF-CM45	0	0	0	0	0	2	0
Kocuria	0	0	0	0	1	2	0
Kroppenstedtia	17	117	91	136	172	206	121
Lachnoclostridium	0	0	0	0	0	1	0
Lachnospiraceae_NK4A136_group	1	1	2	0	3	5	1
unclassified_f_Lachnospiraceae	0	0	1	0	1	0	1
Lactobacillus	27	32	48	20	29	33	15

Lactococcus	159	1	1	4	7	4	1
Leadbetterella	0	0	9	0	0	0	0
Lentibacillus	0	5	0	0	0	2	0
norank_f_Lentimicrobiaceae	0	0	18	0	0	2	0
Lentimicrobium	0	0	7	0	0	0	0
Leuconostoc	0	3	2	6	4	2	2
Massilia	0	2	4	1	1	0	0
Mesorhizobium	0	0	1	1	1	3	0
Methylobacterium	0	0	0	1	0	0	0
norank_f_Methylocystaceae	0	0	7	0	0	0	0
Microbispora	0	0	0	0	0	3	1
Mitochondria_norank_f_Mitochondria	35	663	492	393	520	574	145
Mobilitalea	0	0	0	0	1	0	0
Modestobacter	0	0	0	3	0	0	0
Mycobacterium	0	0	5	0	0	1	0
norank_f_NS9_marine_group	0	0	1	0	0	0	0
norank_f_Nitriliruptoraceae	0	0	0	0	0	1	0
norank_f_Nitrosomonadaceae	0	0	3	0	0	0	0
Nitrospira	0	0	1	0	0	0	0
Novosphingobium	0	9	0	0	0	0	0
OPB56_norank_f_OPB56	0	0	4	0	0	0	0
norank_o_Obscuribacterales	0	2	0	1	0	0	0
Ottowia	0	0	13	0	0	0	0
Paenibacillus	380	109	548	313	170	202	85
Paracoccus	0	0	3	5	2	1	1
Pediococcus	22	11	10	8	3	7	2
Pelomonas	1	9	3	6	10	10	10
Peredibacter	0	0	0	1	0	1	0
Perlucidibaca	0	0	0	0	0	1	1
Phaselicystis	0	0	5	0	0	0	0
Phormidium	0	0	0	0	0	3	0
unclassified_p_Planctomycetes	0	0	0	0	0	1	0
norank_f_Porphyromonadaceae	0	0	25	0	0	0	0
Prevotella_9	0	4	0	2	6	4	3
Prevotellaceae_UCG-001	0	0	0	0	0	1	0
Propionibacterium	0	2	2	1	3	3	1
Pseudomonas	0	12	8	4	3	5	3
RB41	0	1	0	0	0	3	0
Ralstonia	31	137	82	177	196	277	173
Rhizobium	0	1	8	0	0	2	1
Rhodobacter	0	0	3	0	0	2	0
Rhodococcus	18	154	53	130	145	235	189
unclassified_f_Rhodocyclaceae	0	0	6	0	0	0	0
Romboutsia	1	2	1	1	5	2	2

Roseburia	0	1	0	0	2	2	3
Ruminiclostridium_5	0	1	0	0	0	0	0
Ruminococcaceae_NK4A214_group	0	0	0	0	0	2	0
Ruminococcus_2	0	5	0	0	2	3	0
norank_c_SBR2076	0	0	12	0	0	0	0
norank_c_SJA-15	0	0	7	0	0	0	0
SM1A02	0	0	0	0	0	2	0
Saccharopolyspora	5	18	31	62	39	96	58
Shinella	0	0	12	0	0	2	1
Solibacillus	0	0	0	1	0	1	0
Solirubrobacter	0	0	0	0	0	0	2
unclassified_f_Sphingobacteriaceae	0	0	5	0	0	0	0
norank_o_Sphingobacteriales	0	0	72	0	0	0	0
Sphingobacterium	0	0	0	1	0	1	0
Sphingobium	1	2	0	0	2	0	2
Sporacetigenium	0	0	0	0	0	0	0
Staphylococcus	3	19	10	9	5	8	4
Stenotrophomonas	0	1	2	1	1	1	2
Streptococcus	0	0	0	0	1	2	1
Streptomyces	16	78	106	193	143	205	218
Subdoligranulum	1	2	0	0	3	2	1
Sulfuritalea	0	0	1	1	0	0	0
norank_c_TK10	0	0	0	0	0	1	0
Taibaiella	0	0	4	0	0	0	0
Thauera	0	0	4	0	0	1	0
Thermoactinomyces	1	0	0	2	1	3	1
Thermomonas	0	0	0	0	0	0	1
Truepera	0	0	2	0	0	0	0
Turicibacter	0	1	0	0	1	2	0
Veillonella	0	0	0	0	2	2	1
Virgibacillus	1	0	1	2	4	6	3
Weissella	71	656	543	735	499	239	149
[Eubacterium]_coprostanoligenes_group	0	1	2	0	3	1	1
[Eubacterium]_eligens_group	0	0	0	0	4	0	0
[Eubacterium]_hallii_group	0	1	0	0	3	1	1
[Eubacterium]_rectale_group	0	2	1	2	10	3	4
[Ruminococcus]_gnavus_group	0	0	0	0	2	0	1
cvE6_norank_f_cvE6	0	0	0	1	0	0	0
vadinBC27_wastewater-sludge_group	0	0	45	0	0	0	0

Table S5. Predicted gene functions related to KEGG pathways at levels 3

KEGG pathways at levels 3	D0	D2	D4	D6	D8	D10	D12
1,1,1-Trichloro-2,2-bis(4-chlorophenyl)ethane (DDT) degradation	23	125	89	137	147	223	156
ABC transporters	926311	831619	770254	885626	862443	975038	915906
Adherens junction	0	0	0	0	0	0	0
Adipocytokine signaling pathway	13873	13068	12267	14032	13881	15662	14560
African trypanosomiasis	34	693	581	385	528	622	193
Alanine, aspartate and glutamate metabolism	246576	219965	200919	232570	225164	257603	243250
Aldosterone-regulated sodium reabsorption	0	0	0	0	0	0	0
Alzheimer's disease	13783	15190	13764	14379	14174	16893	14136
Amino acid metabolism	82186	73445	67160	78100	76355	85567	80816
Amino acid related enzymes	309959	291414	265621	301173	294377	335010	307327
Amino sugar and nucleotide sugar metabolism	341063	301596	274127	316884	299734	350596	332860
Aminoacyl-tRNA biosynthesis	209398	208969	189707	213607	213690	236117	210500
Aminobenzoate degradation	130078	112380	104059	121251	118167	135329	129300
Amoebiasis	20169	16840	15297	17887	16867	19890	19288
Amyotrophic lateral sclerosis (ALS)	33951	27969	26012	30080	29480	33419	32202
Antigen processing and presentation	6769	6758	5992	6585	5995	7607	6824
Apoptosis	60	839	789	553	645	692	220
Arachidonic acid metabolism	20808	18223	17060	19829	20072	21519	20345
Arginine and proline metabolism	337099	303791	277909	322104	314872	354969	333515
Arrhythmogenic right ventricular cardiomyopathy (ARVC)	0	0	0	0	0	0	0
Ascorbate and aldarate metabolism	80361	67475	61300	71033	64886	80391	77985
Atrazine degradation	472	2773	2233	2634	2718	2779	1819
Bacterial chemotaxis	183560	150302	144148	162078	155839	180005	174581
Bacterial invasion of epithelial cells	81	676	437	585	665	519	288
Bacterial motility proteins	385201	333216	315502	359273	355551	393203	373877
Bacterial secretion system	97049	103818	94048	101453	100046	115729	99514
Bacterial toxins	20735	18336	17005	19644	19467	21387	20254
Basal transcription factors	49	90	123	134	128	172	122
Base excision repair	90793	85140	78648	90014	90569	99000	90712
Benzoate degradation	97455	87147	82878	95277	94697	106838	100509
Betalain biosynthesis	10	47	57	76	69	101	79
Bile secretion	7	34	51	77	56	83	82
Biosynthesis and biodegradation of secondary metabolites	13745	13070	11649	13695	12850	15376	14326
Biosynthesis of 12-, 14- and 16-membered macrolides	19	87	121	214	159	237	235
Biosynthesis of ansamycins	20321	17132	15905	18244	17296	20407	19667
Biosynthesis of siderophore group nonribosomal peptides	46517	38217	34374	39905	35933	45309	44420
Biosynthesis of type II polyketide backbone	15	80	110	195	145	225	215

Biosynthesis of type II polyketide products	4	23	43	48	36	59	51
Biosynthesis of unsaturated fatty acids	82005	71944	66275	76785	74004	85411	81142
Biosynthesis of vancomycin group antibiotics	277	1685	1563	1578	1696	1488	872
Biotin metabolism	40403	36680	32861	37655	34824	42454	40034
Bisphenol degradation	60565	50966	46367	54201	50353	61523	59683
Bladder cancer	15	90	120	116	111	168	128
Butanoate metabolism	219874	196630	183016	209560	204183	235063	221151
Butirosin and neomycin biosynthesis	7321	7355	6895	8085	8640	8409	7566
C5-Branched dibasic acid metabolism	68241	61474	56239	63819	60055	71909	67470
CAM ligands	0	0	0	0	0	0	0
Caffeine metabolism	7	52	37	67	59	97	82
Calcium signaling pathway	78	671	434	579	636	513	284
Caprolactam degradation	21027	20366	19683	22318	22463	25921	23907
Carbohydrate digestion and absorption	7105	6273	5797	6970	7513	7308	6857
Carbohydrate metabolism	74415	65193	58328	68218	63189	75856	72617
Carbon fixation in photosynthetic organisms	84401	87995	79356	90115	90799	98212	87049
Carbon fixation pathways in prokaryotes	221520	204533	190130	215644	215471	239693	221034
Cardiac muscle contraction	116	2072	1858	1202	1621	1918	574
Carotenoid biosynthesis	663	5586	3982	4875	5297	4437	2501
Cell cycle	0	0	0	0	0	0	0
Cell cycle - Caulobacter	97368	97663	88700	99171	99225	110092	97952
Cell cycle - yeast	0	0	0	0	0	0	0
Cell division	13585	13026	11919	13229	12640	14946	13671
Cell motility and secretion	8118	14676	13670	13746	15215	14744	10433
Cellular antigens	13860	12628	11799	13485	13286	14764	13841
Chagas disease (American trypanosomiasis)	34	692	577	383	527	620	191
Chaperones and folding catalysts	167366	170199	154762	173896	173130	191153	170433
Chloroalkane and chloroalkene degradation	82430	73524	66991	78326	75560	86933	82379
Chlorocyclohexane and chlorobenzene degradation	13605	13808	12222	14035	12601	16225	14892
Cholinergic synapse	0	0	0	0	0	0	0
Chromosome	377331	349581	316721	362639	350525	402029	372822
Chronic myeloid leukemia	0	0	0	0	0	0	0
Circadian rhythm - plant	6801	5472	5114	5968	5795	6583	6418
Citrate cycle (TCA cycle)	171728	160272	147514	166011	163612	187558	171653
Clavulanic acid biosynthesis	0	0	0	0	0	0	0
Colorectal cancer	31	666	621	360	498	575	149
Complement and coagulation cascades	0	0	0	0	0	0	0
Cyanoamino acid metabolism	82014	71955	65719	76137	73046	84512	80296
Cysteine and methionine metabolism	254654	222986	205673	237896	234232	262378	248322
Cytochrome P450	7	66	24	52	58	94	75
Cytokine receptors	0	0	0	0	0	0	0
Cytokine-cytokine receptor interaction	0	0	0	0	0	0	0
Cytoskeleton proteins	55808	55531	50063	57257	57422	62560	56382

Cytosolic DNA-sensing pathway	0	0	0	0	0	0	0
D-Alanine metabolism	40434	34118	31014	35886	33293	40030	38731
D-Arginine and D-ornithine metabolism	6767	5465	5164	5955	5827	6578	6433
D-Glutamine and D-glutamate metabolism	34415	32121	29175	33393	32489	36910	34092
DNA repair and recombination proteins	516195	501052	457079	519626	520115	571449	517001
DNA replication	111841	109603	100234	112989	114536	124561	111596
DNA replication proteins	188654	186481	169754	191487	192819	211358	189061
Dilated cardiomyopathy (DCM)	0	0	0	0	0	0	0
Dioxin degradation	27166	24124	21855	25344	23580	28278	27020
Drug metabolism - cytochrome P450	21680	24530	22064	25434	26172	27524	23925
Drug metabolism - other enzymes	68134	58030	53759	61958	58702	68778	66119
ECM-receptor interaction	0	0	0	0	0	0	0
Electron transfer carriers	13445	11557	10335	12146	11262	13600	13102
Endocrine and other factor-regulated calcium reabsorption	0	0	0	0	0	0	0
Endocytosis	4	31	13	26	29	48	38
Energy metabolism	158053	144486	135023	151820	147964	169765	158053
Epithelial cell signaling in Helicobacter pylori infection	13734	12444	11346	12975	12213	14423	13574
ErbB signaling pathway	0	0	0	0	0	0	0
Ether lipid metabolism	29	187	247	210	214	326	237
Ethylbenzene degradation	21381	18475	17609	20563	21692	22353	21051
Fat digestion and absorption	0	0	0	0	0	0	0
Fatty acid biosynthesis	158953	142314	131683	151735	150245	167335	156674
Fatty acid elongation in mitochondria	0	0	2	0	0	0	0
Fatty acid metabolism	138567	127441	118275	137178	136154	153487	143510
Fc epsilon RI signaling pathway	0	0	0	0	0	0	0
Fc gamma R-mediated phagocytosis	4	31	13	26	29	48	38
Flagellar assembly	224281	181195	171907	196968	191550	218023	212328
Flavone and flavonol biosynthesis	41	17	79	35	33	37	18
Flavonoid biosynthesis	7073	7522	6568	7751	7738	8183	7312
Fluorobenzoate degradation	262	2009	1703	1908	1997	2182	1404
Focal adhesion	0	0	0	0	0	0	0
Folate biosynthesis	123202	111122	101133	116772	112028	129012	121421
Fructose and mannose metabolism	217563	191227	172623	200495	189262	222684	212227
Function unknown	387083	347860	322289	370852	365997	406347	381054
G protein-coupled receptors	0	0	0	0	0	0	0
GTP-binding proteins	0	0	0	0	0	0	0
Galactose metabolism	136103	116955	107406	123979	116428	137589	132107
Gastric acid secretion	0	0	0	0	0	0	0
General function prediction only	898329	821745	754005	869349	851079	954486	891370
Geraniol degradation	61961	55056	52122	60104	59349	68145	64490
Germination	141697	113476	104053	121541	113144	135697	133028
Glioma	0	0	0	0	0	0	0

Glutamatergic synapse	20574	19522	17480	20176	19427	22457	20724
Glutathione metabolism	63405	64260	58933	67116	67375	72624	65284
Glycan bindng proteins	0	0	0	0	0	0	0
Glycan biosynthesis and metabolism	246	1626	1623	1485	1630	1502	852
Glycerolipid metabolism	96344	86880	79211	91930	88461	101520	95556
Glycerophospholipid metabolism	130908	119734	110036	125333	123173	139203	129061
Glycine, serine and threonine metabolism	205437	185191	170855	194891	188188	217727	204039
Glycolysis / Gluconeogenesis	296318	270874	246434	285555	278238	314427	293975
Glycosaminoglycan biosynthesis - chondroitin sulfate	0	0	0	0	0	0	0
Glycosaminoglycan degradation	66	120	525	247	273	334	261
Glycosphingolipid biosynthesis - ganglio series	19	81	369	159	183	223	187
Glycosphingolipid biosynthesis - globo series	6964	5685	5903	6378	6208	7093	6840
Glycosphingolipid biosynthesis - lacto and neolacto series	0	1	1	0	2	0	0
Glycosylphosphatidylinositol(GPI)-anchor biosynthesis	0	0	3	0	0	0	0
Glycosyltransferases	62685	65153	57481	66485	63515	71938	65011
Glyoxylate and dicarboxylate metabolism	176906	158427	146198	166524	159145	187171	176199
GnRH signaling pathway	4	31	13	26	29	48	38
Hedgehog signaling pathway	0	0	0	0	0	0	0
Hematopoietic cell lineage	0	0	2	0	0	0	0
Hepatitis C	0	0	0	0	0	0	0
Histidine metabolism	125169	112428	104908	120982	120815	132462	124297
Homologous recombination	133034	135350	122647	137760	138611	152063	134639
Huntington's disease	20777	21268	19402	21084	21467	24040	20844
Hypertrophic cardiomyopathy (HCM)	0	2	3	2	1	2	3
Indole alkaloid biosynthesis	3	15	35	34	25	38	39
Influenza A	34	679	639	392	522	609	185
Inorganic ion transport and metabolism	34742	32760	31857	34680	33655	38864	35758
Inositol phosphate metabolism	87370	74769	67761	78403	72232	88650	85151
Insulin signaling pathway	7630	9946	8662	10204	11113	10408	8629
Ion channels	6828	6442	5569	6586	5781	7226	6832
Isoflavonoid biosynthesis	193	128	290	427	1086	221	145
Isoquinoline alkaloid biosynthesis	7204	7320	7096	7799	8523	8597	7507
Leishmaniasis	0	0	0	0	0	1	0
Leukocyte transendothelial migration	0	0	0	0	0	0	0
Limonene and pinene degradation	108937	93682	86759	100732	96768	113975	109247
Linoleic acid metabolism	20392	17206	15741	18322	17284	20803	20125
Lipid biosynthesis proteins	193602	177360	163795	188640	186113	209102	194724
Lipid metabolism	14726	17025	15476	17776	19351	19211	16220
Lipoic acid metabolism	20693	20675	18352	20960	20342	23235	20912
Lipopolysaccharide biosynthesis	1004	5824	6980	5700	6765	5745	3269
Lipopolysaccharide biosynthesis proteins	8130	12758	13820	13081	13993	13568	10428

Long-term depression	0	0	0	0	0	0	0
Long-term potentiation	0	0	0	0	0	0	0
Lysine biosynthesis	138688	129920	119174	135461	134451	150239	137773
Lysine degradation	89651	80147	75564	86591	86375	97276	91368
Lysosome	190	216	965	387	411	515	354
MAPK signaling pathway	0	0	0	0	0	0	0
MAPK signaling pathway - yeast	14153	11959	11500	13440	14609	14259	13487
Measles	0	0	0	0	0	0	0
Meiosis - yeast	6889	8201	6958	8009	7447	8731	7664
Melanogenesis	7	32	20	42	44	63	40
Membrane and intracellular structural molecules	109881	104879	97315	107801	105313	120488	110079
Metabolism of cofactors and vitamins	40984	37052	33814	38868	36452	43351	40954
Metabolism of xenobiotics by cytochrome P450	21432	24246	21590	24885	24951	27079	23645
Methane metabolism	220103	202853	185771	213466	205776	235814	220502
Mineral absorption	7096	7596	6665	7855	7860	8318	7401
Mismatch repair	139517	137536	124986	141329	141505	155848	139775
N-Glycan biosynthesis	6836	7406	6340	7344	6708	8025	7216
NOD-like receptor signaling pathway	6770	6758	5992	6585	6021	7609	6826
Naphthalene degradation	82418	72429	66303	77578	74984	86448	82326
Neuroactive ligand-receptor interaction	3	13	18	32	24	34	36
Neurotrophin signaling pathway	0	0	0	0	0	0	0
Nicotinate and nicotinamide metabolism	76931	72144	66772	77062	77601	83452	77174
Nitrogen metabolism	237558	205926	190151	217485	206196	242625	231762
Nitrotoluene degradation	183	1370	1928	1480	1513	1920	1334
Non-homologous end-joining	20478	16765	15840	18424	17858	20498	19843
Notch signaling pathway	0	0	0	0	0	0	0
Novobiocin biosynthesis	21040	19868	18628	21156	21770	23245	21237
Nucleotide excision repair	76393	72059	66057	75332	74824	83052	75951
Nucleotide metabolism	13324	11100	10049	11503	9906	13062	12764
Olfactory transduction	0	0	0	0	0	0	0
One carbon pool by folate	97270	93167	85437	96826	95865	106871	97407
Oocyte meiosis	0	0	0	0	0	0	0
Other glycan degradation	13947	11949	12185	12704	11190	14393	13656
Other ion-coupled transporters	615085	521165	474506	551770	522898	613389	590940
Other transporters	41579	40561	36761	41575	40208	45862	41833
Other types of O-glycan biosynthesis	0	0	0	0	0	0	0
Others	391218	344837	314742	365837	353168	402368	381547
Oxidative phosphorylation	243166	250899	226934	252998	252554	283245	249418
PPAR signaling pathway	21186	22161	20344	23456	23311	26333	23799
Pancreatic cancer	0	0	0	0	0	0	0
Pancreatic secretion	3	13	18	32	24	34	36
Pantothenate and CoA biosynthesis	124348	115502	105609	121286	118427	133527	123981
Parkinson's disease	147	2738	2481	1562	2119	2493	723
Pathogenic Escherichia coli infection	0	0	0	0	0	0	0

Pathways in cancer	13728	14289	12825	13919	13510	15954	13907
Penicillin and cephalosporin biosynthesis	13611	11820	11150	12607	11707	14182	13551
Pentose and glucuronate interconversions	228049	189838	172618	199672	181248	224622	218585
Pentose phosphate pathway	185600	168655	153029	177587	169689	194597	183209
Peptidases	461071	424792	384905	445851	436213	489723	456447
Peptidoglycan biosynthesis	139460	135850	123749	140236	139328	154130	139355
Peroxisome	75374	67015	62020	71200	69590	79213	74634
Pertussis	114	904	915	802	862	842	482
Phagosome	0	0	0	0	0	0	0
Phenylalanine metabolism	36586	36481	35776	40740	44998	44338	39630
Phenylalanine, tyrosine and tryptophan biosynthesis	146338	133308	123522	142489	143312	155763	145212
Phenylpropanoid biosynthesis	34309	30188	27671	31900	30160	35388	33632
Phosphatidylinositol signaling system	21048	21229	19316	21847	21813	23814	21277
Phosphonate and phosphinate metabolism	13602	11109	10608	11865	10982	13470	13048
Phosphotransferase system (PTS)	346560	279283	251470	294258	267868	331991	326845
Photosynthesis	60303	95969	76553	91194	94363	93555	72807
Photosynthesis - antenna proteins	1330	11407	7382	9847	10816	8717	4794
Photosynthesis proteins	68529	113590	89608	107701	111749	109500	84392
Phototransduction	0	0	0	0	0	0	0
Phototransduction - fly	0	0	0	0	0	0	0
Plant-pathogen interaction	27278	25095	23071	25787	24321	29113	27006
Polycyclic aromatic hydrocarbon degradation	47850	42998	39126	45772	43564	50884	48284
Polyketide sugar unit biosynthesis	1102	5993	5521	5635	6004	5178	3057
Pores ion channels	29309	36718	34239	36944	37568	38995	33028
Porphyrin and chlorophyll metabolism	148962	166751	147726	168959	171550	183123	158995
Prenyltransferases	69971	71255	63976	73121	72599	80024	71566
Primary bile acid biosynthesis	6641	5756	5074	5916	5132	6881	6683
Primary immunodeficiency	7010	6489	5986	7026	6925	7768	7248
Prion diseases	6981	5609	5416	6390	6887	6831	6573
Progesterone-mediated oocyte maturation	6769	6758	5992	6585	5995	7607	6824
Propanoate metabolism	226928	199311	186014	214415	211975	239410	226388
Prostate cancer	6769	6758	5992	6585	5998	7608	6825
Proteasome	6800	6965	6176	6889	6263	8033	7194
Protein digestion and absorption	8	45	185	88	85	113	97
Protein export	137217	131951	118693	134177	129634	150329	136690
Protein folding and associated processing	86178	94778	86782	97802	101274	105638	91880
Protein kinases	224227	195724	177968	206138	194650	228242	217632
Protein processing in endoplasmic reticulum	20348	19696	17493	19926	18553	22328	20537
Proximal tubule bicarbonate reclamation	13466	11669	10632	12294	11409	13832	13264
Purine metabolism	450099	423491	386012	442338	432525	486086	448191
Pyrimidine metabolism	326963	309036	282760	322587	320819	354307	324141
Pyruvate metabolism	329936	296652	271805	314157	307574	346892	325721
RIG-I-like receptor signaling pathway	81	71	106	107	108	129	96

RNA degradation	83816	84733	76371	86848	86511	95121	85180
RNA polymerase	34876	34514	31042	35425	35426	38872	34866
RNA transport	27738	24732	22703	26440	25969	28509	26912
Regulation of actin cytoskeleton	0	0	0	0	0	0	0
Renal cell carcinoma	6928	6865	6212	6974	7017	7771	6934
Renin-angiotensin system	0	3	8	4	2	5	7
Replication, recombination and repair proteins	121207	121451	110023	125504	124077	136090	122101
Restriction enzyme	40522	35223	31824	36758	34398	40997	39255
Retinol metabolism	21003	20457	18276	21715	21425	23728	21892
Rheumatoid arthritis	0	0	0	0	0	0	0
Riboflavin metabolism	55525	53237	48083	55513	54546	60848	55876
Ribosome	412634	410222	366869	411748	395746	461477	414254
Ribosome Biogenesis	249152	237467	216653	244971	239619	271013	247488
Ribosome biogenesis in eukaryotes	7002	7162	6585	7311	7347	8132	7179
Salivary secretion	0	0	0	0	0	0	0
Secondary bile acid biosynthesis	6604	5458	4826	5596	4819	6366	6268
Secretion system	325516	313129	287715	323405	321464	357711	324785
Selenocompound metabolism	97134	87484	80861	93227	92660	102205	95519
Sesquiterpenoid biosynthesis	6	26	36	64	48	69	73
Shigellosis	0	0	0	0	0	0	0
Signal transduction mechanisms	157990	142509	129376	150366	145708	165176	155465
Small cell lung cancer	31	666	621	360	498	576	149
Sphingolipid metabolism	13811	12137	11875	12967	12143	14551	13798
Spliceosome	0	0	0	0	0	0	0
Sporulation	671992	541543	492818	579500	547001	646896	634171
Staphylococcus aureus infection	46129	36994	32791	38102	32687	43762	43366
Starch and sucrose metabolism	305969	263732	238853	278648	264187	308314	295733
Steroid biosynthesis	7004	7010	6117	7391	7316	7994	7312
Steroid hormone biosynthesis	128	1073	889	993	1058	1195	809
Stilbenoid, diarylheptanoid and gingerol biosynthesis	26872	23364	20907	24721	22861	27812	26792
Streptomycin biosynthesis	29027	33116	30147	34232	34216	36311	31605
Styrene degradation	14011	12749	12175	14059	14407	15612	14632
Sulfur metabolism	102059	89407	81503	94083	88768	104770	99895
Sulfur relay system	88671	81970	73727	84803	80413	94644	88414
Synthesis and degradation of ketone bodies	14797	13588	13910	15998	18130	17292	15887
Systemic lupus erythematosus	0	2	3	1	0	1	0
TGF-beta signaling pathway	0	0	0	0	0	0	0
Taurine and hypotaurine metabolism	41034	35592	32725	37981	36555	41898	40062
Terpenoid backbone biosynthesis	104612	99394	90898	104983	103874	114505	105531
Tetracycline biosynthesis	55003	47846	43878	51202	50377	56060	53326
Thiamine metabolism	123107	109016	99218	114951	110124	126916	120276
Tight junction	0	0	0	0	0	0	0
Toluene degradation	27658	28433	25817	28807	28326	32978	29222

Toxoplasmosis	31	666	621	360	498	575	149
Transcription factors	611253	518852	475655	551222	520425	614021	590441
Transcription machinery	207215	189990	175790	199890	196783	221074	204913
Transcription related proteins	6619	5309	4719	5472	4738	6306	6232
Translation factors	69526	72578	65166	72407	70418	80671	71054
Translation proteins	160599	154986	141965	161370	161733	176507	160346
Transporters	1949786	1709094	1575307	1815184	1733563	2009635	1905726
Tropane, piperidine and pyridine alkaloid biosynthesis	21107	20247	18972	21592	22182	23891	21771
Tryptophan metabolism	124657	110399	104172	120310	121300	133956	126012
Tuberculosis	21657	26772	23177	26475	27066	28341	23678
Two-component system	788410	682057	628198	721710	685955	801033	765201
Type I diabetes mellitus	6927	7739	6722	7530	6982	8522	7391
Type II diabetes mellitus	7126	7820	6901	8074	8107	8515	7537
Tyrosine metabolism	91844	85796	79775	92963	95237	100650	93112
Ubiquinone and other terpenoid-quinone biosynthesis	83476	85696	76210	87032	86299	96192	85696
Ubiquitin system	13419	11470	10307	12048	11199	13470	12997
VEGF signaling pathway	0	0	0	0	0	1	0
Valine, leucine and isoleucine biosynthesis	157420	146664	132932	151363	143388	169599	157486
Valine, leucine and isoleucine degradation	186690	165785	156246	179651	180930	200213	188113
Various types of N-glycan biosynthesis	0	0	0	0	0	0	0
Vascular smooth muscle contraction	0	0	0	0	0	0	0
Vasopressin-regulated water reabsorption	0	0	8	0	0	1	0
Vibrio cholerae infection	0	0	7	0	0	0	0
Vibrio cholerae pathogenic cycle	20605	19837	17738	20487	19745	22357	20731
Viral myocarditis	31	666	621	360	498	575	149
Vitamin B6 metabolism	47025	41783	37577	43059	38563	48719	46518
Wnt signaling pathway	0	0	0	0	0	0	0
Xylene degradation	13769	13268	11895	13820	13007	15448	14439
Zeatin biosynthesis	6793	6931	6111	6751	6168	7716	6889
alpha-Linolenic acid metabolism	7157	6752	6359	7502	8028	8121	7482
beta-Alanine metabolism	83063	75489	70848	81775	81597	91345	85702
beta-Lactam resistance	13503	11649	10658	12239	11420	13763	13206
mRNA surveillance pathway	4	31	14	26	29	49	38
mTOR signaling pathway	0	0	0	0	0	0	0
p53 signaling pathway	31	666	653	360	498	577	149

Table S6. Fungi genera abundance during different fermentation stages of *F. tataricum.*

OTU ID	D0	D2	D4	D6	D8	D10	D12
Aspergillus	975	88	19	6	8	14	17
Cladosporium	63	1	0	0	0	0	0
Cochliobolus	10	4	0	0	0	0	0
norank_k_Fungi	818	221	14	47	128	55	82
norank_o_Hypocreales	4	0	0	0	0	0	0
unclassified_o_Hypocreales	8	0	1	0	0	1	0
norank_o_Malasseziales	4	0	0	0	0	0	0
Pichia	10865	193	30	37	46	56	63
unclassified_o_Saccharomycetales	34042	44968	52779	47571	47037	47961	48474
Saccharomycopsis	421	62	7	9	10	9	6
Sporidiobolus	9	0	0	0	0	0	0
norank_o_Tremellales	4	0	0	0	0	0	0

Table S7. The correlation between microorganism and flavor compounds during *F. tataricum* grain fermentation

Microorganism	Flavor compounds	P value	P adjust	Correlation
[Eubacterium]_eligens_group	1,54-dibromo-Tetrapentacontane	1.04E-05	0.000338	0.992157
[Eubacterium]_eligens_group	2,6,10,15-tetramethyl-Heptadecane	6.76E-05	0.001965	0.983394
Acetoanaerobium	2,4,4-trimethyl-Hexane	4.08E-09	1.71E-07	0.999661
Acetoanaerobium	3,5-Dimethyl-4-octanone	4.08E-09	1.71E-07	0.999661
Acetoanaerobium	3-Nonene	4.08E-09	1.71E-07	0.999661
Acetoanaerobium	Dodecanoic acid ethyl ester	0.001909	0.044668	0.936186
Acetoanaerobium	Ethyl 9-hexadecenoate	4.08E-09	1.71E-07	0.999661
Acetoanaerobium	Oxybis dichloro-Methane	4.08E-09	1.71E-07	0.999661
Acetoanaerobium	Pentadecanoic acid ethyl ester	4.08E-09	1.71E-07	0.999661
Alistipes	1,54-dibromo-Tetrapentacontane	0.000141	0.004081	0.977702
Alistipes	2,6,10,15-tetramethyl-Heptadecane	0.000279	0.007517	0.970631
Anaerobaculum	1-Heptanol	0	0	1
Anaerobaculum	2-(1-methylethoxy)-Ethanol	0	0	1
Anaerobaculum	3-methyl-Uecane	0	0	1
Anaerobaculum	Acetyl valeryl	0	0	1
Anaerobaculum	Amyl Nitrite	0	0	1
Anaerobaculum	diethyl(decyloxy)-Borane	0	0	1
Anaerobaculum	Oxalic acid-6-ethyloct-3-yl heptyl ester	0	0	1
Anaerovorax	2,4,4-trimethyl-Hexane	0	0	1
Anaerovorax	3,5-Dimethyl-4-octanone	0	0	1
Anaerovorax	3-Nonene	0	0	1
Anaerovorax	Dodecanoic acid ethyl ester	0.001756	0.041348	0.938306
Anaerovorax	Ethyl 9-hexadecenoate	0	0	1
Anaerovorax	Oxybis dichloro-Methane	0	0	1

Anaerovorax	Pentadecanoic acid ethyl ester	0	0	1
Aspergillus	1,2-Butanediol	1.05E-06	3.77E-05	0.996876
Aspergillus	1,3-dihydroxy-2-Propanone	1.05E-06	3.77E-05	0.996876
Aspergillus	1-bromo-2-methyl-Decane	1.05E-06	3.77E-05	0.996876
Aspergillus	1-Heptadecanamine	1.05E-06	3.77E-05	0.996876
Aspergillus	1-hydroxy-2-Propanone	1.05E-06	3.77E-05	0.996876
Aspergillus	1-Octen-3-ol	1.05E-06	3.77E-05	0.996876
Aspergillus	2,2'-oxybis-Pentane	1.05E-06	3.77E-05	0.996876
Aspergillus	2,6-dimethyl-Pyrazine	1.05E-06	3.77E-05	0.996876
Aspergillus	2-ethenyl-5-methyl-Pyrazine	1.05E-06	3.77E-05	0.996876
Aspergillus	2-ethyl-3,5-dimethyl-Pyrazine	1.05E-06	3.77E-05	0.996876
Aspergillus	2-ethyl-5-methyl-Pyrazine	1.05E-06	3.77E-05	0.996876
Aspergillus	2-ethyl-6-methyl-Pyrazine	1.05E-06	3.77E-05	0.996876
Aspergillus	2-methyl-3-Hexanone	1.05E-06	3.77E-05	0.996876
Aspergillus	3,5-diethyl-2-methyl-Pyrazine	1.05E-06	3.77E-05	0.996876
Aspergillus	3,8-dimethyl-Decane	1.05E-06	3.77E-05	0.996876
Aspergillus	3-hydroxy-2-Butanone	0.000386	0.010367	0.966553
Aspergillus	2-(2-butoxyethoxy)-Ethanol	1.05E-06	3.77E-05	0.996876
Aspergillus	ethyl-Pyrazine	1.05E-06	3.77E-05	0.996876
Aspergillus	Glycolaldehyde dimer	1.05E-06	3.77E-05	0.996876
Aspergillus	Hexanal	1.05E-06	3.77E-05	0.996876
Aspergillus	(methoxymethyl)-Oxirane	1.05E-06	3.77E-05	0.996876
Aspergillus	trimethyl-Pyrazine	1.05E-06	3.77E-05	0.996876
Azospirillum	2,4,4-trimethyl-Hexane	2.35E-08	9.53E-07	0.999315
Azospirillum	3,5-Dimethyl-4-octanone	2.35E-08	9.53E-07	0.999315
Azospirillum	3-Nonene	2.35E-08	9.53E-07	0.999315
Azospirillum	Dodecanoic acid ethyl ester	0.001989	0.046386	0.935105
Azospirillum	Ethyl 9-hexadecenoate	2.35E-08	9.53E-07	0.999315
Azospirillum	Oxybis dichloro-Methane	2.35E-08	9.53E-07	0.999315
Azospirillum	Pentadecanoic acid ethyl ester	2.35E-08	9.53E-07	0.999315
Bacillus	2-methyl-Propanoic acid	0.001332	0.034003	-0.94484
Bdellovibrio	2,4,4-trimethyl-Hexane	1.72E-06	5.91E-05	0.996185
Bdellovibrio	3,5-Dimethyl-4-octanone	1.72E-06	5.91E-05	0.996185
Bdellovibrio	3-Nonene	1.72E-06	5.91E-05	0.996185
Bdellovibrio	Ethyl 9-hexadecenoate	1.72E-06	5.91E-05	0.996185
Bdellovibrio	Oxybis dichloro-Methane	1.72E-06	5.91E-05	0.996185
Bdellovibrio	Pentadecanoic acid ethyl ester	1.72E-06	5.91E-05	0.996185
Brevibacillus	2-Bromo dodecane	0.001134	0.029497	0.94832
Candidatus_Competibacter	2,4,4-trimethyl-Hexane	0	0	1
Candidatus_Competibacter	3,5-Dimethyl-4-octanone	0	0	1
Candidatus_Competibacter	3-Nonene	0	0	1
Candidatus_Competibacter	Dodecanoic acid ethyl ester	0.001756	0.041348	0.938306
Candidatus_Competibacter	Ethyl 9-hexadecenoate	0	0	1
Candidatus_Competibacter	Oxybis dichloro-Methane	0	0	1

Candidatus_Competibacter	Pentadecanoic acid ethyl ester	0	0	1
Candidatus_Microthrix	2,4,4-trimethyl-Hexane	2.95E-05	0.000861	0.988097
Candidatus_Microthrix	3,5-Dimethyl-4-octanone	2.95E-05	0.000861	0.988097
Candidatus_Microthrix	3-Nonene	2.95E-05	0.000861	0.988097
Candidatus_Microthrix	Ethyl 9-hexadecenoate	2.95E-05	0.000861	0.988097
Candidatus_Microthrix	Oxybis dichloro-Methane	2.95E-05	0.000861	0.988097
Candidatus_Microthrix	Pentadecanoic acid ethyl ester	2.95E-05	0.000861	0.988097
Cellvibrio	1-(1-methylethoxy)-2-Propanol	0	0	1
Cellvibrio	2-ethyl-Heptanoic acid	0	0	1
Cellvibrio	2-Isopropyl-5-methyl-1-heptanol	0	0	1
Cellvibrio	3-methyl-5-propyl-Nonane	0	0	1
Cellvibrio	Benzeneacetic acid ethyl ester	0	0	1
Cellvibrio	methyl 6-deoxy-alpha-L-Galactopyranoside	0	0	1
Cellvibrio	Oxalic acid- 2-ethylhexyl hexyl ester	0	0	1
Cellvibrio	Sulfurous acid ethylhexyl hexyl ester	0.000681	0.018004	0.957933
Christensenellaceae_R-7_group	2,4,4-trimethyl-Hexane	0	0	1
Christensenellaceae_R-7_group	3,5-Dimethyl-4-octanone	0	0	1
Christensenellaceae_R-7_group	3-Nonene	0	0	1
Christensenellaceae_R-7_group	Dodecanoic acid ethyl ester	0.001756	0.041348	0.938306
Christensenellaceae_R-7_group	Ethyl 9-hexadecenoate	0	0	1
Christensenellaceae_R-7_group	Oxybis dichloro-Methane	0	0	1
Christensenellaceae_R-7_group	Pentadecanoic acid ethyl ester	0	0	1
Chryseobacterium	2,4,4-trimethyl-Hexane	5.73E-06	0.000191	0.993828
Chryseobacterium	3,5-Dimethyl-4-octanone	5.73E-06	0.000191	0.993828
Chryseobacterium	3-Nonene	5.73E-06	0.000191	0.993828
Chryseobacterium	Ethyl 9-hexadecenoate	5.73E-06	0.000191	0.993828
Chryseobacterium	Oxybis dichloro-Methane	5.73E-06	0.000191	0.993828
Chryseobacterium	Pentadecanoic acid ethyl ester	5.73E-06	0.000191	0.993828
Cladosporium	1,2-Butanediol	8.24E-10	3.57E-08	0.999821
Cladosporium	1,3-dihydroxy-2-Propanone	8.24E-10	3.57E-08	0.999821
Cladosporium	1-bromo-2-methyl-Decane	8.24E-10	3.57E-08	0.999821
Cladosporium	1-Heptadecanamine	8.24E-10	3.57E-08	0.999821
Cladosporium	1-hydroxy-2-Propanone	8.24E-10	3.57E-08	0.999821
Cladosporium	1-Octen-3-ol	8.24E-10	3.57E-08	0.999821
Cladosporium	2,2'-oxybis-Pentane	8.24E-10	3.57E-08	0.999821
Cladosporium	2,6-dimethyl-Pyrazine	8.24E-10	3.57E-08	0.999821
Cladosporium	2-ethenyl-5-methyl-Pyrazine	8.24E-10	3.57E-08	0.999821
Cladosporium	2-ethyl-3,5-dimethyl-Pyrazine	8.24E-10	3.57E-08	0.999821
Cladosporium	2-ethyl-5-methyl-Pyrazine	8.24E-10	3.57E-08	0.999821
Cladosporium	2-ethyl-6-methyl-Pyrazine	8.24E-10	3.57E-08	0.999821
Cladosporium	2-methyl-3-Hexanone	8.24E-10	3.57E-08	0.999821
Cladosporium	3,5-diethyl-2-methyl-Pyrazine	8.24E-10	3.57E-08	0.999821
Cladosporium	3,8-dimethyl-Decane	8.24E-10	3.57E-08	0.999821
Cladosporium	3-hydroxy-2-Butanone	0.001024	0.026717	0.950415

Cladosporium	2-(2-butoxyethoxy)-Ethanol	8.24E-10	3.57E-08	0.999821
Cladosporium	ethyl-Pyrazine	8.24E-10	3.57E-08	0.999821
Cladosporium	Glycolaldehyde dimer	8.24E-10	3.57E-08	0.999821
Cladosporium	Hexanal	8.24E-10	3.57E-08	0.999821
Cladosporium	(methoxymethyl)-Oxirane	8.24E-10	3.57E-08	0.999821
Cladosporium	trimethyl-Pyrazine	8.24E-10	3.57E-08	0.999821
Cochliobolus	3-hydroxy-2-Butanone	0.000107	0.003103	0.980038
Comamonas	2,4,4-trimethyl-Hexane	0	0	1
Comamonas	3,5-Dimethyl-4-octanone	0	0	1
Comamonas	3-Nonene	0	0	1
Comamonas	Dodecanoic acid ethyl ester	0.001756	0.041348	0.938306
Comamonas	Ethyl 9-hexadecenoate	0	0	1
Comamonas	Oxybis dichloro-Methane	0	0	1
Comamonas	Pentadecanoic acid ethyl ester	0	0	1
Coprococcus_2	1-Heptanol	0.000227	0.006296	0.973011
Coprococcus_2	2-(1-methylethoxy)-Ethanol	0.000227	0.006296	0.973011
Coprococcus_2	3-methyl-Uecane	0.000227	0.006296	0.973011
Coprococcus_2	Acetyl valeryl	0.000227	0.006296	0.973011
Coprococcus_2	Amyl Nitrite	0.000227	0.006296	0.973011
Coprococcus_2	diethyl(decyloxy)-Borane	0.000227	0.006296	0.973011
Coprococcus_2	Oxalic acid-6-ethyloct-3-yl heptyl ester	0.000227	0.006296	0.973011
Corynebacterium	1-Heptanol	1.04E-05	0.000337	0.992171
Corynebacterium	2-(1-methylethoxy)-Ethanol	1.04E-05	0.000337	0.992171
Corynebacterium	3-methyl-Uecane	1.04E-05	0.000337	0.992171
Corynebacterium	Acetyl valeryl	1.04E-05	0.000337	0.992171
Corynebacterium	Amyl Nitrite	1.04E-05	0.000337	0.992171
Corynebacterium	diethyl(decyloxy)-Borane	1.04E-05	0.000337	0.992171
Corynebacterium	Oxalic acid-6-ethyloct-3-yl heptyl ester	1.04E-05	0.000337	0.992171
Craurococcus	1-Heptanol	1.04E-05	0.000337	0.992171
Craurococcus	2-(1-methylethoxy)-Ethanol	1.04E-05	0.000337	0.992171
Craurococcus	3-methyl-Uecane	1.04E-05	0.000337	0.992171
Craurococcus	Acetyl valeryl	1.04E-05	0.000337	0.992171
Craurococcus	Amyl Nitrite	1.04E-05	0.000337	0.992171
Craurococcus	diethyl(decyloxy)-Borane	1.04E-05	0.000337	0.992171
Craurococcus	Oxalic acid-6-ethyloct-3-yl heptyl ester	1.04E-05	0.000337	0.992171
Dechlorobacter	2,4,4-trimethyl-Hexane	1.76E-08	7.23E-07	0.99939
Dechlorobacter	3,5-Dimethyl-4-octanone	1.76E-08	7.23E-07	0.99939
Dechlorobacter	3-Nonene	1.76E-08	7.23E-07	0.99939
Dechlorobacter	Dodecanoic acid ethyl ester	0.001973	0.046067	0.935319
Dechlorobacter	Ethyl 9-hexadecenoate	1.76E-08	7.23E-07	0.99939
Dechlorobacter	Oxybis dichloro-Methane	1.76E-08	7.23E-07	0.99939
Dechlorobacter	Pentadecanoic acid ethyl ester	1.76E-08	7.23E-07	0.99939
Dechloromonas	2,4,4-trimethyl-Hexane	0	0	1
Dechloromonas	3,5-Dimethyl-4-octanone	0	0	1

Dechloromonas	3-Nonene	0	0	1
Dechloromonas	Dodecanoic acid ethyl ester	0.001756	0.041348	0.938306
Dechloromonas	Ethyl 9-hexadecenoate	0	0	1
Dechloromonas	Oxybis dichloro-Methane	0	0	1
Dechloromonas	Pentadecanoic acid ethyl ester	0	0	1
Deinococcus	2,4,4-trimethyl-Hexane	0	0	1
Deinococcus	3,5-Dimethyl-4-octanone	0	0	1
Deinococcus	3-Nonene	0	0	1
Deinococcus	Dodecanoic acid ethyl ester	0.001756	0.041348	0.938306
Deinococcus	Ethyl 9-hexadecenoate	0	0	1
Deinococcus	Oxybis dichloro-Methane	0	0	1
Deinococcus	Pentadecanoic acid ethyl ester	0	0	1
Desulfatiglans	1-ethyl-2-methyl-Cyclopentane	0.001356	0.034003	0.944444
Desulfatiglans	1-Pentanol	0.000494	0.013112	0.963047
Desulfatiglans	2-methyl-Propanoic acid pentyl ester	0.000749	0.019766	0.956293
Desulfatiglans	3,5-bis(1,1-dimethylethyl)- Phenol	0.001356	0.034003	0.944444
Desulfatiglans	3,8-dimethyl-Uecane	0.001356	0.034003	0.944444
Desulfatiglans	3-ethoxy-1-Propanol	0.001356	0.034003	0.944444
Desulfatiglans	Sulfurous acid octadecyl 2-propyl ester	0.001356	0.034003	0.944444
Devosia	2,4,4-trimethyl-Hexane	3.34E-08	1.28E-06	0.999213
Devosia	3,5-Dimethyl-4-octanone	3.34E-08	1.28E-06	0.999213
Devosia	3-Nonene	3.34E-08	1.28E-06	0.999213
Devosia	Dodecanoic acid ethyl ester	0.002011	0.046771	0.934823
Devosia	Ethyl 9-hexadecenoate	3.34E-08	1.28E-06	0.999213
Devosia	Oxybis dichloro-Methane	3.34E-08	1.28E-06	0.999213
Devosia	Pentadecanoic acid ethyl ester	3.34E-08	1.28E-06	0.999213
Diaphorobacter	2,4,4-trimethyl-Hexane	5.08E-08	1.92E-06	0.999069
Diaphorobacter	3,5-Dimethyl-4-octanone	5.08E-08	1.92E-06	0.999069
Diaphorobacter	3-Nonene	5.08E-08	1.92E-06	0.999069
Diaphorobacter	Dodecanoic acid ethyl ester	0.002039	0.047225	0.934449
Diaphorobacter	Ethyl 9-hexadecenoate	5.08E-08	1.92E-06	0.999069
Diaphorobacter	Oxybis dichloro-Methane	5.08E-08	1.92E-06	0.999069
Diaphorobacter	Pentadecanoic acid ethyl ester	5.08E-08	1.92E-06	0.999069
Enhydrobacter	1-Heptanol	0.00205	0.047225	0.934311
Enhydrobacter	2-(1-methylethoxy)-Ethanol	0.00205	0.047225	0.934311
Enhydrobacter	3-methyl-Uecane	0.00205	0.047225	0.934311
Enhydrobacter	Acetyl valeryl	0.00205	0.047225	0.934311
Enhydrobacter	Amyl Nitrite	0.00205	0.047225	0.934311
Enhydrobacter	diethyl(decyloxy)-Borane	0.00205	0.047225	0.934311
Enhydrobacter	Oxalic acid-6-ethyloct-3-yl heptyl ester	0.00205	0.047225	0.934311
Enterococcus	methyl-Pyrazine	0.001591	0.039806	0.94072
Filimonas	2,4,4-trimethyl-Hexane	0	0	1
Filimonas	3,5-Dimethyl-4-octanone	0	0	1
Filimonas	3-Nonene	0	0	1

Filimonas	Dodecanoic acid ethyl ester	0.001756	0.041348	0.938306
Filimonas	Ethyl 9-hexadecenoate	0	0	1
Filimonas	Oxybis dichloro-Methane	0	0	1
Filimonas	Pentadecanoic acid ethyl ester	0	0	1
Flavobacterium	2,4,4-trimethyl-Hexane	2.49E-40	1.30E-38	1
Flavobacterium	3,5-Dimethyl-4-octanone	1.41E-39	6.85E-38	1
Flavobacterium	3-Nonene	0	0	1
Flavobacterium	Dodecanoic acid ethyl ester	0.001756	0.041348	0.938306
Flavobacterium	Ethyl 9-hexadecenoate	1.41E-39	6.85E-38	1
Flavobacterium	Oxybis dichloro-Methane	1.41E-39	6.85E-38	1
Flavobacterium	Pentadecanoic acid ethyl ester	0	0	1
Fluviicola	2,4,4-trimethyl-Hexane	0	0	1
Fluviicola	3,5-Dimethyl-4-octanone	0	0	1
Fluviicola	3-Nonene	0	0	1
Fluviicola	Dodecanoic acid ethyl ester	0.001756	0.041348	0.938306
Fluviicola	Ethyl 9-hexadecenoate	0	0	1
Fluviicola	Oxybis dichloro-Methane	0	0	1
Fluviicola	Pentadecanoic acid ethyl ester	0	0	1
Fusibacter	2,4,4-trimethyl-Hexane	1.18E-08	4.90E-07	0.999481
Fusibacter	3,5-Dimethyl-4-octanone	1.18E-08	4.90E-07	0.999481
Fusibacter	3-Nonene	1.18E-08	4.90E-07	0.999481
Fusibacter	Dodecanoic acid ethyl ester	0.001953	0.045647	0.93559
Fusibacter	Ethyl 9-hexadecenoate	1.18E-08	4.90E-07	0.999481
Fusibacter	Oxybis dichloro-Methane	1.18E-08	4.90E-07	0.999481
Fusibacter	Pentadecanoic acid ethyl ester	1.18E-08	4.90E-07	0.999481
Fusobacterium	2,4,4-trimethyl-Hexane	0.000158	0.004541	0.976624
Fusobacterium	3,5-Dimethyl-4-octanone	0.000158	0.004541	0.976624
Fusobacterium	3-Nonene	0.000158	0.004541	0.976624
Fusobacterium	Ethyl 9-hexadecenoate	0.000158	0.004541	0.976624
Fusobacterium	Oxybis dichloro-Methane	0.000158	0.004541	0.976624
Fusobacterium	Pentadecanoic acid ethyl ester	0.000158	0.004541	0.976624
Gemmatimonas	2,4,4-trimethyl-Hexane	0	0	1
Gemmatimonas	3,5-Dimethyl-4-octanone	0	0	1
Gemmatimonas	3-Nonene	0	0	1
Gemmatimonas	Dodecanoic acid ethyl ester	0.001756	0.041348	0.938306
Gemmatimonas	Ethyl 9-hexadecenoate	0	0	1
Gemmatimonas	Oxybis dichloro-Methane	0	0	1
Gemmatimonas	Pentadecanoic acid ethyl ester	0	0	1
Geobacter	2,4,4-trimethyl-Hexane	4.06E-13	1.94E-11	0.999991
Geobacter	3,5-Dimethyl-4-octanone	4.06E-13	1.94E-11	0.999991
Geobacter	3-Nonene	4.06E-13	1.94E-11	0.999991
Geobacter	Dodecanoic acid ethyl ester	0.001792	0.04209	0.937798
Geobacter	Ethyl 9-hexadecenoate	4.06E-13	1.94E-11	0.999991
Geobacter	Oxybis dichloro-Methane	4.06E-13	1.94E-11	0.999991

Geobacter	Pentadecanoic acid ethyl ester	4.06E-13	1.94E-11	0.999991
Kocuria	1-Heptanol	0.001732	0.041348	0.938652
Kocuria	2-(1-methylethoxy)-Ethanol	0.001732	0.041348	0.938652
Kocuria	3-methyl-Uecane	0.001732	0.041348	0.938652
Kocuria	Acetyl valeryl	0.001732	0.041348	0.938652
Kocuria	Amyl Nitrite	0.001732	0.041348	0.938652
Kocuria	diethyl(decyloxy)-Borane	0.001732	0.041348	0.938652
Kocuria	Oxalic acid-6-ethyloct-3-yl heptyl ester	0.001732	0.041348	0.938652
Lactococcus	1,2-Butanediol	2.80E-08	1.08E-06	0.999266
Lactococcus	1,3-dihydroxy-2-Propanone	2.80E-08	1.08E-06	0.999266
Lactococcus	1-bromo-2-methyl-Decane	2.80E-08	1.08E-06	0.999266
Lactococcus	1-Heptadecanamine	2.80E-08	1.08E-06	0.999266
Lactococcus	1-hydroxy-2-Propanone	2.80E-08	1.08E-06	0.999266
Lactococcus	1-Octen-3-ol	2.80E-08	1.08E-06	0.999266
Lactococcus	2,2'-oxybis-Pentane	2.80E-08	1.08E-06	0.999266
Lactococcus	2,6-dimethyl-Pyrazine	2.80E-08	1.08E-06	0.999266
Lactococcus	2-ethenyl-5-methyl-Pyrazine	2.80E-08	1.08E-06	0.999266
Lactococcus	2-ethyl-3,5-dimethyl-Pyrazine	2.80E-08	1.08E-06	0.999266
Lactococcus	2-ethyl-5-methyl-Pyrazine	2.80E-08	1.08E-06	0.999266
Lactococcus	2-ethyl-6-methyl-Pyrazine	2.80E-08	1.08E-06	0.999266
Lactococcus	2-methyl-3-Hexanone	2.80E-08	1.08E-06	0.999266
Lactococcus	3,5-diethyl-2-methyl-Pyrazine	2.80E-08	1.08E-06	0.999266
Lactococcus	3,8-dimethyl-Decane	2.80E-08	1.08E-06	0.999266
Lactococcus	3-hydroxy-2-Butanone	0.00178	0.041852	0.937972
Lactococcus	2-(2-butoxyethoxy)-Ethanol	2.80E-08	1.08E-06	0.999266
Lactococcus	ethyl-Pyrazine	2.80E-08	1.08E-06	0.999266
Lactococcus	Glycolaldehyde dimer	2.80E-08	1.08E-06	0.999266
Lactococcus	Hexanal	2.80E-08	1.08E-06	0.999266
Lactococcus	(methoxymethyl)-Oxirane	2.80E-08	1.08E-06	0.999266
Lactococcus	trimethyl-Pyrazine	2.80E-08	1.08E-06	0.999266
Leadbetterella	2,4,4-trimethyl-Hexane	0	0	1
Leadbetterella	3,5-Dimethyl-4-octanone	0	0	1
Leadbetterella	3-Nonene	0	0	1
Leadbetterella	Dodecanoic acid ethyl ester	0.001756	0.041348	0.938306
Leadbetterella	Ethyl 9-hexadecenoate	0	0	1
Leadbetterella	Oxybis dichloro-Methane	0	0	1
Leadbetterella	Pentadecanoic acid ethyl ester	0	0	1
Lentibacillus	1-ethyl-2-methyl-Cyclopentane	0.000951	0.024898	0.951861
Lentibacillus	1-Pentanol	0.001302	0.033339	0.945354
Lentibacillus	2-methyl-Propanoic acid pentyl ester	0.001135	0.029497	0.948305
Lentibacillus	3,5-bis(1,1-dimethylethyl)- Phenol	0.000951	0.024898	0.951861
Lentibacillus	3,7-dimethyl-Nonane	0.000151	0.004369	0.977068
Lentibacillus	3,8-dimethyl-Uecane	0.000951	0.024898	0.951861
Lentibacillus	3-ethoxy-1-Propanol	0.000951	0.024898	0.951861

Lentibacillus	Sulfurous acid octadecyl 2-propyl ester	0.000951	0.024898	0.951861
Lentimicrobium	2,4,4-trimethyl-Hexane	2.49E-40	1.30E-38	1
Lentimicrobium	3,5-Dimethyl-4-octanone	0	0	1
Lentimicrobium	3-Nonene	0	0	1
Lentimicrobium	Dodecanoic acid ethyl ester	0.001756	0.041348	0.938306
Lentimicrobium	Ethyl 9-hexadecenoate	0	0	1
Lentimicrobium	Oxybis dichloro-Methane	0	0	1
Lentimicrobium	Pentadecanoic acid ethyl ester	0	0	1
Massilia	2-ethyl-2-methyl-Tridecanol	0.001726	0.041348	0.938742
Massilia	2-methyl-Propanoic acid	0.001819	0.042669	0.937422
Mesorhizobium	Butanoic acid ethyl ester	0.002071	0.047654	0.934038
Mesorhizobium	dihydro-5-propyl-2(3H)-Furanone	0.001619	0.040432	0.940313
Methylobacterium	1-iodo-Dodecane	0.001222	0.031388	0.946729
Methylobacterium	2-methyl-Butanoic acid ethyl ester	0.001222	0.031388	0.946729
Methylobacterium	2-Methylbutanoic anhydride	0.001222	0.031388	0.946729
Methylobacterium	4-methyl-Pentanoic acid ethyl ester	0.001222	0.031388	0.946729
Methylobacterium	Heneicosane	0.00136	0.034066	0.944374
Methylobacterium	Heptanoic acid ethyl ester	0.001222	0.031388	0.946729
Methylobacterium	Nonacosane	0.001222	0.031388	0.946729
Methylobacterium	Pentacosane	0.002101	0.048289	0.93365
Methylobacterium	Pentanoic acid ethyl ester	0.001222	0.031388	0.946729
Mobilitalea	1,54-dibromo-Tetrapentacontane	0	0	1
Mobilitalea	2,6,10,15-tetramethyl-Heptadecane	1.79E-06	6.13E-05	0.996124
Mobilitalea	Tetratriacontane	0.001713	0.041348	0.938921
Modestobacter	1-iodo-Dodecane	3.37E-06	0.000114	0.99501
Modestobacter	2-methyl-Butanoic acid ethyl ester	3.37E-06	0.000114	0.99501
Modestobacter	2-Methylbutanoic anhydride	3.37E-06	0.000114	0.99501
Modestobacter	3-methyl-Butanoic acid	4.10E-05	0.001195	0.986418
Modestobacter	4-methyl-Pentanoic acid ethyl ester	3.37E-06	0.000114	0.99501
Modestobacter	Heneicosane	4.56E-06	0.000154	0.994369
Modestobacter	Heptanoic acid ethyl ester	3.37E-06	0.000114	0.99501
Modestobacter	Nonacosane	3.37E-06	0.000114	0.99501
Modestobacter	Pentacosane	1.68E-05	0.000516	0.990503
Modestobacter	Pentanoic acid ethyl ester	3.37E-06	0.000114	0.99501
Mycobacterium	2,4,4-trimethyl-Hexane	2.43E-05	0.000716	0.988982
Mycobacterium	3,5-Dimethyl-4-octanone	2.43E-05	0.000716	0.988982
Mycobacterium	3-Nonene	2.43E-05	0.000716	0.988982
Mycobacterium	Ethyl 9-hexadecenoate	2.43E-05	0.000716	0.988982
Mycobacterium	Oxybis dichloro-Methane	2.43E-05	0.000716	0.988982
Mycobacterium	Pentadecanoic acid ethyl ester	2.43E-05	0.000716	0.988982
Nitrospira	2,4,4-trimethyl-Hexane	2.49E-40	1.30E-38	1
Nitrospira	3,5-Dimethyl-4-octanone	0	0	1
Nitrospira	3-Nonene	0	0	1
Nitrospira	Dodecanoic acid ethyl ester	0.001756	0.041348	0.938306

Nitrospira	Ethyl 9-hexadecenoate	0	0	1
Nitrospira	Oxybis dichloro-Methane	0	0	1
Nitrospira	Pentadecanoic acid ethyl ester	1.41E-39	6.85E-38	1
norank_c_C10-SB1A	2,4,4-trimethyl-Hexane	2.49E-40	1.30E-38	1
norank_c_C10-SB1A	3,5-Dimethyl-4-octanone	0	0	1
norank_c_C10-SB1A	3-Nonene	0	0	1
norank_c_C10-SB1A	Dodecanoic acid ethyl ester	0.001756	0.041348	0.938306
norank_c_C10-SB1A	Ethyl 9-hexadecenoate	0	0	1
norank_c_C10-SB1A	Oxybis dichloro-Methane	0	0	1
norank_c_C10-SB1A	Pentadecanoic acid ethyl ester	1.41E-39	6.85E-38	1
norank_c_SBR2076	2,4,4-trimethyl-Hexane	0	0	1
norank_c_SBR2076	3,5-Dimethyl-4-octanone	0	0	1
norank_c_SBR2076	3-Nonene	0	0	1
norank_c_SBR2076	Dodecanoic acid ethyl ester	0.001756	0.041348	0.938306
norank_c_SBR2076	Ethyl 9-hexadecenoate	0	0	1
norank_c_SBR2076	Oxybis dichloro-Methane	0	0	1
norank_c_SBR2076	Pentadecanoic acid ethyl ester	0	0	1
norank_c_SJA-15	2,4,4-trimethyl-Hexane	0	0	1
norank_c_SJA-15	3,5-Dimethyl-4-octanone	0	0	1
norank_c_SJA-15	3-Nonene	0	0	1
norank_c_SJA-15	Dodecanoic acid ethyl ester	0.001756	0.041348	0.938306
norank_c_SJA-15	Ethyl 9-hexadecenoate	0	0	1
norank_c_SJA-15	Oxybis dichloro-Methane	0	0	1
norank_c_SJA-15	Pentadecanoic acid ethyl ester	0	0	1
norank_c_TK10	1-Heptanol	0	0	1
norank_c_TK10	2-(1-methylethoxy)-Ethanol	0	0	1
norank_c_TK10	3-methyl-Uecane	0	0	1
norank_c_TK10	Acetyl valeryl	0	0	1
norank_c_TK10	Amyl Nitrite	0	0	1
norank_c_TK10	diethyl(decyloxy)-Borane	0	0	1
norank_c_TK10	Oxalic acid-6-ethyloct-3-yl heptyl ester	0	0	1
norank_f_Bacillaceae	1-(1-methylethoxy)-2-Propanol	0	0	1
norank_f_Bacillaceae	2-ethyl-Heptanoic acid	0	0	1
norank_f_Bacillaceae	2-Isopropyl-5-methyl-1-heptanol	0	0	1
norank_f_Bacillaceae	3-methyl-5-propyl-Nonane	0	0	1
norank_f_Bacillaceae	Benzeneacetic acid ethyl ester	0	0	1
norank_f_Bacillaceae	methyl 6-deoxy-alpha-L-Galactopyranoside	0	0	1
norank_f_Bacillaceae	Oxalic acid- 2-ethylhexyl hexyl ester	0	0	1
norank_f_Bacillaceae	Sulfurous acid ethylhexyl hexyl ester	0.000681	0.018004	0.957933
norank_f_Bacteroidales_RF16_group	1-Heptanol	0	0	1
norank_f_Bacteroidales_RF16_group	1-Heptanol	0	0	1
norank_f_Bacteroidales_RF16_group	2-(1-methylethoxy)-Ethanol	0	0	1
norank_f_Bacteroidales_RF16_group	2-(1-methylethoxy)-Ethanol	0	0	1
norank_f_Bacteroidales_RF16_group	3-methyl-Uecane	2.49E-40	1.30E-38	1

norank_f_Bacteroidales_RF16_group	3-methyl-Uecane	0	0	1
norank_f_Bacteroidales_RF16_group	Acetyl valeryl	1.41E-39	6.85E-38	1
norank_f_Bacteroidales_RF16_group	Acetyl valeryl	0	0	1
norank_f_Bacteroidales_RF16_group	Amyl Nitrite	0	0	1
norank_f_Bacteroidales_RF16_group	Amyl Nitrite	0	0	1
norank_f_Bacteroidales_RF16_group	diethyl(decyloxy)-Borane	1.41E-39	6.85E-38	1
norank_f_Bacteroidales_RF16_group	diethyl(decyloxy)-Borane	0	0	1
norank_f_Bacteroidales_RF16_group	Oxalic acid-6-ethyloct-3-yl heptyl ester	2.49E-40	1.30E-38	1
norank_f_Bacteroidales_RF16_group	Oxalic acid-6-ethyloct-3-yl heptyl ester	0	0	1
norank_f_Caldilineaceae	2,4,4-trimethyl-Hexane	2.49E-40	1.30E-38	1
norank_f_Caldilineaceae	3,5-Dimethyl-4-octanone	0	0	1
norank_f_Caldilineaceae	3-Nonene	0	0	1
norank_f_Caldilineaceae	Dodecanoic acid ethyl ester	0.001756	0.041348	0.938306
norank_f_Caldilineaceae	Ethyl 9-hexadecenoate	0	0	1
norank_f_Caldilineaceae	Oxybis dichloro-Methane	0	0	1
norank_f_Caldilineaceae	Pentadecanoic acid ethyl ester	0	0	1
norank_f_Chitinophagaceae	2,4,4-trimethyl-Hexane	0	0	1
norank_f_Chitinophagaceae	3,5-Dimethyl-4-octanone	0	0	1
norank_f_Chitinophagaceae	3-Nonene	0	0	1
norank_f_Chitinophagaceae	Dodecanoic acid ethyl ester	0.001756	0.041348	0.938306
norank_f_Chitinophagaceae	Ethyl 9-hexadecenoate	0	0	1
norank_f_Chitinophagaceae	Oxybis dichloro-Methane	0	0	1
norank_f_Chitinophagaceae	Pentadecanoic acid ethyl ester	0	0	1
norank_f_cvE6	1-iodo-Dodecane	0	0	1
norank_f_cvE6	2-methyl-Butanoic acid ethyl ester	1.41E-39	6.85E-38	1
norank_f_cvE6	2-Methylbutanoic anhydride	0	0	1
norank_f_cvE6	3-methyl-Butanoic acid	6.21E-06	0.000207	0.993625
norank_f_cvE6	4-methyl-Pentanoic acid ethyl ester	2.49E-40	1.30E-38	1
norank_f_cvE6	Heneicosane	1.41E-09	6.09E-08	0.999778
norank_f_cvE6	Heptanoic acid ethyl ester	0	0	1
norank_f_cvE6	Nonacosane	0	0	1
norank_f_cvE6	Pentacosane	5.43E-07	2.04E-05	0.997597
norank_f_cvE6	Pentanoic acid ethyl ester	2.49E-40	1.30E-38	1
norank_f_Elev-16S-1332	1-Heptanol	0.001356	0.034003	0.944444
norank_f_Elev-16S-1332	2-(1-methylethoxy)-Ethanol	0.001356	0.034003	0.944444
norank_f_Elev-16S-1332	3-methyl-Uecane	0.001356	0.034003	0.944444
norank_f_Elev-16S-1332	Acetyl valeryl	0.001356	0.034003	0.944444
norank_f_Elev-16S-1332	Amyl Nitrite	0.001356	0.034003	0.944444
norank_f_Elev-16S-1332	diethyl(decyloxy)-Borane	0.001356	0.034003	0.944444
norank_f_Elev-16S-1332	Oxalic acid-6-ethyloct-3-yl heptyl ester	0.001356	0.034003	0.944444
norank_f_Gemmatimonadaceae	1,54-dibromo-Tetrapentacontane	0	0	1
norank_f_Gemmatimonadaceae	2,6,10,15-tetramethyl-Heptadecane	1.79E-06	6.13E-05	0.996124
norank_f_Gemmatimonadaceae	Tetratriacontane	0.001713	0.041348	0.938921
norank_f_Hyphomicrobiaceae	2,4,4-trimethyl-Hexane	0	0	1

norank_f_Hyphomicrobiaceae	3,5-Dimethyl-4-octanone	0	0	1
norank_f_Hyphomicrobiaceae	3-Nonene	0	0	1
norank_f_Hyphomicrobiaceae	Dodecanoic acid ethyl ester	0.001756	0.041348	0.938306
norank_f_Hyphomicrobiaceae	Ethyl 9-hexadecenoate	0	0	1
norank_f_Hyphomicrobiaceae	Oxybis dichloro-Methane	0	0	1
norank_f_Hyphomicrobiaceae	Pentadecanoic acid ethyl ester	0	0	1
norank_f_Lentimicrobiaceae	2,4,4-trimethyl-Hexane	1.14E-05	0.000365	0.991881
norank_f_Lentimicrobiaceae	3,5-Dimethyl-4-octanone	1.14E-05	0.000365	0.991881
norank_f_Lentimicrobiaceae	3-Nonene	1.14E-05	0.000365	0.991881
norank_f_Lentimicrobiaceae	Ethyl 9-hexadecenoate	1.14E-05	0.000365	0.991881
norank_f_Lentimicrobiaceae	Oxybis dichloro-Methane	1.14E-05	0.000365	0.991881
norank_f_Lentimicrobiaceae	Pentadecanoic acid ethyl ester	1.14E-05	0.000365	0.991881
norank_f_Methylocystaceae	2,4,4-trimethyl-Hexane	0	0	1
norank_f_Methylocystaceae	3,5-Dimethyl-4-octanone	0	0	1
norank_f_Methylocystaceae	3-Nonene	0	0	1
norank_f_Methylocystaceae	Dodecanoic acid ethyl ester	0.001756	0.041348	0.938306
norank_f_Methylocystaceae	Ethyl 9-hexadecenoate	0	0	1
norank_f_Methylocystaceae	Oxybis dichloro-Methane	0	0	1
norank_f_Methylocystaceae	Pentadecanoic acid ethyl ester	0	0	1
norank_f_Nitriliruptoraceae	1-Heptanol	0	0	1
norank_f_Nitriliruptoraceae	2-(1-methylethoxy)-Ethanol	0	0	1
norank_f_Nitriliruptoraceae	3-methyl-Uecane	0	0	1
norank_f_Nitriliruptoraceae	Acetyl valeryl	0	0	1
norank_f_Nitriliruptoraceae	Amyl Nitrite	0	0	1
norank_f_Nitriliruptoraceae	diethyl(decyloxy)-Borane	0	0	1
norank_f_Nitriliruptoraceae	Oxalic acid-6-ethyloct-3-yl heptyl ester	0	0	1
norank_f_Nitrosomonadaceae	2,4,4-trimethyl-Hexane	0	0	1
norank_f_Nitrosomonadaceae	3,5-Dimethyl-4-octanone	0	0	1
norank_f_Nitrosomonadaceae	3-Nonene	0	0	1
norank_f_Nitrosomonadaceae	Dodecanoic acid ethyl ester	0.001756	0.041348	0.938306
norank_f_Nitrosomonadaceae	Ethyl 9-hexadecenoate	0	0	1
norank_f_Nitrosomonadaceae	Oxybis dichloro-Methane	0	0	1
norank_f_Nitrosomonadaceae	Pentadecanoic acid ethyl ester	0	0	1
norank_f_NS9_marine_group	2,4,4-trimethyl-Hexane	2.49E-40	1.30E-38	1
norank_f_NS9_marine_group	3,5-Dimethyl-4-octanone	0	0	1
norank_f_NS9_marine_group	3-Nonene	0	0	1
norank_f_NS9_marine_group	Dodecanoic acid ethyl ester	0.001756	0.041348	0.938306
norank_f_NS9_marine_group	Ethyl 9-hexadecenoate	0	0	1
norank_f_NS9_marine_group	Oxybis dichloro-Methane	0	0	1
norank_f_NS9_marine_group	Pentadecanoic acid ethyl ester	1.41E-39	6.85E-38	1
norank_f_OPB56	2,4,4-trimethyl-Hexane	2.49E-40	1.30E-38	1
norank_f_OPB56	3,5-Dimethyl-4-octanone	0	0	1
norank_f_OPB56	3-Nonene	0	0	1
norank_f_OPB56	Dodecanoic acid ethyl ester	0.001756	0.041348	0.938306

norank_f_OPB56	Ethyl 9-hexadecenoate	0	0	1
norank_f_OPB56	Oxybis dichloro-Methane	0	0	1
norank_f_OPB56	Pentadecanoic acid ethyl ester	0	0	1
norank_f_Porphyromonadaceae	2,4,4-trimethyl-Hexane	2.49E-40	1.30E-38	1
norank_f_Porphyromonadaceae	3,5-Dimethyl-4-octanone	0	0	1
norank_f_Porphyromonadaceae	3-Nonene	0	0	1
norank_f_Porphyromonadaceae	Dodecanoic acid ethyl ester	0.001756	0.041348	0.938306
norank_f_Porphyromonadaceae	Ethyl 9-hexadecenoate	0	0	1
norank_f_Porphyromonadaceae	Oxybis dichloro-Methane	0	0	1
norank_f_Porphyromonadaceae	Pentadecanoic acid ethyl ester	2.49E-40	1.30E-38	1
norank_k_Fungi	1,2-Butanediol	0.000272	0.007338	0.970947
norank_k_Fungi	1,3-dihydroxy-2-Propanone	0.000272	0.007338	0.970947
norank_k_Fungi	1-bromo-2-methyl-Decane	0.000272	0.007338	0.970947
norank_k_Fungi	1-Heptadecanamine	0.000272	0.007338	0.970947
norank_k_Fungi	1-hydroxy-2-Propanone	0.000272	0.007338	0.970947
norank_k_Fungi	1-Octen-3-ol	0.000272	0.007338	0.970947
norank_k_Fungi	2,2'-oxybis-Pentane	0.000272	0.007338	0.970947
norank_k_Fungi	2,6-dimethyl-Pyrazine	0.000272	0.007338	0.970947
norank_k_Fungi	2-ethenyl-5-methyl-Pyrazine	0.000272	0.007338	0.970947
norank_k_Fungi	2-ethyl-3,5-dimethyl-Pyrazine	0.000272	0.007338	0.970947
norank_k_Fungi	2-ethyl-5-methyl-Pyrazine	0.000272	0.007338	0.970947
norank_k_Fungi	2-ethyl-6-methyl-Pyrazine	0.000272	0.007338	0.970947
norank_k_Fungi	2-methyl-3-Hexanone	0.000272	0.007338	0.970947
norank_k_Fungi	3,5-diethyl-2-methyl-Pyrazine	0.000272	0.007338	0.970947
norank_k_Fungi	3,8-dimethyl-Decane	0.000272	0.007338	0.970947
norank_k_Fungi	3-hydroxy-2-Butanone	0.000274	0.007389	0.97085
norank_k_Fungi	2-(2-butoxyethoxy)-Ethanol	0.000272	0.007338	0.970947
norank_k_Fungi	ethyl-Pyrazine	0.000272	0.007338	0.970947
norank_k_Fungi	Glycolaldehyde dimer	0.000272	0.007338	0.970947
norank_k_Fungi	Hexanal	0.000272	0.007338	0.970947
norank_k_Fungi	(methoxymethyl)-Oxirane	0.000272	0.007338	0.970947
norank_k_Fungi	trimethyl-Pyrazine	0.000272	0.007338	0.970947
norank_o_Hypocreales	1,2-Butanediol	0	0	1
norank_o_Hypocreales	1,3-dihydroxy-2-Propanone	0	0	1
norank_o_Hypocreales	1-bromo-2-methyl-Decane	0	0	1
norank_o_Hypocreales	1-Heptadecanamine	0	0	1
norank_o_Hypocreales	1-hydroxy-2-Propanone	0	0	1
norank_o_Hypocreales	1-Octen-3-ol	0	0	1
norank_o_Hypocreales	2,2'-oxybis-Pentane	0	0	1
norank_o_Hypocreales	2,6-dimethyl-Pyrazine	0	0	1
norank_o_Hypocreales	2-ethenyl-5-methyl-Pyrazine	0	0	1
norank_o_Hypocreales	2-ethyl-3,5-dimethyl-Pyrazine	0	0	1
norank_o_Hypocreales	2-ethyl-5-methyl-Pyrazine	0	0	1
norank_o_Hypocreales	2-ethyl-6-methyl-Pyrazine	0	0	1

norank_o_Hypocreales	2-methyl-3-Hexanone	0	0	1
norank_o_Hypocreales	3,5-diethyl-2-methyl-Pyrazine	0	0	1
norank_o_Hypocreales	3,8-dimethyl-Decane	0	0	1
norank_o_Hypocreales	3-hydroxy-2-Butanone	0.001337	0.034003	0.944762
norank_o_Hypocreales	2-(2-butoxyethoxy)-Ethanol	0	0	1
norank_o_Hypocreales	ethyl-Pyrazine	0	0	1
norank_o_Hypocreales	Glycolaldehyde dimer	0	0	1
norank_o_Hypocreales	Hexanal	0	0	1
norank_o_Hypocreales	(methoxymethyl)-Oxirane	0	0	1
norank_o_Hypocreales	trimethyl-Pyrazine	2.49E-40	1.30E-38	1
norank_o_JG30-KF-CM45	1-Heptanol	0	0	1
norank_o_JG30-KF-CM45	2-(1-methylethoxy)-Ethanol	0	0	1
norank_o_JG30-KF-CM45	3-methyl-Uecane	0	0	1
norank_o_JG30-KF-CM45	Acetyl valeryl	0	0	1
norank_o_JG30-KF-CM45	Amyl Nitrite	0	0	1
norank_o_JG30-KF-CM45	diethyl(decyloxy)-Borane	0	0	1
norank_o_JG30-KF-CM45	Oxalic acid-6-ethyloct-3-yl heptyl ester	0	0	1
norank_o_Malasseziales	1,2-Butanediol	1.34E-06	4.66E-05	0.996546
norank_o_Malasseziales	1,3-dihydroxy-2-Propanone	1.34E-06	4.66E-05	0.996546
norank_o_Malasseziales	1-bromo-2-methyl-Decane	1.34E-06	4.66E-05	0.996546
norank_o_Malasseziales	1-Heptadecanamine	1.34E-06	4.66E-05	0.996546
norank_o_Malasseziales	1-hydroxy-2-Propanone	1.34E-06	4.66E-05	0.996546
norank_o_Malasseziales	1-Octen-3-ol	1.34E-06	4.66E-05	0.996546
norank_o_Malasseziales	2,2'-oxybis-Pentane	1.34E-06	4.66E-05	0.996546
norank_o_Malasseziales	2,6-dimethyl-Pyrazine	1.34E-06	4.66E-05	0.996546
norank_o_Malasseziales	2-ethenyl-5-methyl-Pyrazine	1.34E-06	4.66E-05	0.996546
norank_o_Malasseziales	2-ethyl-3,5-dimethyl-Pyrazine	1.34E-06	4.66E-05	0.996546
norank_o_Malasseziales	2-ethyl-5-methyl-Pyrazine	1.34E-06	4.66E-05	0.996546
norank_o_Malasseziales	2-ethyl-6-methyl-Pyrazine	1.34E-06	4.66E-05	0.996546
norank_o_Malasseziales	2-methyl-3-Hexanone	1.34E-06	4.66E-05	0.996546
norank_o_Malasseziales	3,5-diethyl-2-methyl-Pyrazine	1.34E-06	4.66E-05	0.996546
norank_o_Malasseziales	3,8-dimethyl-Decane	1.34E-06	4.66E-05	0.996546
norank_o_Malasseziales	3-hydroxy-2-Butanone	0.000388	0.0104	0.966481
norank_o_Malasseziales	2-(2-butoxyethoxy)-Ethanol	1.34E-06	4.66E-05	0.996546
norank_o_Malasseziales	ethyl-Pyrazine	1.34E-06	4.66E-05	0.996546
norank_o_Malasseziales	Glycolaldehyde dimer	1.34E-06	4.66E-05	0.996546
norank_o_Malasseziales	Hexanal	1.34E-06	4.66E-05	0.996546
norank_o_Malasseziales	(methoxymethyl)-Oxirane	1.34E-06	4.66E-05	0.996546
norank_o_Malasseziales	trimethyl-Pyrazine	1.34E-06	4.66E-05	0.996546
norank_o_Obscuribacterales	Sulfurous acid ethylhexyl isohexyl ester	4.60E-05	0.00134	0.985769
norank_o_Sphingobacteriales	2,4,4-trimethyl-Hexane	0	0	1
norank_o_Sphingobacteriales	3,5-Dimethyl-4-octanone	0	0	1
norank_o_Sphingobacteriales	3-Nonene	0	0	1
norank_o_Sphingobacteriales	Dodecanoic acid ethyl ester	0.001756	0.041348	0.938306

norank_o_Sphingobacteriales	Ethyl 9-hexadecenoate	0	0	1
norank_o_Sphingobacteriales	Oxybis dichloro-Methane	0	0	1
norank_o_Sphingobacteriales	Pentadecanoic acid ethyl ester	0	0	1
norank_o_Tremellales	1,2-Butanediol	1.41E-39	6.85E-38	1
norank_o_Tremellales	1,3-dihydroxy-2-Propanone	0	0	1
norank_o_Tremellales	1-bromo-2-methyl-Decane	0	0	1
norank_o_Tremellales	1-Heptadecanamine	0	0	1
norank_o_Tremellales	1-hydroxy-2-Propanone	0	0	1
norank_o_Tremellales	1-Octen-3-ol	0	0	1
norank_o_Tremellales	2,2'-oxybis-Pentane	0	0	1
norank_o_Tremellales	2,6-dimethyl-Pyrazine	1.41E-39	6.85E-38	1
norank_o_Tremellales	2-ethenyl-5-methyl-Pyrazine	0	0	1
norank_o_Tremellales	2-ethyl-3,5-dimethyl-Pyrazine	1.41E-39	6.85E-38	1
norank_o_Tremellales	2-ethyl-5-methyl-Pyrazine	0	0	1
norank_o_Tremellales	2-ethyl-6-methyl-Pyrazine	1.41E-39	6.85E-38	1
norank_o_Tremellales	2-methyl-3-Hexanone	0	0	1
norank_o_Tremellales	3,5-diethyl-2-methyl-Pyrazine	1.41E-39	6.85E-38	1
norank_o_Tremellales	3,8-dimethyl-Decane	0	0	1
norank_o_Tremellales	3-hydroxy-2-Butanone	0.001337	0.034003	0.944762
norank_o_Tremellales	2-(2-butoxyethoxy)-Ethanol	0	0	1
norank_o_Tremellales	ethyl-Pyrazine	0	0	1
norank_o_Tremellales	Glycolaldehyde dimer	0	0	1
norank_o_Tremellales	Hexanal	0	0	1
norank_o_Tremellales	(methoxymethyl)-Oxirane	0	0	1
norank_o_Tremellales	trimethyl-Pyrazine	2.49E-40	1.30E-38	1
norank_p_Gracilibacteria	2,4,4-trimethyl-Hexane	0	0	1
norank_p_Gracilibacteria	3,5-Dimethyl-4-octanone	0	0	1
norank_p_Gracilibacteria	3-Nonene	0	0	1
norank_p_Gracilibacteria	Dodecanoic acid ethyl ester	0.001756	0.041348	0.938306
norank_p_Gracilibacteria	Ethyl 9-hexadecenoate	0	0	1
norank_p_Gracilibacteria	Oxybis dichloro-Methane	0	0	1
norank_p_Gracilibacteria	Pentadecanoic acid ethyl ester	0	0	1
Novosphingobium	1-ethyl-2-methyl-Cyclopentane	0	0	1
Novosphingobium	1-Pentanol	3.15E-07	1.19E-05	0.998067
Novosphingobium	2-methyl-Propanoic acid pentyl ester	2.74E-08	1.08E-06	0.999272
Novosphingobium	3,5-bis(1,1-dimethylethyl)- Phenol	0	0	1
Novosphingobium	3,7-dimethyl-Nonane	0.000811	0.021364	0.954874
Novosphingobium	3,8-dimethyl-Uecane	0	0	1
Novosphingobium	3-ethoxy-1-Propanol	0	0	1
Novosphingobium	Sulfurous acid octadecyl 2-propyl ester	1.41E-39	6.85E-38	1
Ottowia	2,4,4-trimethyl-Hexane	2.49E-40	1.30E-38	1
Ottowia	3,5-Dimethyl-4-octanone	0	0	1
Ottowia	3-Nonene	0	0	1
Ottowia	Dodecanoic acid ethyl ester	0.001756	0.041348	0.938306

Ottowia	Ethyl 9-hexadecenoate	0	0	1
Ottowia	Oxybis dichloro-Methane	0	0	1
Ottowia	Pentadecanoic acid ethyl ester	0	0	1
Perlucidibaca	Butanedioic acid diethyl ester	0.000404	0.010743	0.965917
Phaselicystis	2,4,4-trimethyl-Hexane	0	0	1
Phaselicystis	3,5-Dimethyl-4-octanone	0	0	1
Phaselicystis	3-Nonene	0	0	1
Phaselicystis	Dodecanoic acid ethyl ester	0.001756	0.041348	0.938306
Phaselicystis	Ethyl 9-hexadecenoate	0	0	1
Phaselicystis	Oxybis dichloro-Methane	0	0	1
Phaselicystis	Pentadecanoic acid ethyl ester	2.49E-40	1.30E-38	1
Phormidium	1-Heptanol	0	0	1
Phormidium	2-(1-methylethoxy)-Ethanol	0	0	1
Phormidium	3-methyl-Uecane	0	0	1
Phormidium	Acetyl valeryl	0	0	1
Phormidium	Amyl Nitrite	0	0	1
Phormidium	diethyl(decyloxy)-Borane	0	0	1
Phormidium	Oxalic acid-6-ethyloct-3-yl heptyl ester	0	0	1
Pichia	1,2-Butanediol	1.60E-10	7.26E-09	0.999907
Pichia	1,3-dihydroxy-2-Propanone	1.60E-10	7.26E-09	0.999907
Pichia	1-bromo-2-methyl-Decane	1.60E-10	7.26E-09	0.999907
Pichia	1-Heptadecanamine	1.60E-10	7.26E-09	0.999907
Pichia	1-hydroxy-2-Propanone	1.60E-10	7.26E-09	0.999907
Pichia	1-Octen-3-ol	1.60E-10	7.26E-09	0.999907
Pichia	2,2'-oxybis-Pentane	1.60E-10	7.26E-09	0.999907
Pichia	2,6-dimethyl-Pyrazine	1.60E-10	7.26E-09	0.999907
Pichia	2-ethenyl-5-methyl-Pyrazine	1.60E-10	7.26E-09	0.999907
Pichia	2-ethyl-3,5-dimethyl-Pyrazine	1.60E-10	7.26E-09	0.999907
Pichia	2-ethyl-5-methyl-Pyrazine	1.60E-10	7.26E-09	0.999907
Pichia	2-ethyl-6-methyl-Pyrazine	1.60E-10	7.26E-09	0.999907
Pichia	2-methyl-3-Hexanone	1.60E-10	7.26E-09	0.999907
Pichia	3,5-diethyl-2-methyl-Pyrazine	1.60E-10	7.26E-09	0.999907
Pichia	3,8-dimethyl-Decane	1.60E-10	7.26E-09	0.999907
Pichia	3-hydroxy-2-Butanone	0.001122	0.029253	0.948534
Pichia	2-(2-butoxyethoxy)-Ethanol	1.60E-10	7.26E-09	0.999907
Pichia	ethyl-Pyrazine	1.60E-10	7.26E-09	0.999907
Pichia	Glycolaldehyde dimer	1.60E-10	7.26E-09	0.999907
Pichia	Hexanal	1.60E-10	7.26E-09	0.999907
Pichia	(methoxymethyl)-Oxirane	1.60E-10	7.26E-09	0.999907
Pichia	trimethyl-Pyrazine	1.60E-10	7.26E-09	0.999907
Prevotellaceae_UCG-001	1-Heptanol	0	0	1
Prevotellaceae_UCG-001	2-(1-methylethoxy)-Ethanol	0	0	1
Prevotellaceae_UCG-001	3-methyl-Uecane	0	0	1
Prevotellaceae_UCG-001	Acetyl valeryl	0	0	1

Prevotellaceae_UCG-001	Amyl Nitrite	0	0	1
Prevotellaceae_UCG-001	diethyl(decyloxy)-Borane	0	0	1
Prevotellaceae_UCG-001	Oxalic acid-6-ethyloct-3-yl heptyl ester	0	0	1
RB41	1-butoxy-2-Propanol	0.000997	0.026046	0.95095
RB41	1-Heptanol	0.000186	0.005264	0.975076
RB41	2-(1-methylethoxy)-Ethanol	0.000186	0.005264	0.975076
RB41	3-methyl-Uecane	0.000186	0.005264	0.975076
RB41	Acetyl valeryl	0.000186	0.005264	0.975076
RB41	Amyl Nitrite	0.000186	0.005264	0.975076
RB41	diethyl(decyloxy)-Borane	0.000186	0.005264	0.975076
RB41	Oxalic acid-6-ethyloct-3-yl heptyl ester	0.000186	0.005264	0.975076
Rhizobium	2,4,4-trimethyl-Hexane	0.000391	0.0104	0.966379
Rhizobium	3,5-Dimethyl-4-octanone	0.000391	0.0104	0.966379
Rhizobium	3-Nonene	0.000391	0.0104	0.966379
Rhizobium	Ethyl 9-hexadecenoate	0.000391	0.0104	0.966379
Rhizobium	Oxybis dichloro-Methane	0.000391	0.0104	0.966379
Rhizobium	Pentadecanoic acid ethyl ester	0.000391	0.0104	0.966379
Ruminococcaceae_NK4A214_group	1-Heptanol	0	0	1
Ruminococcaceae_NK4A214_group	2-(1-methylethoxy)-Ethanol	0	0	1
Ruminococcaceae_NK4A214_group	3-methyl-Uecane	2.49E-40	1.30E-38	1
Ruminococcaceae_NK4A214_group	Acetyl valeryl	1.41E-39	6.85E-38	1
Ruminococcaceae_NK4A214_group	Amyl Nitrite	0	0	1
Ruminococcaceae_NK4A214_group	diethyl(decyloxy)-Borane	1.41E-39	6.85E-38	1
Ruminococcaceae_NK4A214_group	Oxalic acid-6-ethyloct-3-yl heptyl ester	2.49E-40	1.30E-38	1
Saccharomycopsis	1,2-Butanediol	1.23E-05	0.000383	0.99161
Saccharomycopsis	1,3-dihydroxy-2-Propanone	1.23E-05	0.000383	0.99161
Saccharomycopsis	1-bromo-2-methyl-Decane	1.23E-05	0.000383	0.99161
Saccharomycopsis	1-Heptadecanamine	1.23E-05	0.000383	0.99161
Saccharomycopsis	1-hydroxy-2-Propanone	1.23E-05	0.000383	0.99161
Saccharomycopsis	1-Octen-3-ol	1.23E-05	0.000383	0.99161
Saccharomycopsis	2,2'-oxybis-Pentane	1.23E-05	0.000383	0.99161
Saccharomycopsis	2,6-dimethyl-Pyrazine	1.23E-05	0.000383	0.99161
Saccharomycopsis	2-ethenyl-5-methyl-Pyrazine	1.23E-05	0.000383	0.99161
Saccharomycopsis	2-ethyl-3,5-dimethyl-Pyrazine	1.23E-05	0.000383	0.99161
Saccharomycopsis	2-ethyl-5-methyl-Pyrazine	1.23E-05	0.000383	0.99161
Saccharomycopsis	2-ethyl-6-methyl-Pyrazine	1.23E-05	0.000383	0.99161
Saccharomycopsis	2-methyl-3-Hexanone	1.23E-05	0.000383	0.99161
Saccharomycopsis	3,5-diethyl-2-methyl-Pyrazine	1.23E-05	0.000383	0.99161
Saccharomycopsis	3,8-dimethyl-Decane	1.23E-05	0.000383	0.99161
Saccharomycopsis	3-hydroxy-2-Butanone	0.000185	0.005264	0.975095
Saccharomycopsis	2-(2-butoxyethoxy)-Ethanol	1.23E-05	0.000383	0.99161
Saccharomycopsis	ethyl-Pyrazine	1.23E-05	0.000383	0.99161
Saccharomycopsis	Glycolaldehyde dimer	1.23E-05	0.000383	0.99161
Saccharomycopsis	Hexanal	1.23E-05	0.000383	0.99161

Saccharomycopsis	(methoxymethyl)-Oxirane	1.23E-05	0.000383	0.99161
Saccharomycopsis	trimethyl-Pyrazine	1.23E-05	0.000383	0.99161
Shinella	2,4,4-trimethyl-Hexane	1.46E-05	0.000449	0.991026
Shinella	3,5-Dimethyl-4-octanone	1.46E-05	0.000449	0.991026
Shinella	3-Nonene	1.46E-05	0.000449	0.991026
Shinella	Ethyl 9-hexadecenoate	1.46E-05	0.000449	0.991026
Shinella	Oxybis dichloro-Methane	1.46E-05	0.000449	0.991026
Shinella	Pentadecanoic acid ethyl ester	1.46E-05	0.000449	0.991026
SM1A02	1-Heptanol	0	0	1
SM1A02	2-(1-methylethoxy)-Ethanol	0	0	1
SM1A02	3-methyl-Uecane	2.49E-40	1.30E-38	1
SM1A02	Acetyl valeryl	1.41E-39	6.85E-38	1
SM1A02	Amyl Nitrite	0	0	1
SM1A02	diethyl(decyloxy)-Borane	1.41E-39	6.85E-38	1
SM1A02	Oxalic acid-6-ethyloct-3-yl heptyl ester	2.49E-40	1.30E-38	1
Solirubrobacter	1-(1-methylethoxy)-2-Propanol	0	0	1
Solirubrobacter	2-ethyl-Heptanoic acid	2.49E-40	1.30E-38	1
Solirubrobacter	2-Isopropyl-5-methyl-1-heptanol	2.49E-40	1.30E-38	1
Solirubrobacter	3-methyl-5-propyl-Nonane	2.49E-40	1.30E-38	1
Solirubrobacter	Benzeneacetic acid ethyl ester	0	0	1
Solirubrobacter	methyl 6-deoxy-alpha-L-Galactopyranoside	0	0	1
Solirubrobacter	Oxalic acid- 2-ethylhexyl hexyl ester	1.41E-39	6.85E-38	1
Solirubrobacter	Sulfurous acid ethylhexyl hexyl ester	0.000681	0.018004	0.957933
Sporacetigenium	1-butoxy-2-Propanol	0.001278	0.032781	0.945755
Sporidiobolus	1,2-Butanediol	0	0	1
Sporidiobolus	1,3-dihydroxy-2-Propanone	0	0	1
Sporidiobolus	1-bromo-2-methyl-Decane	0	0	1
Sporidiobolus	1-Heptadecanamine	0	0	1
Sporidiobolus	1-hydroxy-2-Propanone	0	0	1
Sporidiobolus	1-Octen-3-ol	0	0	1
Sporidiobolus	2,2'-oxybis-Pentane	0	0	1
Sporidiobolus	2,6-dimethyl-Pyrazine	0	0	1
Sporidiobolus	2-ethenyl-5-methyl-Pyrazine	0	0	1
Sporidiobolus	2-ethyl-3,5-dimethyl-Pyrazine	0	0	1
Sporidiobolus	2-ethyl-5-methyl-Pyrazine	0	0	1
Sporidiobolus	2-ethyl-6-methyl-Pyrazine	0	0	1
Sporidiobolus	2-methyl-3-Hexanone	0	0	1
Sporidiobolus	3,5-diethyl-2-methyl-Pyrazine	0	0	1
Sporidiobolus	3,8-dimethyl-Decane	0	0	1
Sporidiobolus	3-hydroxy-2-Butanone	0.001337	0.034003	0.944762
Sporidiobolus	2-(2-butoxyethoxy)-Ethanol	0	0	1
Sporidiobolus	ethyl-Pyrazine	0	0	1
Sporidiobolus	Glycolaldehyde dimer	0	0	1
Sporidiobolus	Hexanal	0	0	1

Sporidiobolus	(methoxymethyl)-Oxirane	0	0	1
Sporidiobolus	trimethyl-Pyrazine	0	0	1
Staphylococcus	3,7-dimethyl-Nonane	0.001167	0.030259	0.947712
Taibaiella	2,4,4-trimethyl-Hexane	2.49E-40	1.30E-38	1
Taibaiella	3,5-Dimethyl-4-octanone	0	0	1
Taibaiella	3-Nonene	0	0	1
Taibaiella	Dodecanoic acid ethyl ester	0.001756	0.041348	0.938306
Taibaiella	Ethyl 9-hexadecenoate	0	0	1
Taibaiella	Oxybis dichloro-Methane	0	0	1
Taibaiella	Pentadecanoic acid ethyl ester	1.41E-39	6.85E-38	1
Thauera	2,4,4-trimethyl-Hexane	0.000224	0.006294	0.973124
Thauera	3,5-Dimethyl-4-octanone	0.000224	0.006294	0.973124
Thauera	3-Nonene	0.000224	0.006294	0.973124
Thauera	Ethyl 9-hexadecenoate	0.000224	0.006294	0.973124
Thauera	Oxybis dichloro-Methane	0.000224	0.006294	0.973124
Thauera	Pentadecanoic acid ethyl ester	0.000224	0.006294	0.973124
Truepera	2,4,4-trimethyl-Hexane	2.49E-40	1.30E-38	1
Truepera	3,5-Dimethyl-4-octanone	0	0	1
Truepera	3-Nonene	0	0	1
Truepera	Dodecanoic acid ethyl ester	0.001756	0.041348	0.938306
Truepera	Ethyl 9-hexadecenoate	0	0	1
Truepera	Oxybis dichloro-Methane	0	0	1
Truepera	Pentadecanoic acid ethyl ester	1.41E-39	6.85E-38	1
Turicibacter	1-butoxy-2-Propanol	0.00116	0.030103	0.947849
unclassified_c_Gammaproteobacteria	2,4,4-trimethyl-Hexane	2.49E-40	1.30E-38	1
unclassified_c_Gammaproteobacteria	3,5-Dimethyl-4-octanone	0	0	1
unclassified_c_Gammaproteobacteria	3-Nonene	0	0	1
unclassified_c_Gammaproteobacteria	Dodecanoic acid ethyl ester	0.001756	0.041348	0.938306
unclassified_c_Gammaproteobacteria	Ethyl 9-hexadecenoate	0	0	1
unclassified_c_Gammaproteobacteria	Oxybis dichloro-Methane	0	0	1
unclassified_c_Gammaproteobacteria	Pentadecanoic acid ethyl ester	1.41E-39	6.85E-38	1
unclassified_f_Comamonadaceae	2,4,4-trimethyl-Hexane	1.41E-39	6.85E-38	1
unclassified_f_Comamonadaceae	3,5-Dimethyl-4-octanone	0	0	1
unclassified_f_Comamonadaceae	3-Nonene	0	0	1
unclassified_f_Comamonadaceae	Dodecanoic acid ethyl ester	0.001756	0.041348	0.938306
unclassified_f_Comamonadaceae	Ethyl 9-hexadecenoate	0	0	1
unclassified_f_Comamonadaceae	Oxybis dichloro-Methane	0	0	1
unclassified_f_Comamonadaceae	Pentadecanoic acid ethyl ester	0	0	1
unclassified_f_Rhodocyclaceae	2,4,4-trimethyl-Hexane	2.49E-40	1.30E-38	1
unclassified_f_Rhodocyclaceae	3,5-Dimethyl-4-octanone	0	0	1
unclassified_f_Rhodocyclaceae	3-Nonene	2.49E-40	1.30E-38	1
unclassified_f_Rhodocyclaceae	Dodecanoic acid ethyl ester	0.001756	0.041348	0.938306
unclassified_f_Rhodocyclaceae	Ethyl 9-hexadecenoate	0	0	1
unclassified_f_Rhodocyclaceae	Oxybis dichloro-Methane	0	0	1

unclassified_f_Rhodocyclaceae	Pentadecanoic acid ethyl ester	2.49E-40	1.30E-38	1
unclassified_f_Sphingobacteriaceae	2,4,4-trimethyl-Hexane	0	0	1
unclassified_f_Sphingobacteriaceae	3,5-Dimethyl-4-octanone	0	0	1
unclassified_f_Sphingobacteriaceae	3-Nonene	0	0	1
unclassified_f_Sphingobacteriaceae	Dodecanoic acid ethyl ester	0.001756	0.041348	0.938306
unclassified_f_Sphingobacteriaceae	Ethyl 9-hexadecenoate	0	0	1
unclassified_f_Sphingobacteriaceae	Oxybis dichloro-Methane	0	0	1
unclassified_f_Sphingobacteriaceae	Pentadecanoic acid ethyl ester	0	0	1
unclassified_o_Chlamydiales	1-butoxy-2-Propanol	6.11E-08	2.30E-06	0.998997
unclassified_o_Hypocreales	1,2-Butanediol	2.07E-05	0.000615	0.989675
unclassified_o_Hypocreales	1,3-dihydroxy-2-Propanone	2.07E-05	0.000615	0.989675
unclassified_o_Hypocreales	1-bromo-2-methyl-Decane	2.07E-05	0.000615	0.989675
unclassified_o_Hypocreales	1-Heptadecanamine	2.07E-05	0.000615	0.989675
unclassified_o_Hypocreales	1-hydroxy-2-Propanone	2.07E-05	0.000615	0.989675
unclassified_o_Hypocreales	1-Octen-3-ol	2.07E-05	0.000615	0.989675
unclassified_o_Hypocreales	2,2'-oxybis-Pentane	2.07E-05	0.000615	0.989675
unclassified_o_Hypocreales	2,6-dimethyl-Pyrazine	2.07E-05	0.000615	0.989675
unclassified_o_Hypocreales	2-ethenyl-5-methyl-Pyrazine	2.07E-05	0.000615	0.989675
unclassified_o_Hypocreales	2-ethyl-3,5-dimethyl-Pyrazine	2.07E-05	0.000615	0.989675
unclassified_o_Hypocreales	2-ethyl-5-methyl-Pyrazine	2.07E-05	0.000615	0.989675
unclassified_o_Hypocreales	2-ethyl-6-methyl-Pyrazine	2.07E-05	0.000615	0.989675
unclassified_o_Hypocreales	2-methyl-3-Hexanone	2.07E-05	0.000615	0.989675
unclassified_o_Hypocreales	3,5-diethyl-2-methyl-Pyrazine	2.07E-05	0.000615	0.989675
unclassified_o_Hypocreales	3,8-dimethyl-Decane	2.07E-05	0.000615	0.989675
unclassified_o_Hypocreales	3-hydroxy-2-Butanone	0.002001	0.046612	0.934945
unclassified_o_Hypocreales	2-(2-butoxyethoxy)-Ethanol	2.07E-05	0.000615	0.989675
unclassified_o_Hypocreales	ethyl-Pyrazine	2.07E-05	0.000615	0.989675
unclassified_o_Hypocreales	Glycolaldehyde dimer	2.07E-05	0.000615	0.989675
unclassified_o_Hypocreales	Hexanal	2.07E-05	0.000615	0.989675
unclassified_o_Hypocreales	(methoxymethyl)-Oxirane	2.07E-05	0.000615	0.989675
unclassified_o_Hypocreales	trimethyl-Pyrazine	2.07E-05	0.000615	0.989675
unclassified_p_Bacteroidetes	2,4,4-trimethyl-Hexane	2.49E-40	1.30E-38	1
unclassified_p_Bacteroidetes	2,4,4-trimethyl-Hexane	1.62E-09	6.91E-08	0.999765
unclassified_p_Bacteroidetes	3,5-Dimethyl-4-octanone	0	0	1
unclassified_p_Bacteroidetes	3,5-Dimethyl-4-octanone	1.62E-09	6.91E-08	0.999765
unclassified_p_Bacteroidetes	3-Nonene	0	0	1
unclassified_p_Bacteroidetes	3-Nonene	1.62E-09	6.91E-08	0.999765
unclassified_p_Bacteroidetes	Dodecanoic acid ethyl ester	0.001756	0.041348	0.938306
unclassified_p_Bacteroidetes	Dodecanoic acid ethyl ester	0.001879	0.044031	0.936587
unclassified_p_Bacteroidetes	Ethyl 9-hexadecenoate	0	0	1
unclassified_p_Bacteroidetes	Ethyl 9-hexadecenoate	1.62E-09	6.91E-08	0.999765
unclassified_p_Bacteroidetes	Oxybis dichloro-Methane	0	0	1
unclassified_p_Bacteroidetes	Oxybis dichloro-Methane	1.62E-09	6.91E-08	0.999765
unclassified_p_Bacteroidetes	Pentadecanoic acid ethyl ester	1.41E-39	6.85E-38	1

unclassified_p_Bacteroidetes	Pentadecanoic acid ethyl ester	1.62E-09	6.91E-08	0.999765
unclassified_p_Chloroflexi	2,4,4-trimethyl-Hexane	0	0	1
unclassified_p_Chloroflexi	3,5-Dimethyl-4-octanone	0	0	1
unclassified_p_Chloroflexi	3-Nonene	0	0	1
unclassified_p_Chloroflexi	Dodecanoic acid ethyl ester	0.001756	0.041348	0.938306
unclassified_p_Chloroflexi	Ethyl 9-hexadecenoate	0	0	1
unclassified_p_Chloroflexi	Oxybis dichloro-Methane	0	0	1
unclassified_p_Chloroflexi	Pentadecanoic acid ethyl ester	2.49E-40	1.30E-38	1
unclassified_p_Planctomycetes	1-Heptanol	0	0	1
unclassified_p_Planctomycetes	2-(1-methylethoxy)-Ethanol	0	0	1
unclassified_p_Planctomycetes	3-methyl-Uecane	2.49E-40	1.30E-38	1
unclassified_p_Planctomycetes	Acetyl valeryl	1.41E-39	6.85E-38	1
unclassified_p_Planctomycetes	Amyl Nitrite	0	0	1
unclassified_p_Planctomycetes	diethyl(decyloxy)-Borane	1.41E-39	6.85E-38	1
unclassified_p_Planctomycetes	Oxalic acid-6-ethyloct-3-yl heptyl ester	2.49E-40	1.30E-38	1
vadinBC27_wastewater-sludge_group	2,4,4-trimethyl-Hexane	2.49E-40	1.30E-38	1
vadinBC27_wastewater-sludge_group	3,5-Dimethyl-4-octanone	0	0	1
vadinBC27_wastewater-sludge_group	3-Nonene	2.49E-40	1.30E-38	1
vadinBC27_wastewater-sludge_group	Dodecanoic acid ethyl ester	0.001756	0.041348	0.938306
vadinBC27_wastewater-sludge_group	Ethyl 9-hexadecenoate	0	0	1
vadinBC27_wastewater-sludge_group	Oxybis dichloro-Methane	0	0	1
vadinBC27_wastewater-sludge_group	Pentadecanoic acid ethyl ester	2.49E-40	1.30E-38	1

Table S8. Microbial significance level based on O2PLS model

Microorganism	Quantiles a	Quantiles b	Significance
unclassified_o_Saccharomycetales	0.1559	-0.0104	upper quantiles
Lactococcus	-0.157	-0.0293	lower quantiles
Pediococcus	-0.1294	-0.0468	lower quantiles
Aspergillus	-0.1552	-0.0302	lower quantiles
Cladosporium	-0.1557	-0.0308	lower quantiles
Cochliobolus	-0.1438	-0.02	lower quantiles
norank_k_Fungi	-0.1563	-0.018	lower quantiles
norank_o_Hypocreales	-0.1559	-0.031	lower quantiles
unclassified_o_Hypocreales	-0.1479	-0.0398	lower quantiles
norank_o_Malasseziales	-0.1558	-0.0294	lower quantiles
Pichia	-0.156	-0.0305	lower quantiles
Saccharomycopsis	-0.1554	-0.028	lower quantiles
Sporidiobolus	-0.1559	-0.031	lower quantiles
norank_o_Tremellales	-0.1559	-0.031	lower quantiles

Table S9. Correlation of significant microbiota with flavor compounds during CRW fermentation process

Microorganism	Flavor compounds	P value	P adjust	Correlation
Aspergillus	(methoxymethyl)-Oxirane	1.05E-06	1.08E-05	0.996876359
Aspergillus	1,2-Butanediol	1.05E-06	1.08E-05	0.996876359
Aspergillus	1,3-dihydroxy-2-Propanone	1.05E-06	1.08E-05	0.996876359
Aspergillus	1-bromo-2-methyl-Decane	1.05E-06	1.08E-05	0.996876359
Aspergillus	1-Heptadecanamine	1.05E-06	1.08E-05	0.996876359
Aspergillus	1-hydroxy-2-Propanone	1.05E-06	1.08E-05	0.996876359
Aspergillus	1-Octen-3-ol	1.05E-06	1.08E-05	0.996876359
Aspergillus	2-(2-butoxyethoxy)-Ethanol	1.05E-06	1.08E-05	0.996876359
Aspergillus	2,2'-oxybis-Pentane	1.05E-06	1.08E-05	0.996876359
Aspergillus	2,6-dimethyl-Pyrazine	1.05E-06	1.08E-05	0.996876359
Aspergillus	2-ethenyl-5-methyl-Pyrazine	1.05E-06	1.08E-05	0.996876359
Aspergillus	2-ethyl-3,5-dimethyl-Pyrazine	1.05E-06	1.08E-05	0.996876359
Aspergillus	2-ethyl-5-methyl-Pyrazine	1.05E-06	1.08E-05	0.996876359
Aspergillus	2-ethyl-6-methyl-Pyrazine	1.05E-06	1.08E-05	0.996876359
Aspergillus	2-methyl-3-Hexanone	1.05E-06	1.08E-05	0.996876359
Aspergillus	3,5-diethyl-2-methyl-Pyrazine	1.05E-06	1.08E-05	0.996876359
Aspergillus	3,8-dimethyl-Decane	1.05E-06	1.08E-05	0.996876359
Aspergillus	3-hydroxy-2-Butanone	0.000386	0.002483	0.966553268
Aspergillus	ethyl-Pyrazine	1.05E-06	1.08E-05	0.996876359
Aspergillus	Glycolaldehyde dimer	1.05E-06	1.08E-05	0.996876359
Aspergillus	Hexanal	1.05E-06	1.08E-05	0.996876359
Aspergillus	trimethyl-Pyrazine	1.05E-06	1.08E-05	0.996876359
Cladosporium	(methoxymethyl)-Oxirane	8.24E-10	1.19E-08	0.999820932
Cladosporium	1,2-Butanediol	8.24E-10	1.19E-08	0.999820932
Cladosporium	1,3-dihydroxy-2-Propanone	8.24E-10	1.19E-08	0.999820932
Cladosporium	1-bromo-2-methyl-Decane	8.24E-10	1.19E-08	0.999820932
Cladosporium	1-Heptadecanamine	8.24E-10	1.19E-08	0.999820932
Cladosporium	1-hydroxy-2-Propanone	8.24E-10	1.19E-08	0.999820932
Cladosporium	1-Octen-3-ol	8.24E-10	1.19E-08	0.999820932
Cladosporium	2-(2-butoxyethoxy)-Ethanol	8.24E-10	1.19E-08	0.999820932
Cladosporium	2,2'-oxybis-Pentane	8.24E-10	1.19E-08	0.999820932
Cladosporium	2,6-dimethyl-Pyrazine	8.24E-10	1.19E-08	0.999820932
Cladosporium	2-ethenyl-5-methyl-Pyrazine	8.24E-10	1.19E-08	0.999820932
Cladosporium	2-ethyl-3,5-dimethyl-Pyrazine	8.24E-10	1.19E-08	0.999820932
Cladosporium	2-ethyl-5-methyl-Pyrazine	8.24E-10	1.19E-08	0.999820932
Cladosporium	2-ethyl-6-methyl-Pyrazine	8.24E-10	1.19E-08	0.999820932
Cladosporium	2-methyl-3-Hexanone	8.24E-10	1.19E-08	0.999820932
Cladosporium	3,5-diethyl-2-methyl-Pyrazine	8.24E-10	1.19E-08	0.999820932
Cladosporium	3,8-dimethyl-Decane	8.24E-10	1.19E-08	0.999820932
Cladosporium	3-hydroxy-2-Butanone	0.001024	0.006531	0.950415059
Cladosporium	ethyl-Pyrazine	8.24E-10	1.19E-08	0.999820932
Cladosporium	Glycolaldehyde dimer	8.24E-10	1.19E-08	0.999820932

Cladosporium	Hexanal	8.24E-10	1.19E-08	0.999820932
Cladosporium	trimethyl-Pyrazine	8.24E-10	1.19E-08	0.999820932
Cochliobolus	(methoxymethyl)-Oxirane	0.005286	0.027751	0.903392682
Cochliobolus	1,2-Butanediol	0.005286	0.027751	0.903392682
Cochliobolus	1,3-dihydroxy-2-Propanone	0.005286	0.027751	0.903392682
Cochliobolus	1-bromo-2-methyl-Decane	0.005286	0.027751	0.903392682
Cochliobolus	1-Heptadecanamine	0.005286	0.027751	0.903392682
Cochliobolus	1-hydroxy-2-Propanone	0.005286	0.027751	0.903392682
Cochliobolus	1-Octen-3-ol	0.005286	0.027751	0.903392682
Cochliobolus	2-(2-butoxyethoxy)-Ethanol	0.005286	0.027751	0.903392682
Cochliobolus	2,2'-oxybis-Pentane	0.005286	0.027751	0.903392682
Cochliobolus	2,6-dimethyl-Pyrazine	0.005286	0.027751	0.903392682
Cochliobolus	2-ethenyl-5-methyl-Pyrazine	0.005286	0.027751	0.903392682
Cochliobolus	2-ethyl-3,5-dimethyl-Pyrazine	0.005286	0.027751	0.903392682
Cochliobolus	2-ethyl-5-methyl-Pyrazine	0.005286	0.027751	0.903392682
Cochliobolus	2-ethyl-6-methyl-Pyrazine	0.005286	0.027751	0.903392682
Cochliobolus	2-methyl-3-Hexanone	0.005286	0.027751	0.903392682
Cochliobolus	3,5-diethyl-2-methyl-Pyrazine	0.005286	0.027751	0.903392682
Cochliobolus	3,8-dimethyl-Decane	0.005286	0.027751	0.903392682
Cochliobolus	3-hydroxy-2-Butanone	0.000107	0.000767	0.980038378
Cochliobolus	9,12-Octadecadienoic acid ethyl ester	0.003797	0.022224	-0.91559973
Cochliobolus	ethyl-Pyrazine	0.005286	0.027751	0.903392682
Cochliobolus	Glycolaldehyde dimer	0.005286	0.027751	0.903392682
Cochliobolus	Hexanal	0.005286	0.027751	0.903392682
Cochliobolus	trimethyl-Pyrazine	0.005286	0.027751	0.903392682
Lactococcus	(methoxymethyl)-Oxirane	2.80E-08	3.37E-07	0.99926573
Lactococcus	1,2-Butanediol	2.80E-08	3.37E-07	0.99926573
Lactococcus	1,3-dihydroxy-2-Propanone	2.80E-08	3.37E-07	0.99926573
Lactococcus	1-bromo-2-methyl-Decane	2.80E-08	3.37E-07	0.99926573
Lactococcus	1-Heptadecanamine	2.80E-08	3.37E-07	0.99926573
Lactococcus	1-hydroxy-2-Propanone	2.80E-08	3.37E-07	0.99926573
Lactococcus	1-Octen-3-ol	2.80E-08	3.37E-07	0.99926573
Lactococcus	2-(2-butoxyethoxy)-Ethanol	2.80E-08	3.37E-07	0.99926573
Lactococcus	2,2'-oxybis-Pentane	2.80E-08	3.37E-07	0.99926573
Lactococcus	2,6-dimethyl-Pyrazine	2.80E-08	3.37E-07	0.99926573
Lactococcus	2-ethenyl-5-methyl-Pyrazine	2.80E-08	3.37E-07	0.99926573
Lactococcus	2-ethyl-3,5-dimethyl-Pyrazine	2.80E-08	3.37E-07	0.99926573
Lactococcus	2-ethyl-5-methyl-Pyrazine	2.80E-08	3.37E-07	0.99926573
Lactococcus	2-ethyl-6-methyl-Pyrazine	2.80E-08	3.37E-07	0.99926573
Lactococcus	2-methyl-3-Hexanone	2.80E-08	3.37E-07	0.99926573
Lactococcus	3,5-diethyl-2-methyl-Pyrazine	2.80E-08	3.37E-07	0.99926573
Lactococcus	3,8-dimethyl-Decane	2.80E-08	3.37E-07	0.99926573
Lactococcus	3-hydroxy-2-Butanone	0.00178	0.011119	0.937971641
Lactococcus	ethyl-Pyrazine	2.80E-08	3.37E-07	0.99926573

Lactococcus	Glycolaldehyde dimer	2.80E-08	3.37E-07	0.99926573
Lactococcus	Hexanal	2.80E-08	3.37E-07	0.99926573
Lactococcus	trimethyl-Pyrazine	2.80E-08	3.37E-07	0.99926573
norank_k_Fungi	(methoxymethyl)-Oxirane	0.000272	0.001765	0.97094707
norank_k_Fungi	1,2-Butanediol	0.000272	0.001765	0.97094707
norank_k_Fungi	1,3-dihydroxy-2-Propanone	0.000272	0.001765	0.97094707
norank_k_Fungi	1-bromo-2-methyl-Decane	0.000272	0.001765	0.97094707
norank_k_Fungi	1-Heptadecanamine	0.000272	0.001765	0.97094707
norank_k_Fungi	1-hydroxy-2-Propanone	0.000272	0.001765	0.97094707
norank_k_Fungi	1-Octen-3-ol	0.000272	0.001765	0.97094707
norank_k_Fungi	2-(2-butoxyethoxy)-Ethanol	0.000272	0.001765	0.97094707
norank_k_Fungi	2,2'-oxybis-Pentane	0.000272	0.001765	0.97094707
norank_k_Fungi	2,6-dimethyl-Pyrazine	0.000272	0.001765	0.97094707
norank_k_Fungi	2-ethenyl-5-methyl-Pyrazine	0.000272	0.001765	0.97094707
norank_k_Fungi	2-ethyl-3,5-dimethyl-Pyrazine	0.000272	0.001765	0.97094707
norank_k_Fungi	2-ethyl-5-methyl-Pyrazine	0.000272	0.001765	0.97094707
norank_k_Fungi	2-ethyl-6-methyl-Pyrazine	0.000272	0.001765	0.97094707
norank_k_Fungi	2-methyl-3-Hexanone	0.000272	0.001765	0.97094707
norank_k_Fungi	3,5-diethyl-2-methyl-Pyrazine	0.000272	0.001765	0.97094707
norank_k_Fungi	3,8-dimethyl-Decane	0.000272	0.001765	0.97094707
norank_k_Fungi	3-hydroxy-2-Butanone	0.000274	0.001773	0.970849511
norank_k_Fungi	ethyl-Pyrazine	0.000272	0.001765	0.97094707
norank_k_Fungi	Glycolaldehyde dimer	0.000272	0.001765	0.97094707
norank_k_Fungi	Hexanal	0.000272	0.001765	0.97094707
norank_k_Fungi	trimethyl-Pyrazine	0.000272	0.001765	0.97094707
norank_o_Hypocreales	(methoxymethyl)-Oxirane	0	0	1
norank_o_Hypocreales	1,2-Butanediol	0	0	1
norank_o_Hypocreales	1,3-dihydroxy-2-Propanone	0	0	1
norank_o_Hypocreales	1-bromo-2-methyl-Decane	0	0	1
norank_o_Hypocreales	1-Heptadecanamine	0	0	1
norank_o_Hypocreales	1-hydroxy-2-Propanone	0	0	1
norank_o_Hypocreales	1-Octen-3-ol	0	0	1
norank_o_Hypocreales	2-(2-butoxyethoxy)-Ethanol	0	0	1
norank_o_Hypocreales	2,2'-oxybis-Pentane	0	0	1
norank_o_Hypocreales	2,6-dimethyl-Pyrazine	0	0	1
norank_o_Hypocreales	2-ethenyl-5-methyl-Pyrazine	0	0	1
norank_o_Hypocreales	2-ethyl-3,5-dimethyl-Pyrazine	0	0	1
norank_o_Hypocreales	2-ethyl-5-methyl-Pyrazine	0	0	1
norank_o_Hypocreales	2-ethyl-6-methyl-Pyrazine	0	0	1
norank_o_Hypocreales	2-methyl-3-Hexanone	0	0	1
norank_o_Hypocreales	3,5-diethyl-2-methyl-Pyrazine	0	0	1
norank_o_Hypocreales	3,8-dimethyl-Decane	0	0	1
norank_o_Hypocreales	3-hydroxy-2-Butanone	0.001337	0.008387	0.9447621
norank_o_Hypocreales	ethyl-Pyrazine	0	0	1

norank_o_Hypocreales	Glycolaldehyde dimer	0	0	1
norank_o_Hypocreales	Hexanal	0	0	1
norank_o_Hypocreales	trimethyl-Pyrazine	0	0	1
norank_o_Malasseziales	(methoxymethyl)-Oxirane	1.34E-06	1.21E-05	0.99654644
norank_o_Malasseziales	1,2-Butanediol	1.34E-06	1.21E-05	0.99654644
norank_o_Malasseziales	1,3-dihydroxy-2-Propanone	1.34E-06	1.21E-05	0.99654644
norank_o_Malasseziales	1-bromo-2-methyl-Decane	1.34E-06	1.21E-05	0.99654644
norank_o_Malasseziales	1-Heptadecanamine	1.34E-06	1.21E-05	0.99654644
norank_o_Malasseziales	1-hydroxy-2-Propanone	1.34E-06	1.21E-05	0.99654644
norank_o_Malasseziales	1-Octen-3-ol	1.34E-06	1.21E-05	0.99654644
norank_o_Malasseziales	2-(2-butoxyethoxy)-Ethanol	1.34E-06	1.21E-05	0.99654644
norank_o_Malasseziales	2,2'-oxybis-Pentane	1.34E-06	1.21E-05	0.99654644
norank_o_Malasseziales	2,6-dimethyl-Pyrazine	1.34E-06	1.21E-05	0.99654644
norank_o_Malasseziales	2-ethenyl-5-methyl-Pyrazine	1.34E-06	1.21E-05	0.99654644
norank_o_Malasseziales	2-ethyl-3,5-dimethyl-Pyrazine	1.34E-06	1.21E-05	0.99654644
norank_o_Malasseziales	2-ethyl-5-methyl-Pyrazine	1.34E-06	1.21E-05	0.99654644
norank_o_Malasseziales	2-ethyl-6-methyl-Pyrazine	1.34E-06	1.21E-05	0.99654644
norank_o_Malasseziales	2-methyl-3-Hexanone	1.34E-06	1.21E-05	0.99654644
norank_o_Malasseziales	3,5-diethyl-2-methyl-Pyrazine	1.34E-06	1.21E-05	0.99654644
norank_o_Malasseziales	3,8-dimethyl-Decane	1.34E-06	1.21E-05	0.99654644
norank_o_Malasseziales	3-hydroxy-2-Butanone	0.000388	0.002486	0.966481243
norank_o_Malasseziales	ethyl-Pyrazine	1.34E-06	1.21E-05	0.99654644
norank_o_Malasseziales	Glycolaldehyde dimer	1.34E-06	1.21E-05	0.99654644
norank_o_Malasseziales	Hexanal	1.34E-06	1.21E-05	0.99654644
norank_o_Malasseziales	trimethyl-Pyrazine	1.34E-06	1.21E-05	0.99654644
norank_o_Tremellales	(methoxymethyl)-Oxirane	0	0	1
norank_o_Tremellales	1,2-Butanediol	0	0	1
norank_o_Tremellales	1,3-dihydroxy-2-Propanone	0	0	1
norank_o_Tremellales	1-bromo-2-methyl-Decane	0	0	1
norank_o_Tremellales	1-Heptadecanamine	0	0	1
norank_o_Tremellales	1-hydroxy-2-Propanone	0	0	1
norank_o_Tremellales	1-Octen-3-ol	0	0	1
norank_o_Tremellales	2-(2-butoxyethoxy)-Ethanol	0	0	1
norank_o_Tremellales	2,2'-oxybis-Pentane	0	0	1
norank_o_Tremellales	2,6-dimethyl-Pyrazine	0	0	1
norank_o_Tremellales	2-ethenyl-5-methyl-Pyrazine	0	0	1
norank_o_Tremellales	2-ethyl-3,5-dimethyl-Pyrazine	0	0	1
norank_o_Tremellales	2-ethyl-5-methyl-Pyrazine	0	0	1
norank_o_Tremellales	2-ethyl-6-methyl-Pyrazine	0	0	1
norank_o_Tremellales	2-methyl-3-Hexanone	0	0	1
norank_o_Tremellales	3,5-diethyl-2-methyl-Pyrazine	0	0	1
norank_o_Tremellales	3,8-dimethyl-Decane	0	0	1
norank_o_Tremellales	3-hydroxy-2-Butanone	0.001337	0.008387	0.9447621
norank_o_Tremellales	ethyl-Pyrazine	0	0	1

norank_o_Tremellales	Glycolaldehyde dimer	0	0	1
norank_o_Tremellales	Hexanal	0	0	1
norank_o_Tremellales	trimethyl-Pyrazine	0	0	1
Pediococcus	3-hydroxy-2-Butanone	0.002906	0.01801	0.924299865
Pichia	(methoxymethyl)-Oxirane	1.60E-10	2.88E-09	0.999907061
Pichia	1,2-Butanediol	1.60E-10	2.88E-09	0.999907061
Pichia	1,3-dihydroxy-2-Propanone	1.60E-10	2.88E-09	0.999907061
Pichia	1-bromo-2-methyl-Decane	1.60E-10	2.88E-09	0.999907061
Pichia	1-Heptadecanamine	1.60E-10	2.88E-09	0.999907061
Pichia	1-hydroxy-2-Propanone	1.60E-10	2.88E-09	0.999907061
Pichia	1-Octen-3-ol	1.60E-10	2.88E-09	0.999907061
Pichia	2-(2-butoxyethoxy)-Ethanol	1.60E-10	2.88E-09	0.999907061
Pichia	2,2'-oxybis-Pentane	1.60E-10	2.88E-09	0.999907061
Pichia	2,6-dimethyl-Pyrazine	1.60E-10	2.88E-09	0.999907061
Pichia	2-ethenyl-5-methyl-Pyrazine	1.60E-10	2.88E-09	0.999907061
Pichia	2-ethyl-3,5-dimethyl-Pyrazine	1.60E-10	2.88E-09	0.999907061
Pichia	2-ethyl-5-methyl-Pyrazine	1.60E-10	2.88E-09	0.999907061
Pichia	2-ethyl-6-methyl-Pyrazine	1.60E-10	2.88E-09	0.999907061
Pichia	2-methyl-3-Hexanone	1.60E-10	2.88E-09	0.999907061
Pichia	3,5-diethyl-2-methyl-Pyrazine	1.60E-10	2.88E-09	0.999907061
Pichia	3,8-dimethyl-Decane	1.60E-10	2.88E-09	0.999907061
Pichia	3-hydroxy-2-Butanone	0.001122	0.007131	0.94853448
Pichia	ethyl-Pyrazine	1.60E-10	2.88E-09	0.999907061
Pichia	Glycolaldehyde dimer	1.60E-10	2.88E-09	0.999907061
Pichia	Hexanal	1.60E-10	2.88E-09	0.999907061
Pichia	trimethyl-Pyrazine	1.60E-10	2.88E-09	0.999907061
Saccharomycopsis	(methoxymethyl)-Oxirane	1.23E-05	9.86E-05	0.991610366
Saccharomycopsis	1,2-Butanediol	1.23E-05	9.86E-05	0.991610366
Saccharomycopsis	1,3-dihydroxy-2-Propanone	1.23E-05	9.86E-05	0.991610366
Saccharomycopsis	1-bromo-2-methyl-Decane	1.23E-05	9.86E-05	0.991610366
Saccharomycopsis	1-Heptadecanamine	1.23E-05	9.86E-05	0.991610366
Saccharomycopsis	1-hydroxy-2-Propanone	1.23E-05	9.86E-05	0.991610366
Saccharomycopsis	1-Octen-3-ol	1.23E-05	9.86E-05	0.991610366
Saccharomycopsis	2-(2-butoxyethoxy)-Ethanol	1.23E-05	9.86E-05	0.991610366
Saccharomycopsis	2,2'-oxybis-Pentane	1.23E-05	9.86E-05	0.991610366
Saccharomycopsis	2,6-dimethyl-Pyrazine	1.23E-05	9.86E-05	0.991610366
Saccharomycopsis	2-ethenyl-5-methyl-Pyrazine	1.23E-05	9.86E-05	0.991610366
Saccharomycopsis	2-ethyl-3,5-dimethyl-Pyrazine	1.23E-05	9.86E-05	0.991610366
Saccharomycopsis	2-ethyl-5-methyl-Pyrazine	1.23E-05	9.86E-05	0.991610366
Saccharomycopsis	2-ethyl-6-methyl-Pyrazine	1.23E-05	9.86E-05	0.991610366
Saccharomycopsis	2-methyl-3-Hexanone	1.23E-05	9.86E-05	0.991610366
Saccharomycopsis	3,5-diethyl-2-methyl-Pyrazine	1.23E-05	9.86E-05	0.991610366
Saccharomycopsis	3,8-dimethyl-Decane	1.23E-05	9.86E-05	0.991610366
Saccharomycopsis	3-hydroxy-2-Butanone	0.000185	0.001323	0.975095283

Saccharomycopsis	ethyl-Pyrazine	1.23E-05	9.86E-05	0.991610366
Saccharomycopsis	Glycolaldehyde dimer	1.23E-05	9.86E-05	0.991610366
Saccharomycopsis	Hexanal	1.23E-05	9.86E-05	0.991610366
Saccharomycopsis	trimethyl-Pyrazine	1.23E-05	9.86E-05	0.991610366
Sporidiobolus	(methoxymethyl)-Oxirane	0	0	1
Sporidiobolus	1,2-Butanediol	0	0	1
Sporidiobolus	1,3-dihydroxy-2-Propanone	0	0	1
Sporidiobolus	1-bromo-2-methyl-Decane	0	0	1
Sporidiobolus	1-Heptadecanamine	0	0	1
Sporidiobolus	1-hydroxy-2-Propanone	0	0	1
Sporidiobolus	1-Octen-3-ol	0	0	1
Sporidiobolus	2-(2-butoxyethoxy)-Ethanol	0	0	1
Sporidiobolus	2,2'-oxybis-Pentane	0	0	1
Sporidiobolus	2,6-dimethyl-Pyrazine	0	0	1
Sporidiobolus	2-ethenyl-5-methyl-Pyrazine	0	0	1
Sporidiobolus	2-ethyl-3,5-dimethyl-Pyrazine	0	0	1
Sporidiobolus	2-ethyl-5-methyl-Pyrazine	0	0	1
Sporidiobolus	2-ethyl-6-methyl-Pyrazine	0	0	1
Sporidiobolus	2-methyl-3-Hexanone	0	0	1
Sporidiobolus	3,5-diethyl-2-methyl-Pyrazine	0	0	1
Sporidiobolus	3,8-dimethyl-Decane	0	0	1
Sporidiobolus	3-hydroxy-2-Butanone	0.001337	0.008387	0.9447621
Sporidiobolus	ethyl-Pyrazine	0	0	1
Sporidiobolus	Glycolaldehyde dimer	0	0	1
Sporidiobolus	Hexanal	0	0	1
Sporidiobolus	trimethyl-Pyrazine	0	0	1
unclassified_o_Hypocreales	(methoxymethyl)-Oxirane	2.07E-05	0.000149	0.989675383
unclassified_o_Hypocreales	1,2-Butanediol	2.07E-05	0.000149	0.989675383
unclassified_o_Hypocreales	1,3-dihydroxy-2-Propanone	2.07E-05	0.000149	0.989675383
unclassified_o_Hypocreales	1-bromo-2-methyl-Decane	2.07E-05	0.000149	0.989675383
unclassified_o_Hypocreales	1-Heptadecanamine	2.07E-05	0.000149	0.989675383
unclassified_o_Hypocreales	1-hydroxy-2-Propanone	2.07E-05	0.000149	0.989675383
unclassified_o_Hypocreales	1-Octen-3-ol	2.07E-05	0.000149	0.989675383
unclassified_o_Hypocreales	2-(2-butoxyethoxy)-Ethanol	2.07E-05	0.000149	0.989675383
unclassified_o_Hypocreales	2,2'-oxybis-Pentane	2.07E-05	0.000149	0.989675383
unclassified_o_Hypocreales	2,6-dimethyl-Pyrazine	2.07E-05	0.000149	0.989675383
unclassified_o_Hypocreales	2-ethenyl-5-methyl-Pyrazine	2.07E-05	0.000149	0.989675383
unclassified_o_Hypocreales	2-ethyl-3,5-dimethyl-Pyrazine	2.07E-05	0.000149	0.989675383
unclassified_o_Hypocreales	2-ethyl-5-methyl-Pyrazine	2.07E-05	0.000149	0.989675383
unclassified_o_Hypocreales	2-ethyl-6-methyl-Pyrazine	2.07E-05	0.000149	0.989675383
unclassified_o_Hypocreales	2-methyl-3-Hexanone	2.07E-05	0.000149	0.989675383
unclassified_o_Hypocreales	3,5-diethyl-2-methyl-Pyrazine	2.07E-05	0.000149	0.989675383
unclassified_o_Hypocreales	3,8-dimethyl-Decane	2.07E-05	0.000149	0.989675383
unclassified_o_Hypocreales	3-hydroxy-2-Butanone	0.002001	0.012454	0.934945024

unclassified_o_Hypocreales	ethyl-Pyrazine	2.07E-05	0.000149	0.989675383
unclassified_o_Hypocreales	Glycolaldehyde dimer	2.07E-05	0.000149	0.989675383
unclassified_o_Hypocreales	Hexanal	2.07E-05	0.000149	0.989675383
unclassified_o_Hypocreales	trimethyl-Pyrazine	2.07E-05	0.000149	0.989675383
unclassified_o_Saccharomycetales	(methoxymethyl)-Oxirane	0.00391	0.022224	-0.91458234
unclassified_o_Saccharomycetales	1,2-Butanediol	0.00391	0.022224	-0.91458234
unclassified_o_Saccharomycetales	1,3-dihydroxy-2-Propanone	0.00391	0.022224	-0.91458234
unclassified_o_Saccharomycetales	1-bromo-2-methyl-Decane	0.00391	0.022224	-0.91458234
unclassified_o_Saccharomycetales	1-Heptadecanamine	0.00391	0.022224	-0.91458234
unclassified_o_Saccharomycetales	1-hydroxy-2-Propanone	0.00391	0.022224	-0.91458234
unclassified_o_Saccharomycetales	1-Octen-3-ol	0.00391	0.022224	-0.91458234
unclassified_o_Saccharomycetales	2-(2-butoxyethoxy)-Ethanol	0.00391	0.022224	-0.91458234
unclassified_o_Saccharomycetales	2,2'-oxybis-Pentane	0.00391	0.022224	-0.91458234
unclassified_o_Saccharomycetales	2,6-dimethyl-Pyrazine	0.00391	0.022224	-0.91458234
unclassified_o_Saccharomycetales	2-ethenyl-5-methyl-Pyrazine	0.00391	0.022224	-0.91458234
unclassified_o_Saccharomycetales	2-ethyl-3,5-dimethyl-Pyrazine	0.00391	0.022224	-0.91458234
unclassified_o_Saccharomycetales	2-ethyl-5-methyl-Pyrazine	0.00391	0.022224	-0.91458234
unclassified_o_Saccharomycetales	2-ethyl-6-methyl-Pyrazine	0.00391	0.022224	-0.91458234
unclassified_o_Saccharomycetales	2-methyl-3-Hexanone	0.00391	0.022224	-0.91458234
unclassified_o_Saccharomycetales	3,5-diethyl-2-methyl-Pyrazine	0.00391	0.022224	-0.91458234
unclassified_o_Saccharomycetales	3,8-dimethyl-Decane	0.00391	0.022224	-0.91458234
unclassified_o_Saccharomycetales	3-hydroxy-2-Butanone	0.00621	0.032492	-0.89680825
unclassified_o_Saccharomycetales	9,12-Octadecadienoic acid ethyl ester	0.003993	0.022614	0.913842848
unclassified_o_Saccharomycetales	ethyl-Pyrazine	0.00391	0.022224	-0.91458234
unclassified_o_Saccharomycetales	Glycolaldehyde dimer	0.00391	0.022224	-0.91458234
unclassified_o_Saccharomycetales	Hexanal	0.00391	0.022224	-0.91458234
unclassified_o_Saccharomycetales	trimethyl-Pyrazine	0.00391	0.022224	-0.91458234
